# Macrophage SELENOP controls neutrophil homeostasis through redox stress regulation in Crohn's disease

**DOI:** 10.1016/j.redox.2026.104377

**Published:** 2026-09-09

**Authors:** Honggang Wang, Juan Wang, Wenliang Jiang, Chao Cheng, Shaoqi Cheng, Shuqiang Fu, Yi Zhang, Cuixia Liu, Jing Sun, Jie Zhao

**Affiliations:** a Department of General Surgery, The Affiliated Taizhou People's Hospital of Nanjing Medical University, Taizhou School of Clinical Medicine, Nanjing Medical University, 366 Taihu Road, Taizhou, Jiangsu, China; b Department of Gastroenterology, The Affiliated Taizhou People's Hospital of Nanjing Medical University, Taizhou School of Clinical Medicine, Nanjing Medical University, 366 Taihu Road, Taizhou, Jiangsu, China; c Department of Gastroenterology, The First Affiliated Hospital with Nanjing Medical University, Nanjing, Jiangsu, China; d Department of Gastroenterology, The Affiliated Wuxi People's Hospital of Nanjing Medical University, Wuxi, China; e Department of Gastrointestinal Surgery, The Affiliated Changzhou No.2 People's Hospital of Nanjing Medical University, The Third Affiliated Hospital of Nanjing Medical University, Changzhou Medical Center, Nanjing Medical University, Changzhou, China

**Keywords:** Crohn's disease, SELENOP, Oxidative stress, Macrophage–neutrophil crosstalk, Ubiquitination

## Abstract

**Background:**

Neutrophil accumulation and oxidative injury are prominent features of Crohn's disease (CD). This study aimed to investigate whether macrophage-derived SELENOP functions as a redox checkpoint in macrophage–neutrophil crosstalk during CD.

**Methods:**

Inflamed and non-inflamed colon tissues from CD patients were examined by single-cell RNA sequencing. SELENOP function was assessed in macrophages, macrophage–neutrophil co-cultures and inflammation models. Mechanistic studies included loss- and gain-of-function assays, co-immunoprecipitation, ubiquitination assays, and rescue experiments. *In vivo* validation was performed using myeloid-specific SELENOP-deficient mice with TNBS-induced colitis and SELENOP-overexpressing IL-10-deficient mice.

**Results:**

SELENOP was selectively diminished in colon macrophages from inflamed CD lesions and its reduction was associated with more severe disease activity and postoperative recurrence. Functionally, SELENOP reduction in macrophages acquired a pro-oxidative and hyperinflammatory state through SESN2/NRF2 antioxidant signaling and CAP1. TRIP12 promoted ubiquitin-dependent SELENOP degradation, whereas CREB1 and SMARCA4 cooperatively activated SELENOP transcription. SELENOP-deficient macrophages altered redox and cADPR-associated metabolic signaling, thereby inducing neutrophil oxidative stress, apoptosis and NET formation. Myeloid SELENOP deletion aggravated experimental TNBS-induced colitis, while SELENOP overexpression alleviated IL-10-deficient colitis in a macrophage-dependent manner.

**Conclusions:**

Our findings identify macrophage-derived SELENOP as a redox checkpoint that restrains pathogenic macrophage–neutrophil communication in CD. Targeting the macrophage SELENOP may recalibrate oxidative stress-driven colonic inflammation.

## 1 Introduction

Crohn's disease (CD) is a chronic, relapsing inflammatory disorder of the gastrointestinal tract characterized by segmental and transmural inflammation [1]. Its pathogenesis involves a complex interplay among genetic susceptibility, environmental factors, intestinal microbiota dysbiosis, and aberrant immune responses [2]. Although corticosteroids, immunomodulators, and biologics targeting tumor necrosis factor-alpha (TNF-α), integrins, and interleukin-23 (IL-23) have substantially improved disease management, major challenges remain, including incomplete remission, treatment resistance, disease recurrence, and long-term complications [3,4].

Intestinal macrophages are central regulators of gut immune homeostasis, contributing to pathogen clearance, cytokine production, and epithelial repair [5]. Under homeostatic conditions, they exhibit a tolerogenic phenotype characterized by restrained inflammatory signaling and robust phagocytic activity [6]. In CD, however, macrophages acquire aberrant inflammatory features, including impaired polarization, defective microbial clearance, and persistent activation of innate immune pathways [7,8]. Increasing evidence indicates that metabolic reprogramming and oxidative stress critically shape macrophage function during intestinal inflammation [9,10]. Oxidative stress, resulting from an imbalance between reactive oxygen species (ROS) generation and antioxidant defenses, is now recognized as a key driver of CD pathogenesis by contributing to epithelial injury, barrier dysfunction, and exaggerated inflammatory responses [11,12]. In particular, excessive ROS accumulation in intestinal macrophages promotes the production of pro-inflammatory mediators and favors M1-like polarization, thereby amplifying mucosal inflammation [13,14].

Neutrophils are also key contributors to intestinal inflammation in CD. Beyond their antimicrobial functions, activated neutrophils produce large amounts of ROS and release neutrophil extracellular traps (NETs), which, when excessive or dysregulated, can aggravate tissue injury and perpetuate inflammation [15]. Importantly, macrophages and neutrophils do not act in isolation; rather, their reciprocal communication through cytokines, metabolites, and redox-associated signals is increasingly recognized as a critical mechanism shaping intestinal immune responses [16]. However, the molecular mechanisms by which macrophage-intrinsic oxidative stress regulates neutrophil activation and NET formation in CD remain poorly understood.

SELENOP (selenoprotein P) is a major selenium-containing extracellular protein that functions not only as the primary selenium transporter in mammals but also as an important regulator of cellular redox homeostasis [17,18]. By delivering selenium to recipient cells, SELENOP supports the biosynthesis and activity of essential antioxidant selenoproteins, including glutathione peroxidases and thioredoxin reductases, thereby contributing to the maintenance of intracellular redox balance [19,20]. Beyond its role in selenium metabolism, accumulating evidence suggests that SELENOP participates in oxidative stress regulation and inflammatory responses. Altered SELENOP expression has been associated with metabolic disorders, chronic inflammation, and redox imbalance, indicating its potential role as a link between selenium metabolism and immune regulation [21,22]. However, whether and how SELENOP regulates macrophage oxidative homeostasis and inflammatory responses in intestinal inflammation remains largely unknown. Here, we demonstrate that SELENOP acts as a critical regulator of macrophage oxidative stress and metabolic homeostasis in CD. Macrophage-specific loss of SELENOP enhances oxidative stress and drives the production of metabolite-derived signals that promote neutrophil oxidative stress and NET formation, thereby exacerbating intestinal inflammation. These findings identify a previously unrecognized SELENOP-dependent macrophage–neutrophil axis in CD and provide new insight into therapeutic strategies aimed at restoring redox balance and modulating innate immune crosstalk.

## 2 Materials and methods

### 2.1 Patient samples

Colon tissue specimens (including inflamed and non-inflamed) were collected from 60 CD patients undergoing colectomy or ileocolectomy. A total of 4 samples were used for scRNA-seq and 56 for validation. Informed consent was obtained, and the study followed ethical guidelines approved by the Ethics Committee (No. 2023-SR-115). Inclusion criteria were based on a previous report, and demographic data (age, gender, and disease location) are summarized in Table S2. Preoperative inflammatory markers (e.g., C-reactive protein (CRP), simplified endoscopic activity score for Crohn's disease (SES-CD) [23], Crohn's disease activity index (CDAI) [24]) were assessed using established methods. Colon tissues were obtained from surgically resected specimens, frozen in liquid nitrogen, and stored at −80 °C for further studies.

### 2.2 scRNA-seq

ScRNA sequencing was performed by Genechem (Shanghai, China) using the BD Rhapsody WTA platform. Raw reads were aligned to human (GRCh38) or mouse (mm10) reference genomes with annotations from GENCODE and Ensembl. UMI count matrices were analyzed in Scanpy (v1.8). Low-quality cells with reduced gene counts or >10% mitochondrial gene expression were excluded. The remaining data were normalized and log-transformed. Highly variable genes were selected for downstream analysis. Dimensionality reduction was conducted using principal component analysis (PCA), followed by clustering and visualization with UMAP. Differentially expressed genes were identified using the Wilcoxon rank-sum test in Seurat, with adjusted *P* < 0.05 and |log_2_ fold change| > 0.25 as significance thresholds. Functional enrichment analysis, including gene ontology (GO) and Kyoto encyclopedia of genes and genomes (KEGG) pathways, was performed using g:Profiler2 [25].

### 2.3 Co-immunoprecipitation (Co-IP) and mass spectrometry (MS)

Co-immunoprecipitation assays were performed using standard procedures [26]. Cells were lysed in IP lysis buffer, and protein lysates were collected after centrifugation. Protein quality was verified prior to immunoprecipitation. Magnetic Protein A/G beads were washed and incubated with specific antibodies, followed by incubation with protein lysates to capture target protein complexes. After washing, bound proteins were eluted in loading buffer and denatured at 95 °C for 10 min. Eluted samples were subjected to Western blotting or further proteomic analysis.

For mass spectrometry, protein samples were separated by silver staining according to the manufacturer's protocol. Peptides were analyzed using an UltiMate 3000 RSLCnano system coupled with a Q Exactive HF mass spectrometer. Raw data were processed using MaxQuant (v1.6.6.0) [27] and searched against the UniProt human protein database [28]. Proteins with a fold change > 2 and supported by at least two unique peptides were defined as significantly enriched.

### 2.4 Animal experiments

IL-10 knockout (KO) mice on a C57BL/6 background were maintained under specific pathogen-free (SPF) conditions. Twelve-week-old male wild-type (WT) and IL-10 KO mice were randomly assigned to four groups (n = 6 per group): WT control group; KO mice receiving AAV9-CD68-vector; IL-10 KO mice treated with macrophage-specific SELENOP overexpression via AAV9-CD68-SELENOP (1 × 10^11^ vg in 200 μL, single enema); and IL-10 KO mice receiving AAV9-CD68-SELENOP combined with clodronate (200 μL, intraperitoneal injection). Mice were euthanized one week after treatment, and proximal colon tissues were collected.

For TNBS-induced colitis, SELENOP^flox/flox^ and SELENOP^Lyz2−Cre^ mice were sensitized with 1% TNBS, followed by intracolonic administration of 150 μL 2.5% TNBS after 18 h fasting under isoflurane anesthesia. Body weight and survival were monitored daily. Mice were sacrificed on day 7, and colon tissues were harvested for downstream analyses. All procedures were approved by the institutional ethics committee of Jiangsu Hanjiang Biotechnology (No. HJSW-25090501).

### 2.5 Disease activity index (DAI)

Disease activity was scored based on stool consistency, fecal blood, and rectal prolapse [29]. Each parameter was graded according to severity, and cumulative scores were calculated to obtain the DAI.

### 2.6 *In vivo* neutrophil activity

Neutrophil activity was assessed by bioluminescence imaging following intraperitoneal injection of the MPO-sensitive probe L-012 (20 mM, 4 μL/g body weight) [30]. Imaging was performed 20 min post-injection, and signal intensity was quantified as background-corrected photon flux.

### 2.7 MPO activity assay

Colon tissues were homogenized, and myeloperoxidase activity was measured using a commercial colorimetric assay kit according to the manufacturer's instructions. Absorbance at 460 nm was recorded using a microplate reader, and MPO activity (U/g tissue) was calculated based on reaction kinetics [31].

### 2.8 Histological analysis

Colon length was measured immediately after sacrifice. Tissues were fixed in 10% buffered formalin, paraffin-embedded, and sectioned (6 μm). Sections were stained with haematoxylin and eosin or AB-PAS. Histological scoring was independently performed by two blinded observers using established criteria [32].

### 2.9 Immunofluorescence staining

Colon tissues were fixed in 4% paraformaldehyde, embedded, and sectioned (4 μm). After deparaffinization and rehydration, antigen retrieval was performed in EDTA buffer. Sections or cell samples were blocked with 5% BSA for 30 min and incubated with primary antibodies overnight at 4 °C. After washing, samples were incubated with HRP-conjugated secondary antibodies and subjected to tyramide signal amplification. Nuclei were counterstained with DAPI, and images were acquired for analysis.

### 2.10 Western blotting

Total proteins were extracted using RIPA buffer supplemented with protease and phosphatase inhibitors, followed by centrifugation at 12,000 × g for 15 min at 4 °C. Protein concentrations were determined using a BCA assay. Equal amounts of protein (15 μg) were separated by SDS-PAGE and transferred to PVDF membranes. Membranes were blocked with 5% milk and incubated with primary antibodies overnight at 4 °C, followed by HRP-conjugated secondary antibodies for 2 h at room temperature. Signals were detected using enhanced chemiluminescence and quantified using ImageJ, with normalization to β-actin or H3 (nuclear). The antibodies were presented in Table S1.

### 2.11 RNA extraction and qRT-PCR

Total RNA was extracted using TRIzol reagent according to the manufacturer's instructions. RNA concentration and purity were assessed spectrophotometrically. For each sample, 1 μg RNA was reverse-transcribed into cDNA in a 20 μL reaction volume. Quantitative PCR was performed using SYBR Green master mix on a real-time PCR system. Each 20 μL reaction contained 10 μL master mix, 0.4 μL of each primer, and 2 μL cDNA template. The cycling conditions were 95 °C for 30 s, followed by 40 cycles of 95 °C for 5 s and 60 °C for 30 s. Gene expression levels were normalized to GAPDH and calculated using the 2^−ΔΔCt^ method. All reactions were performed in triplicate, with primer sequences listed in Table S3.

### 2.12 Transmission electron microscopy

Tight junction ultrastructure was examined by transmission electron microscopy. Colon tissues were fixed in 4% glutaraldehyde and post-fixed in 1% osmium tetroxide. Samples were dehydrated through graded ethanol, embedded in Epon812 resin, and sectioned into ultrathin slices (∼70 nm). Sections were stained with uranyl acetate and lead citrate and imaged using a Hitachi H600 transmission electron microscope.

### 2.13 Intestinal permeability assays

#### 2.13.1 *Ex vivo* permeability [33]

Colonic mucosa was mounted in oxygenated Krebs-buffered chambers as previously described. Both compartments contained Krebs solution supplemented with 10 mM fructose. Paracellular permeability was assessed by measuring mucosal-to-serosal flux of [^3^H]-mannitol (1 mM, 370 kBq/mL). Transepithelial potential difference was continuously recorded, and tissue resistance (Ω·cm^2^) was calculated according to Ohm's law [34].

#### 2.13.2 In vitro barrier function assays

##### 2.13.2.1 TEER measurement

Barrier integrity of epithelial monolayers was assessed by transepithelial electrical resistance (TEER). Caco-2 cells (STR-authenticated) were cultured for 18–21 days to allow full differentiation. Monolayers were then treated with conditioned media derived from LPS/ATP-stimulated SELENOP^+^/^+^ or SELENOP^−/−^ macrophages for 48 h. TEER was measured at 0, 24, and 48 h using a Millicell ERS-2 volt-ohm meter.

Prior to measurement, medium was replaced with pre-warmed HBSS containing 5 mM HEPES. Measurements were performed in triplicate, and values were corrected by subtracting blank inserts and normalized to membrane area (0.33 cm^2^). Results were expressed relative to baseline.

#### 2.13.3 FITC-dextran permeability [35]

Paracellular permeability was further evaluated using FITC-dextran (4 kDa). In vitro, flux across epithelial monolayers was quantified. In vivo, mice were orally gavaged with FITC-dextran (600 mg/kg), and serum fluorescence was measured at 4 h post-administration (excitation 485 nm, emission 528 nm).

### 2.14 Flow cytometry

#### 2.14.1 Apoptosis analysis

Epithelial cell apoptosis was assessed using Annexin V-FITC/propidium iodide (PI) staining. Cells were incubated with 5 μL Annexin V-FITC and 10 μL PI and analyzed by flow cytometry (Beckman Coulter EpicsAltra). Early apoptotic cells were defined as Annexin V^+^/PI^−^, and late apoptotic cells as Annexin V^+^/PI^+^. Experiments were performed in triplicate.

#### 2.14.2 Intracellular ROS measurement

Intracellular ROS levels were quantified using DCFH-DA staining. THP-1-derived macrophages under the indicated treatment conditions were exposed to LPS/ATP to induce inflammation, with untreated cells included as the control group. After treatment, cells were incubated with DCFH-DA working solution in the dark at 37 °C for 30 min. Subsequently, the cells were washed with serum-free medium or PBS to remove excess probe, collected, and analyzed by flow cytometry to determine intracellular ROS production. The fluorescence intensity of DCF was recorded, and the mean fluorescence intensity (MFI) was used for quantitative analysis of ROS levels.

### 2.15 Cell transfection

THP-1-derived macrophages were transfected using Lipofectamine 2000 according to standard protocols. Gene silencing of SELENOP, TRIP12, SMARCA4, and CREB1 was achieved using specific shRNA or siRNAs, while SELENOP and TRIP12 overexpression was performed using a pcDNA3.1 expression vector. Sequences of all constructs are provided in Table S4.

### 2.16 Ubiquitination assay

SELENOP ubiquitination was assessed in HEK293T cells following modulation of TRIP12 expression. Cells were transfected with SELENOP overexpression or knockdown constructs and treated with MG132 to inhibit proteasomal degradation. Cell lysates were subjected to immunoprecipitation using an anti-SELENOP antibody, followed by SDS-PAGE and immunoblotting with an anti-ubiquitin antibody.

For in vitro validation, HEK293T cells were co-transfected with Myc-tagged SELENOP, His-tagged ubiquitin, and HA-tagged TRIP12. After MG132 treatment, SELENOP was immunoprecipitated using an anti-Myc antibody, and ubiquitinated forms were detected by immunoblotting with an anti-His antibody. Input samples were analyzed in parallel to confirm protein expression.

### 2.17 Cycloheximide (CHX) chase assay

SELENOP protein stability was evaluated using a cycloheximide chase assay [36]. HEK293T cells were transfected with TRIP12 overexpression, TRIP12-specific siRNA, or corresponding controls. After 24 h, cells were treated with cycloheximide (100 μg/mL) to block protein synthesis. SELENOP protein levels were monitored at indicated time points by Western blotting.

### 2.18 Live/dead staining

Cell viability of intestinal epithelial cells was assessed using calcein AM/propidium iodide (PI) staining. Cells were incubated with staining solution prepared in PBS for 15 min at room temperature in the dark, followed by fluorescence microscopy imaging. Live cells were identified by calcein-positive staining, whereas dead cells were PI-positive.

### 2.19 Detection of oxidative stress biomarker

Colon tissues were homogenized in ice-cold saline at a ratio of 1:9 (w/v) to obtain 10% (w/v) homogenates. Cells were washed twice with pre-chilled PBS and lysed in cold PBS on ice. The lysates were centrifuged at 4 °C for 10 min, and the resulting supernatants were collected for analysis. The levels of thiobarbituric acid reactive substances (TBARS), as an indicator of lipid peroxidation and malondialdehyde (MDA)-related aldehydic products, and the activity of superoxide dismutase (SOD) were determined using commercial assay kits (Nanjing Jiancheng Bioengineering Institute, China) according to the manufacturer's instructions. Absorbance was measured using a microplate reader, and the results were normalized to total protein concentration where applicable.

### 2.20 Lactate dehydrogenase (LDH) release detection

For the LDH release assay, cells were plated in 96-well plates at 4 × 10^4^ cells per well and allowed to adhere overnight. Following the indicated treatments, the culture supernatants were harvested and assayed using the CytoTox96 cytotoxicity kit (Promega) in accordance with the manufacturer's protocol. LDH release was expressed as a percentage and used to evaluate membrane damage-associated cytotoxicity, based on the release of the cytosolic enzyme LDH into the supernatant after disruption of plasma membrane integrity.

### 2.21 Chromatin immunoprecipitation (ChIP)-qPCR

Chromatin immunoprecipitation was performed using a commercial ChIP kit following standard procedures [37]. Briefly, 1 × 10^7^ cells per sample were processed to isolate ChIP-enriched DNA. Binding of aryl hydrocarbon receptor (AHR) to the SELENOP promoter was assessed by qPCR. Putative AHR-binding sites within the SELENOP promoter were identified using the JASPAR database, and corresponding primers were designed for ChIP-qPCR analysis (Table S3).

### 2.22 Luciferase reporter assay

SELENOP promoter fragments containing CREB1-binding regions were amplified from genomic DNA and cloned into the pGL4.17 luciferase reporter vector. HEK293T cells were co-transfected with reporter constructs and a Renilla luciferase plasmid for normalization. Following transfection, cells were serum-starved and stimulated with BMP6 (5 nM) overnight. Luciferase activity was measured using a dual-luciferase assay system and normalized to Renilla activity.

### 2.23 Statistical analysis

Statistical analyses were performed using SPSS (version 19.0) and GraphPad Prism (version 8.0). Paired t-tests were used to compare inflamed and non-inflamed regions from the same clinical samples. Comparisons between two independent groups were conducted using two-tailed unpaired t-tests with Welch's correction. Multiple group comparisons were analyzed by one-way ANOVA followed by Tukey's post hoc test. Survival in the TNBS mouse model and associations between SELENOP expression and endoscopic recurrence were evaluated using Kaplan–Meier analysis with the log-rank test. Data are presented as mean ± SD. Correlations between SELENOP expression and CD-related inflammatory indices were assessed using Pearson's correlation analysis. A two-sided *P* value < 0.05 was considered statistically significant.

## 3 Results

### 3.1 Macrophage-specific downregulation of SELENOP associated with CD inflammation

**Fig. 1:**
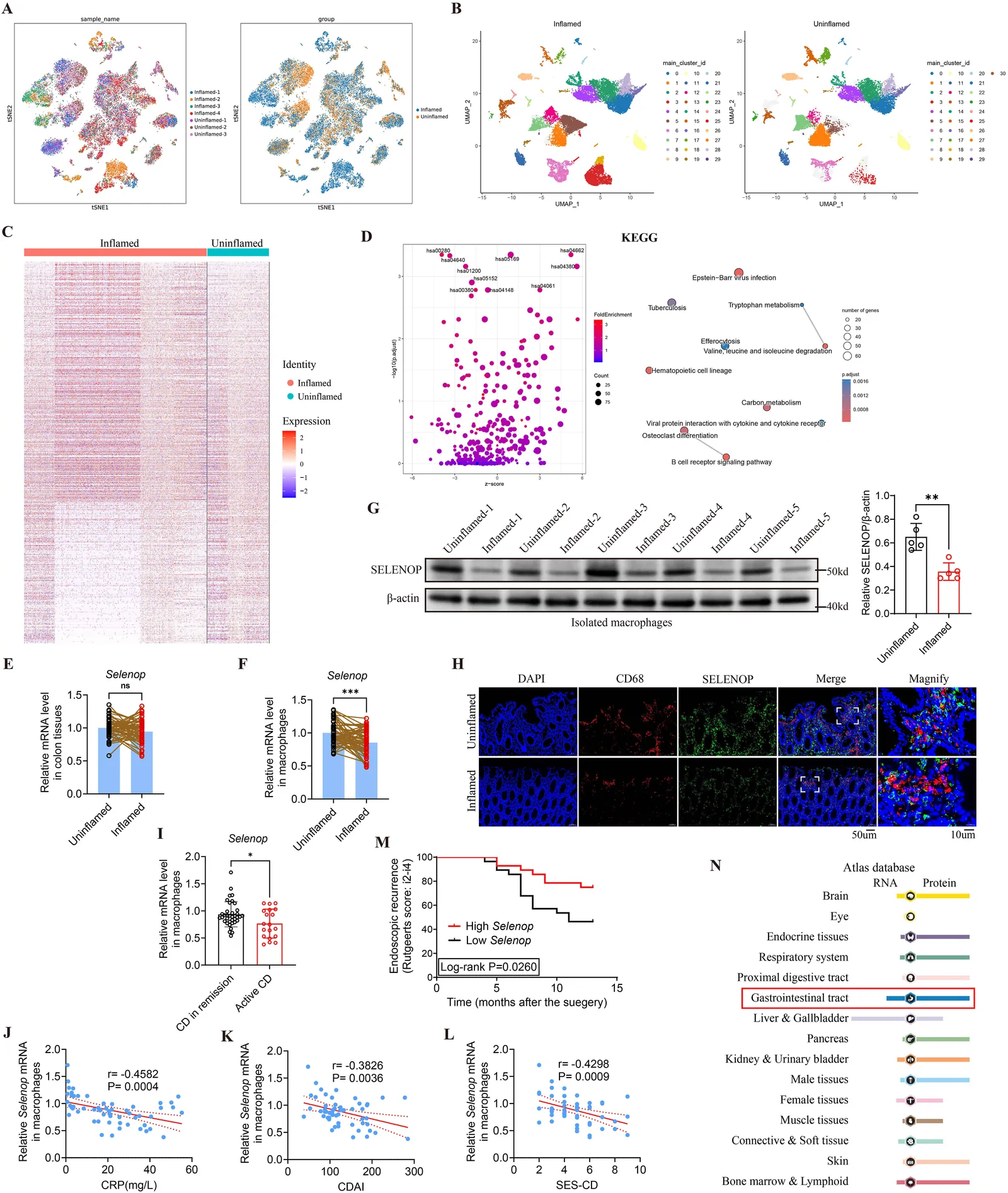
Macrophage-specific downregulation of SELENOP associated with CD inflammation (A, B) t-SNE and UMAP visualization of scRNA-seq data from inflamed and uninflamed colonic tissues, showing distinct cellular clusters and their distributions by sample and inflammatory status. (C) Heatmap of differentially expressed genes in macrophages from inflamed versus uninflamed colon tissues. (D) KEGG enrichment analysis of macrophage differentially expressed genes, highlighting pathways associated with inflammation, and oxidative stress. (E, F) Relative *Selenop* mRNA expression in whole colon tissues (E) and isolated macrophages (F) from uninflamed and inflamed colon samples. (G) Representative Western blot and quantification of SELENOP protein expression in isolated macrophages. (H) Representative immunofluorescence staining for CD68 and SELENOP in uninflamed and inflamed colonic tissues. Nuclei were counterstained with DAPI. Scale bars: 50 μm and 10 μm. (I) Relative *Selenop* mRNA expression in macrophages from patients in remission and patients with active CD. (J–L) Correlations between macrophage *Selenop* mRNA expression and CRP (J), CDAI (K), and SES-CD (L). (M) Kaplan–Meier analysis of postoperative endoscopic recurrence according to macrophage Selenop mRNA expression. (N) RNA and protein expression profiles of SELENOP across human tissues from the Human Protein Atlas database. scRNA-seq, single-cell RNA sequencing; t-SNE, t-distributed stochastic neighbour embedding; UMAP, uniform manifold approximation and projection; KEGG, Kyoto Encyclopedia of Genes and Genomes; DAPI, 4′,6-diamidino-2-phenylindole; CD, Crohn's disease; CRP, C-reactive protein; CDAI, Crohn's disease activity index; SES-CD, Simple Endoscopic Score for Crohn's Disease. Data are presented as mean ± SD. Statistical analysis was performed using paired Student's *t*-test (E, F) or Welch's two-tailed unpaired *t*-test (G, I). For two-group comparisons, correlations were assessed by Spearman's correlation analysis (J, K, L), endoscopic recurrence was analyzed by the Kaplan-Meier method with log-rank test (M). ns, not significant; *P < 0.05; **P < 0.01; ***P < 0.001.

**Fig. S1:**
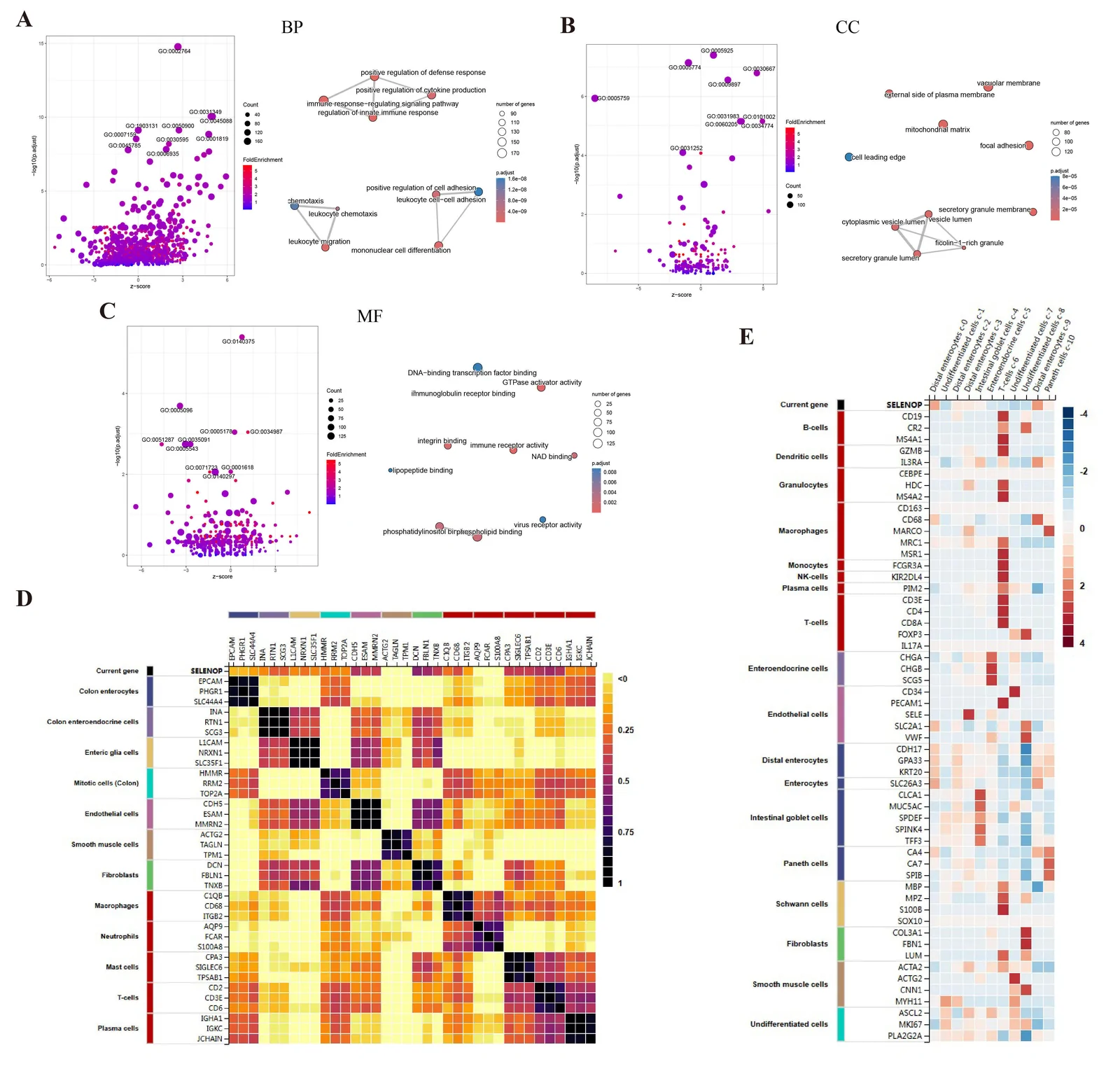
GO enrichment of macrophage differentially expressed genes and cell-type-specific expression pattern of SELENOP by Atlas database. (A–C) GO enrichment analysis of macrophage differentially expressed genes in inflamed versus uninflamed colon tissues, showing the top enriched terms in the biological process (BP), cellular component (CC), and molecular function (MF) categories. (D-E) Heatmap showing the expression of SELENOP across annotated intestinal cell populations.

To characterize cellular heterogeneity, scRNA sequencing data were visualized using t-SNE (Fig. 1A) and UMAP (Fig. 1B) dimensionality reduction, indicating distinct clusters and distributions across inflamed and uninflamed colon tissues. Differential gene expression analysis revealed widespread transcriptional alterations in macrophages between inflamed and uninflamed samples (Fig. 1C). GO (Fig. S1A–S1C) and KEGG (Fig. 1D) enrichment analysis of differentially expressed genes in macrophages showed significant enrichment of pathways related to inflammation and oxidative stress, including Viral protein interaction with cytokine and cytokine receptor, Tryptophan metabolism, and Efferocytosis. Among the differentially expressed genes, *Selenop* was ranked among the top candidates. As a gene encoding a selenium-containing antioxidant protein, SELENOP is closely associated with inflammation and oxidative stress [38], and therefore drew our particular attention.

While no significant difference was observed at the whole-tissue level (Fig. 1E), *Selenop* expression was significantly reduced in macrophages from inflamed tissues (Fig. 1F), indicating cell type-specific regulation. Consistently, Western blot (Fig. 1G) and immunofluorescence staining (Fig. 1H) analysis of isolated macrophages confirmed decreased SELENOP protein levels in inflamed samples. Further analysis showed that macrophage *Selenop* expression was significantly decreased in active CD patients compared to those in remission (Fig. 1I). Correlation analyses revealed that macrophage *Selenop* expression was negatively associated with systemic inflammation markers, including CRP (Fig. 1J), CDAI (Fig. 1K), and SES-CD (Fig. 1L), suggesting a potential anti-inflammatory role. Kaplan–Meier survival analysis demonstrated that patients with low *Selenop* expression exhibited significantly higher endoscopic recurrence rates following surgery (Fig. 1M), indicating its potential prognostic value. Atlas database showed that SELENOP is broadly expressed across tissues, with notable enrichment in the gastrointestinal tract at both RNA and protein levels (Fig. 1N,Fig. S1D, and Fig. S1E).

### 3.2 SELENOP deficiency exacerbates inflammatory and oxidative injury in macrophages via SESN2

**Fig. 2:**
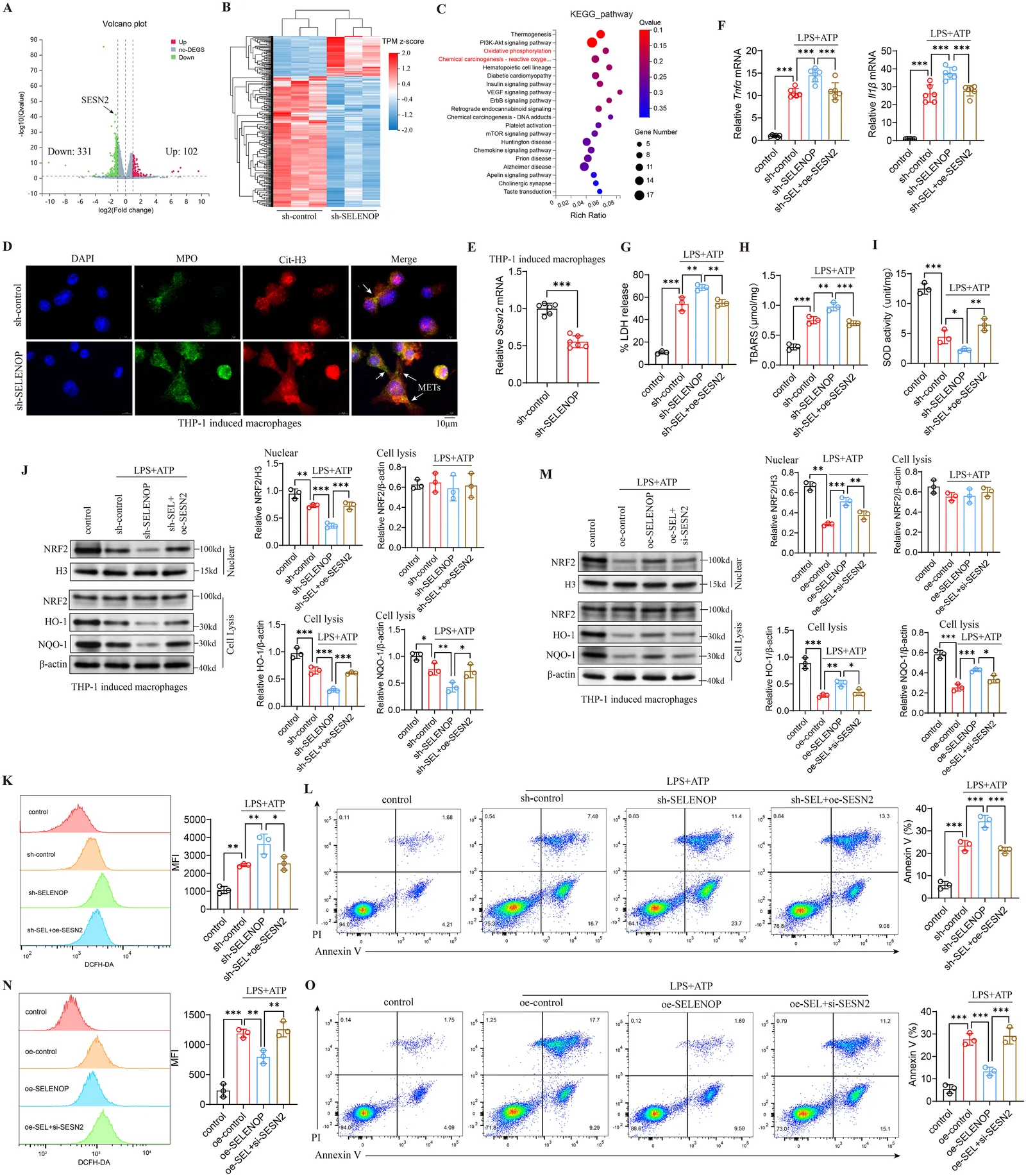
SELENOP deficiency aggravates inflammatory and oxidative injury in macrophages through SESN2/NRF2 signalling. (A) Volcano plot and (B) heatmap showing differentially expressed genes in THP-1-derived macrophages transduced with sh-control or sh-SELENOP. (C) KEGG pathway enrichment analysis of differentially expressed genes, showing enrichment of oxidative stress -related pathways. (D) Representative immunofluorescence staining for MPO and Cit-H3 in sh-control and sh-SELENOP macrophages. Arrows indicate extracellular traps. Scale bar, 10 μm (E) Relative *Sesn2* mRNA expression in sh-control and sh-SELENOP macrophages. (F) Relative *Tnfα* and *Il1β* mRNA expression in macrophages treated with LPS/ATP. (G–I) LDH release (G), TBARS levels (H), and SOD activity (I) in macrophages treated with LPS/ATP. (J) Representative immunoblot analysis and quantification of NRF2/HO-1/NQO1 pathway in sh-control, sh-SELENOP, and sh-SELENOP + oe-SESN2 macrophages treated with LPS/ATP. H3 and β-actin were used as loading controls for nuclear and total protein extracts, respectively. (K) Representative DCFH-DA flow cytometric analysis and quantification of intracellular ROS levels in macrophages treated with LPS/ATP. (L) Representative Annexin V/PI flow cytometric analysis and quantification of Annexin V-positive cells in macrophages treated with LPS/ATP. (M) Representative immunoblot analysis and quantification of NRF2/HO-1/NQO1 pathway in oe-control, oe-SELENOP, and oe-SELENOP + si-SESN2 macrophages treated with LPS/ATP. H3 and β-actin were used as loading controls for nuclear and total protein extracts, respectively. (N) Representative DCFH-DA flow cytometric analysis and quantification of intracellular ROS levels in macrophages treated with LPS/ATP. (O) Representative Annexin V/PI flow cytometric analysis and quantification of Annexin V-positive cells in macrophages treated with LPS/ATP. MPO, myeloperoxidase; Cit-H3, citrullinated histone H3; DAPI, 4′,6-diamidino-2-phenylindole; LPS, lipopolysaccharide; ATP, adenosine triphosphate; LDH, lactate dehydrogenase; TBARS, thiobarbituric acid reactive substances; SOD, superoxide dismutase; ROS, reactive oxygen species; NRF2, nuclear factor erythroid 2-related factor 2; HO-1, heme oxygenase 1; NQO-1, NAD(P)H quinone dehydrogenase 1. Data are presented as mean ± SD. Statistical analysis was performed using Welch's two-tailed unpaired *t*-test (E), or one-way ANOVA followed by Tukey's post hoc test for the rest. ns, not significant; *P < 0.05; **P < 0.01; ***P < 0.001.

**Fig. S2:**
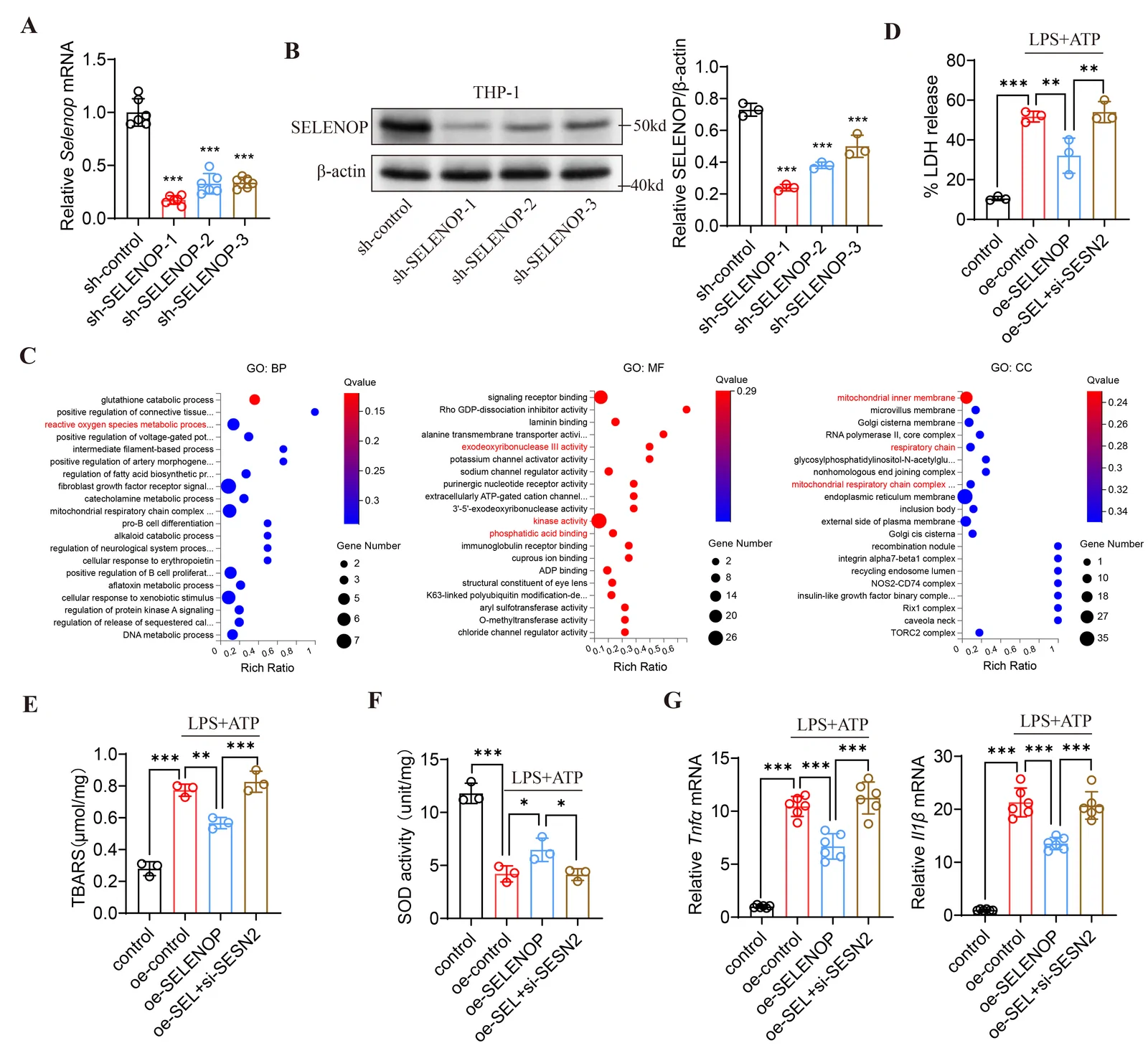
SELENOP regulates oxidative stress and inflammatory responses in macrophages via SESN2. (A–B) qRT-PCR and WB analyses showing the knockdown efficiency of SELENOP in THP-1–derived macrophages. (C) GO enrichment analysis of differentially expressed genes, showing enriched terms in BP, MF, and CC categories. (D) LDH release in control, oe-SELENOP, and oe-SEL + si-SESN2 macrophages following LPS + ATP stimulation. (E) TBRAS levels under the indicated conditions. (F) SOD activity in macrophages. (G) qRT-PCR analysis of Tnfα and Il1β expression under the indicated conditions. SELENOP, selenoprotein P; SESN2, sestrin 2; qRT-PCR, quantitative real-time PCR; WB, Western blot; GO, Gene Ontology; BP, biological process; MF, molecular function; CC, cellular component; LDH, lactate dehydrogenase; LPS, lipopolysaccharide; ATP, adenosine triphosphate; TBARS, thiobarbituric acid reactive substances; SOD, superoxide dismutase; TNFα, tumor necrosis factor alpha; IL-1β, interleukin-1 beta. Data are presented as mean ± SD. Statistical analysis was performed using Welch's two-tailed unpaired *t*-test (E), or one-way ANOVA followed by Tukey's post hoc test for the rest. ns, not significant; *P < 0.05; **P < 0.01; ***P < 0.001.

To further investigate the biological role of SELENOP, we first performed RNA-seq following validation of knockdown efficiency (Fig. S2A and S2B). Differential expression analysis of sh-SELENOP macrophages identified 102 upregulated and 331 downregulated genes, among which SESN2, a stress-inducible gene implicated in oxidative stress control and redox homeostasis [39], was markedly reduced (Fig. 2A and B). Consistent with this finding, GO (Fig. S2C) and KEGG (Fig. 2C) analyses revealed significant enrichment of oxidative stress–related pathways. In parallel, immunofluorescence staining demonstrated increased MPO and Cit-H3 signals in sh-SELENOP macrophages, indicating enhanced extracellular trap formation (Fig. 2D). Reduced SESN2 expression after SELENOP silencing was further confirmed by qRT-PCR (Fig. 2E). We next investigated whether SESN2 mediates the effect of SELENOP on oxidative stress responses. Using an LPS/ATP-induced macrophage inflammatory model, we found that SELENOP knockdown significantly increased *Tnfα* and *Il1β* expression, whereas SESN2 reconstitution blunted this inflammatory response (Fig. 2F). Likewise, LDH release (Fig. 2G) and TBARS levels, reflecting MDA-related lipid peroxidation products (Fig. 2H) levels were increased, while SOD activity (Fig. 2I) was decreased in sh-SELENOP cells, and these alterations was partially restored by SESN2 overexpression. These data indicate that loss of SELENOP aggravates inflammatory and oxidative injury, at least in part, through suppression of SESN2.

Because SESN2 is closely linked to antioxidant signaling, we further examined the NRF2 pathway. SELENOP knockdown reduced nuclear NRF2 accumulation and decreased expression of the downstream antioxidant effectors HO-1 and NQO1, whereas SESN2 overexpression partially rescued these changes (Fig. 2J). In keeping with impaired antioxidant defense, SELENOP deficiency also increased ROS generation (Fig. 2K) and apoptosis (Fig. 2L), both of which were attenuated by SESN2 restoration. To substantiate these findings, we performed complementary gain-of-function experiments. SELENOP overexpression reduced LDH release and TBRAS levels while increasing SOD activity, and these protective effects were reversed by SESN2 knockdown (Fig. S2E–S2G). Similarly, SELENOP overexpression increased nuclear NRF2, HO-1, and NQO1 expression, whereas SESN2 silencing largely abrogated these effects (Fig. 2M). Moreover, the suppressive effects of SELENOP overexpression on ROS production and apoptosis were also lost upon SESN2 knockdown (Fig. 2N and O). Collectively, these results support a model in which SELENOP protects macrophages from LPS/ATP-induced inflammatory and oxidative injury through the SESN2/NRF2 axis.

### 3.3 SELENOP regulates oxidative stress and inflammasome through cyclase-associated protein 1 (CAP1)

**Fig. 3:**
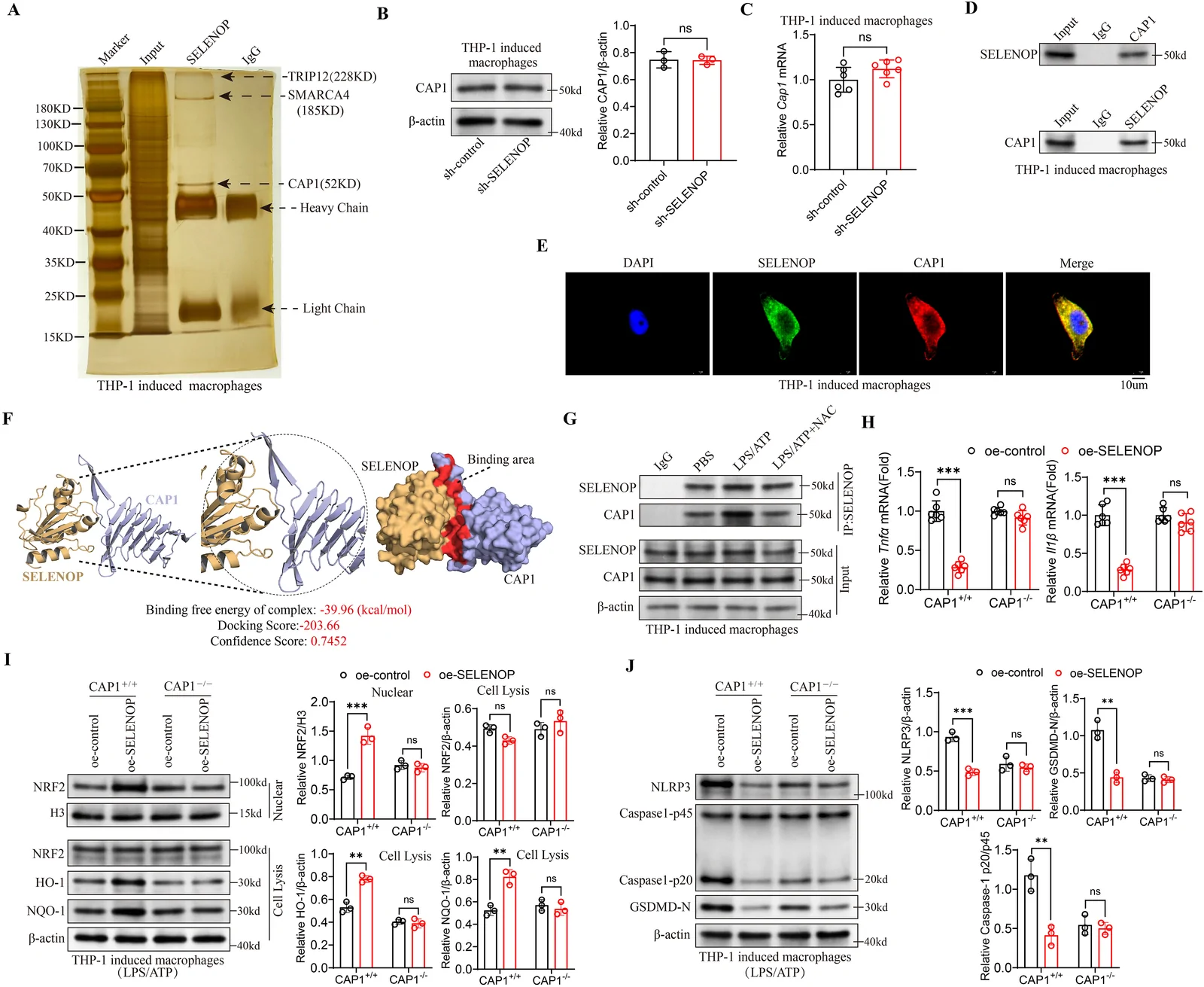
SELENOP regulates oxidative stress and inflammasome through CAP1 (A) Identification of SELENOP-interacting proteins in THP-1-derived macrophages by Co-IP, silver staining, and mass spectrometry. CAP1 was identified as a candidate SELENOP-binding protein. (B, C) CAP1 protein (B) and mRNA (C) expression in THP-1-derived macrophages transduced with sh-control or sh-SELENOP. (D) Reciprocal Co-IP analysis showing the endogenous interaction between SELENOP and CAP1 in THP-1-derived macrophages. (E) Representative immunofluorescence staining showing intracellular colocalization of SELENOP and CAP1 in THP-1-derived macrophages. Scale bar, 10 μm (F) Protein-protein docking model predicting the interaction between SELENOP and CAP1. (G) Co-IP analysis of the interaction between SELENOP and CAP1 in THP-1-derived macrophages treated with PBS, LPS/ATP, or LPS/ATP + NAC. (H) Relative *Tnfα* and *Il1β* mRNA expression in CAP1^+/+^ and CAP1^−/−^ macrophages transduced with oe-control or oe-SELENOP following LPS/ATP stimulation. (I) Representative immunoblot analysis and quantification of NRF2/HO-1/NQO-1 pathway in CAP1^+/+^ and CAP1^−/−^ macrophages transduced with oe-control or oe-SELENOP following LPS/ATP stimulation. Histone H3 and β-actin were used as loading controls for nuclear and total protein extracts, respectively. (J) Representative immunoblot analysis and quantification of NLRP3 inflammasome in CAP1^+/+^ and CAP1^−/−^ macrophages transduced with oe-control or oe-SELENOP following LPS/ATP stimulation. β-actin was used as a loading control. Co-IP, co-immunoprecipitation; DAPI, 4′,6-diamidino-2-phenylindole; CAP1, cyclase-associated protein 1; LPS, lipopolysaccharide; ATP, adenosine triphosphate; NAC, N-acetylcysteine; NRF2, nuclear factor erythroid 2-related factor 2; HO-1, heme oxygenase 1; NQO1, NAD(P)H quinone dehydrogenase 1; NLRP3, NLR family pyrin domain containing 3; GSDMD-N, N-terminal gasdermin D. Data are presented as mean ± SD. Statistical analysis was performed using Welch's two-tailed unpaired *t*-test. ns, not significant; *P < 0.05; **P < 0.01; ***P < 0.001.

To identify SELENOP-interacting proteins in macrophages, we performed Co-IP followed by silver staining and mass spectrometry. Among the candidate binding partners, CAP1, a molecule closely associated with oxidative stress by affecting ROS homeostasis, was identified (Fig. 3A). Because CAP1 has been implicated in stress-related signaling, we focused on this molecule for further study. SELENOP knockdown did not alter CAP1 protein or mRNA expression in THP-1–derived macrophages (Fig. 3B–C), indicating that SELENOP does not regulate CAP1 expression. Reciprocal Co-IP confirmed an endogenous interaction between SELENOP and CAP1 (Fig. 3D). This interaction was further supported by intracellular colocalization on immunofluorescence staining (Fig. 3E) and by protein-protein docking analysis (Fig. 3F). Notably, LPS/ATP stimulation enhanced the association between SELENOP and CAP1, whereas the ROS scavenger N-acetylcysteine (NAC) attenuated this interaction (Fig. 3G), suggesting that their binding is strengthened under oxidative stress.

We next assessed the functional relevance of CAP1 in SELENOP-mediated protection. In CAP1^+/+^ macrophages, SELENOP overexpression significantly reduced *Tnfα* and *Il1β* expression after LPS/ATP stimulation, whereas this effect was largely lost in CAP1^−/−^ cells (Fig. 3H). Similarly, SELENOP overexpression promoted NRF2 nuclear accumulation and increased HO-1 and NQO-1 expression in CAP1^+/+^ macrophages, but these effects were markedly blunted in CAP1^−/−^ cells, while total NRF2 levels remained unchanged (Fig. 3I). Given the well-established link between oxidative stress and NLRP3 inflammasome activation, we next examined whether the CAP1-dependent antioxidant effects of SELENOP were accompanied by changes in inflammasome signaling [40,41]. The inhibitory effects of SELENOP on NLRP3, GSDMD-N, and cleaved caspase-1 (p20) were also abolished by CAP1 deficiency (Fig. 3J). Together, these findings indicate that SELENOP interacts with CAP1 and exerts anti-inflammatory, antioxidant, and anti-pyroptotic effects in a CAP1-dependent manner, at least partly through NRF2 signaling.

### 3.4 TRIP12 promotes ubiquitin-dependent degradation of SELENOP

**Fig. 4:**
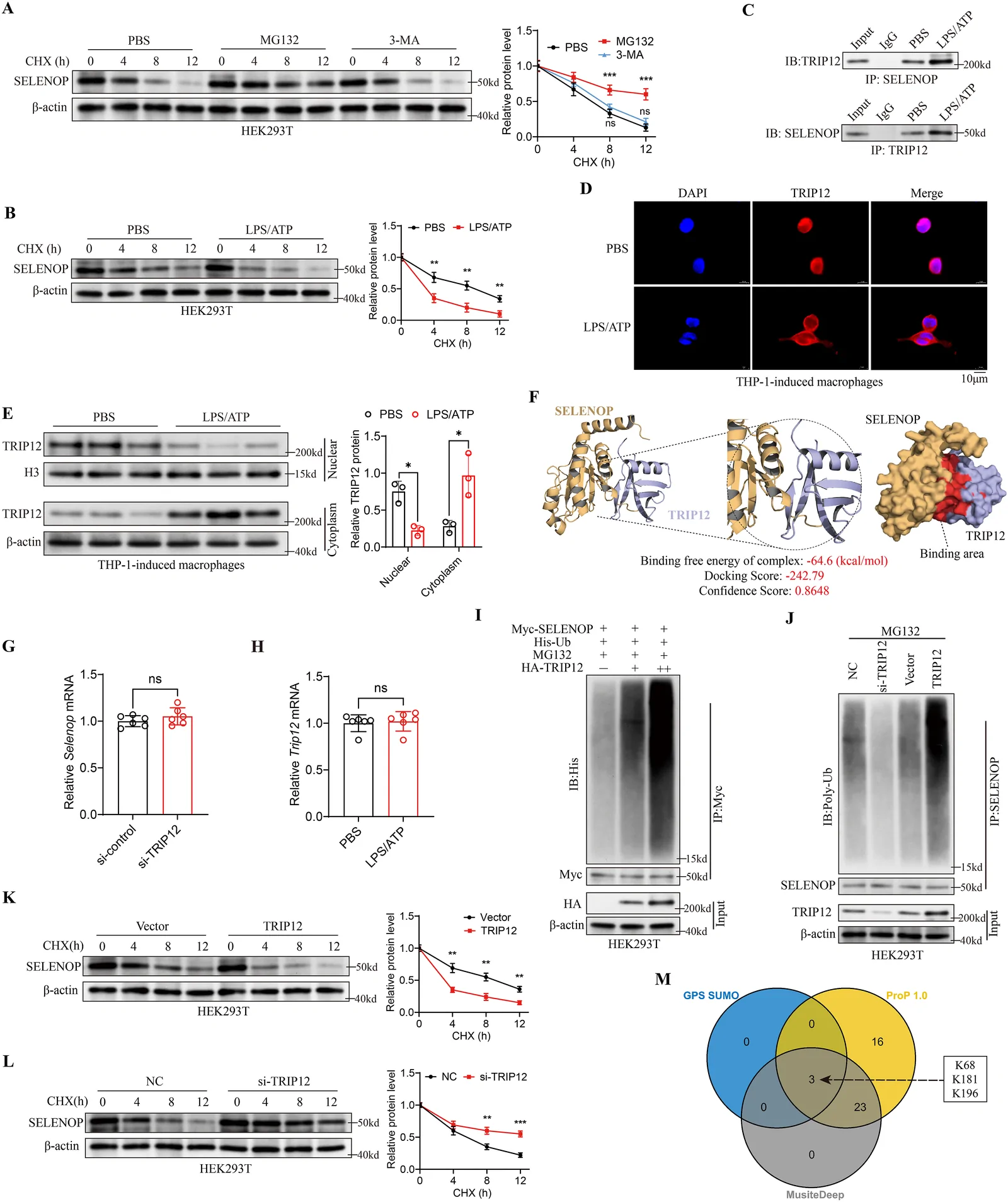
TRIP12 promotes ubiquitin-dependent proteasomal degradation of SELENOP. (A) Representative CHX chase assay and quantification of SELENOP protein levels in HEK293T cells treated with PBS, MG132, or 3-MA. (B) Representative CHX chase assay and quantification of SELENOP protein levels in HEK293T cells treated with PBS or LPS/ATP. (C) Endogenous Co-IP analysis showing the interaction between SELENOP and TRIP12 in cells treated with PBS or LPS/ATP. (D) Immunofluorescence analyses of TRIP12 in cells treated with PBS or LPS/ATP. (E) Nuclear/cytoplasmic fractionation followed by western blotting analyses of TRIP12 in cells treated with PBS or LPS/ATP. (F) Protein-protein docking model predicting the interaction between SELENOP and TRIP12. (G) Relative *Selenop* mRNA expression after TRIP12 knockdown. (H) Relative *Trip1*2 mRNA expression in cells treated with PBS or LPS/ATP. (I) Ubiquitination assay in HEK293T cells transfected with Myc-SELENOP, His-Ub, and HA-TRIP12, followed by immunoprecipitation with anti-Myc and immunoblotting with anti-His. (J) Endogenous ubiquitination assay showing polyubiquitination of SELENOP following TRIP12 knockdown or overexpression in the presence of MG132. (K) Representative CHX chase assay and quantification of SELENOP protein levels in HEK293T cells transfected with vector or TRIP12. (L) Representative CHX chase assay and quantification of SELENOP protein levels in HEK293T cells transfected with negative control siRNA (NC) or si-TRIP12. (M) Venn diagram showing the predicted ubiquitination sites of SELENOP identified by GPS-SUMO, ProP 1.0, and MusiteDeep. Three candidate lysine residues (K68, K181, and K196) were identified. CHX, cycloheximide; Co-IP, co-immunoprecipitation; MG132, carbobenzoxy-Leu-Leu-leucinal; 3-MA, 3-methyladenine; LPS, lipopolysaccharide; ATP, adenosine triphosphate; Ub, ubiquitin; poly-Ub, polyubiquitin; PBS, phosphate-buffered saline; IB, immunoblot; NC, negative control. Data are presented as mean ± SD. Statistical analysis was performed using Welch's two-tailed unpaired *t*-test. ns, not significant; *P < 0.05; **P < 0.01; ***P < 0.001.

**Fig. S3:**
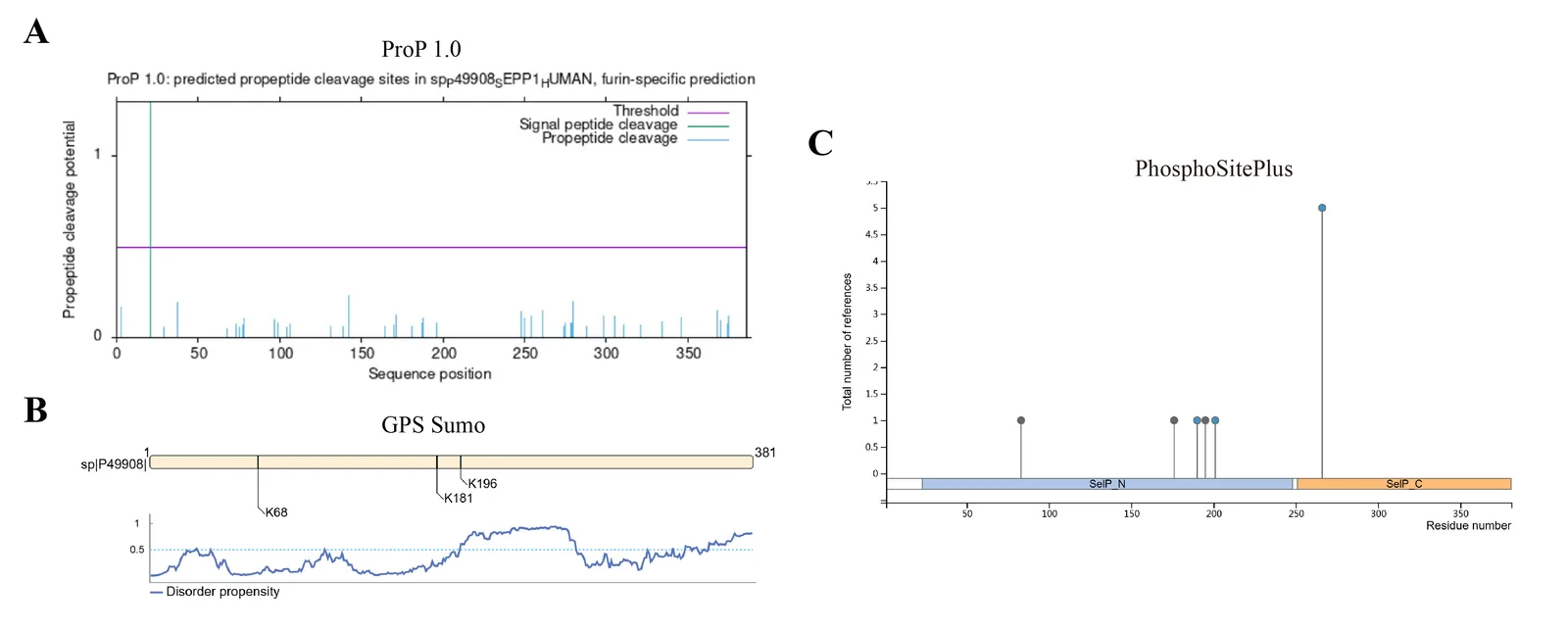
Structural and post-translational modification analysis of SELENOP. (A) Predicted protein–protein docking model of SELENOP in complex with TRIP12, with a zoomed-in view of the interaction interface showing key residues and distances. (B) Prediction of signal peptide and propeptide cleavage sites in SELENOP using ProP 1.0. (C) Prediction of SUMOylation sites and intrinsic disorder propensity of SELENOP using GPS-SUMO. (D) Distribution of experimentally validated phosphorylation sites in SELENOP based on the PhosphoSitePlus database.

To investigate how SELENOP is regulated at the post-translational level, we first examined its protein stability using cycloheximide (CHX) chase assays. Pharmacological inhibition of the proteasome with MG132 markedly delayed SELENOP degradation, whereas the autophagy inhibitor 3-MA had little effect, indicating that SELENOP is primarily degraded through the ubiquitin-proteasome pathway rather than autophagy (Fig. 4A). Notably, LPS/ATP stimulation accelerated SELENOP protein turnover (Fig. 4B), suggesting that inflammatory stress promotes SELENOP degradation. Since our Co-IP/silver staining and mass spectrometry analysis (Fig. 3A) had identified TRIP12 as a highly ranked SELENOP-interacting E3 ligase candidate, we next examined the interaction between SELENOP and TRIP12. Endogenous Co-IP demonstrated that SELENOP interacted with TRIP12, and this association was enhanced following LPS/ATP stimulation (Fig. 4C). TRIP12 is primarily localized in the nucleus under resting conditions, whereas SELENOP is not exclusively localized in the nucleus. The immunofluorescence analyses (Fig. 4D) and nuclear/cytoplasmic fractionation followed by western blotting (Fig. 4E) demonstrated that, under inflammatory stimulation with LPS/ATP, TRIP12 exhibits increased nuclear export and accumulates in the cytoplasm, resulting in enhanced spatial proximity and interaction with SELENOP. Protein-protein docking analysis further supported a direct binding mode between SELENOP and TRIP12 with favorable binding energy (Fig. 4F, Fig. S3A). Importantly, TRIP12 knockdown did not affect *Selenop* mRNA levels (Fig. 4G), and LPS/ATP stimulation did not alter *Trip1*2 mRNA expression (Fig. 4H), arguing against transcriptional regulation and supporting a post-translational mechanism.

In HEK293T cells, TRIP12 overexpression markedly increased SELENOP ubiquitination in the presence of ubiquitin and MG132 (Fig. 4I). Consistently, endogenous ubiquitination assays showed that TRIP12 silencing reduced, whereas TRIP12 overexpression increased the ubiquitin level of SELENOP (Fig. 4J). Functionally, CHX chase experiments demonstrated that TRIP12 overexpression accelerated SELENOP degradation, while TRIP12 knockdown stabilized SELENOP protein (Fig. 4K–L). Finally, in silico prediction identified three potential ubiquitination sites in SELENOP, namely K68, K181, and K196 (Fig. 4M, Fig. S3B–D). Together, these data indicate that TRIP12 interacts with SELENOP and promotes its ubiquitin-dependent proteasomal degradation, a process that is enhanced under inflammatory stimulation.

### 3.5 CREB1 cooperates with SMARCA4 to transcriptionally activate SELENOP expression

**Fig. 5:**
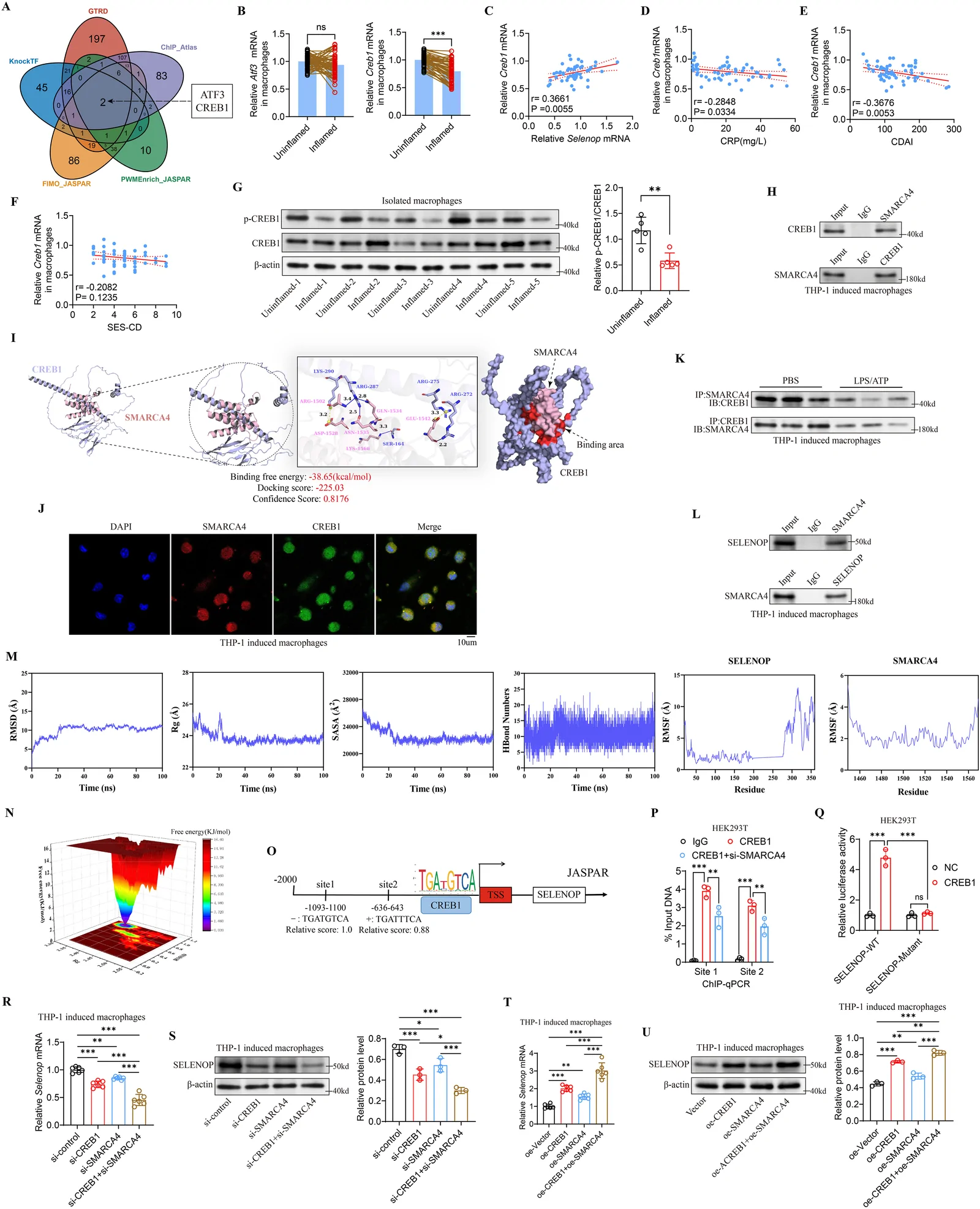
CREB1 cooperates with SMARCA4 to transcriptionally activate SELENOP expression. (A) Venn diagram of candidate upstream transcription factors predicted by multiple transcription factor databases, identifying CREB1 and ATF3 as shared candidates for SELENOP regulation. (B) Relative *Atf3* and *Creb1* mRNA expression in macrophages isolated from uninflamed and inflamed colonic tissues. (C–F) Correlation analyses between macrophage *Creb1* mRNA expression and *Selenop* mRNA expression (C), CRP (D), CDAI (E), and SES-CD (F). (G) Representative immunoblot analysis and quantification of p-CREB1 and total CREB1 protein expression in macrophages isolated from uninflamed and inflamed tissues. (H) Reciprocal Co-IP analysis showing the endogenous interaction between CREB1 and SMARCA4 in THP-1-derived macrophages. (I) Protein-protein docking model predicting the interaction between CREB1 and SMARCA4. (J) Reciprocal Co-IP analysis showing the endogenous interaction between SELENOP and SMARCA4 in THP-1-derived macrophages. (K) Representative immunofluorescence staining showing intracellular colocalization of SMARCA4 and CREB1 in THP-1-derived macrophages. Scale bar, 10 μm (L) Co-IP analysis of the interaction between CREB1 and SMARCA4 in THP-1-derived macrophages treated with PBS or LPS/ATP. (M) Molecular dynamics simulation of the SELENOP-SMARCA4 complex, showing RMSD, Rg, SASA, hydrogen bond number, and RMSF profiles over time. (N) Free-energy landscape analysis of the SELENOP-SMARCA4 interaction based on molecular dynamics simulation. (O) Schematic representation of the predicted CREB1-binding sites within the SELENOP promoter based on JASPAR database. (P) ChIP-qPCR analysis showing CREB1 enrichment at the indicated SELENOP promoter regions in HEK293T cells transfected with control, CREB1, or CREB1+ si-SMARCA4. (Q) Dual-luciferase reporter assay showing the effect of CREB1 overexpression on wild-type or mutant SELENOP promoter activity in HEK293T cells. (R, S) Relative *Selenop* mRNA (R) and protein (S) expressions in THP-1-derived macrophages transfected with si-control, si-CREB1, si-SMARCA4, or si-CREB1+si-SMARCA4. (T, U) Relative *Selenop* mRNA (T) and protein (U) expressions in THP-1-derived macrophages transfected with vector, oe-CREB1, oe-SMARCA4, or oe-CREB1+oe-SMARCA4. CRP, C-reactive protein; CDAI, Crohn's disease activity index; SES-CD, Simple Endoscopic Score for Crohn's Disease; Co-IP, co-immunoprecipitation; DAPI, 4′,6-diamidino-2-phenylindole; PBS, phosphate-buffered saline; LPS, lipopolysaccharide; ATP, adenosine triphosphate; ChIP-qPCR, chromatin immunoprecipitation followed by quantitative PCR; RMSD, root mean square deviation; Rg, radius of gyration; SASA, solvent-accessible surface area; RMSF, root mean square fluctuation. Data are presented as mean ± SD. Statistical analysis was performed using paired Student's *t*-test (B, C), Welch's two-tailed unpaired *t*-test (G, Q), or one-way ANOVA followed by Tukey's post hoc test (P, R, S, T, and U). ns, not significant; *P < 0.05; **P < 0.01; ***P < 0.001.

**Fig. S4:**
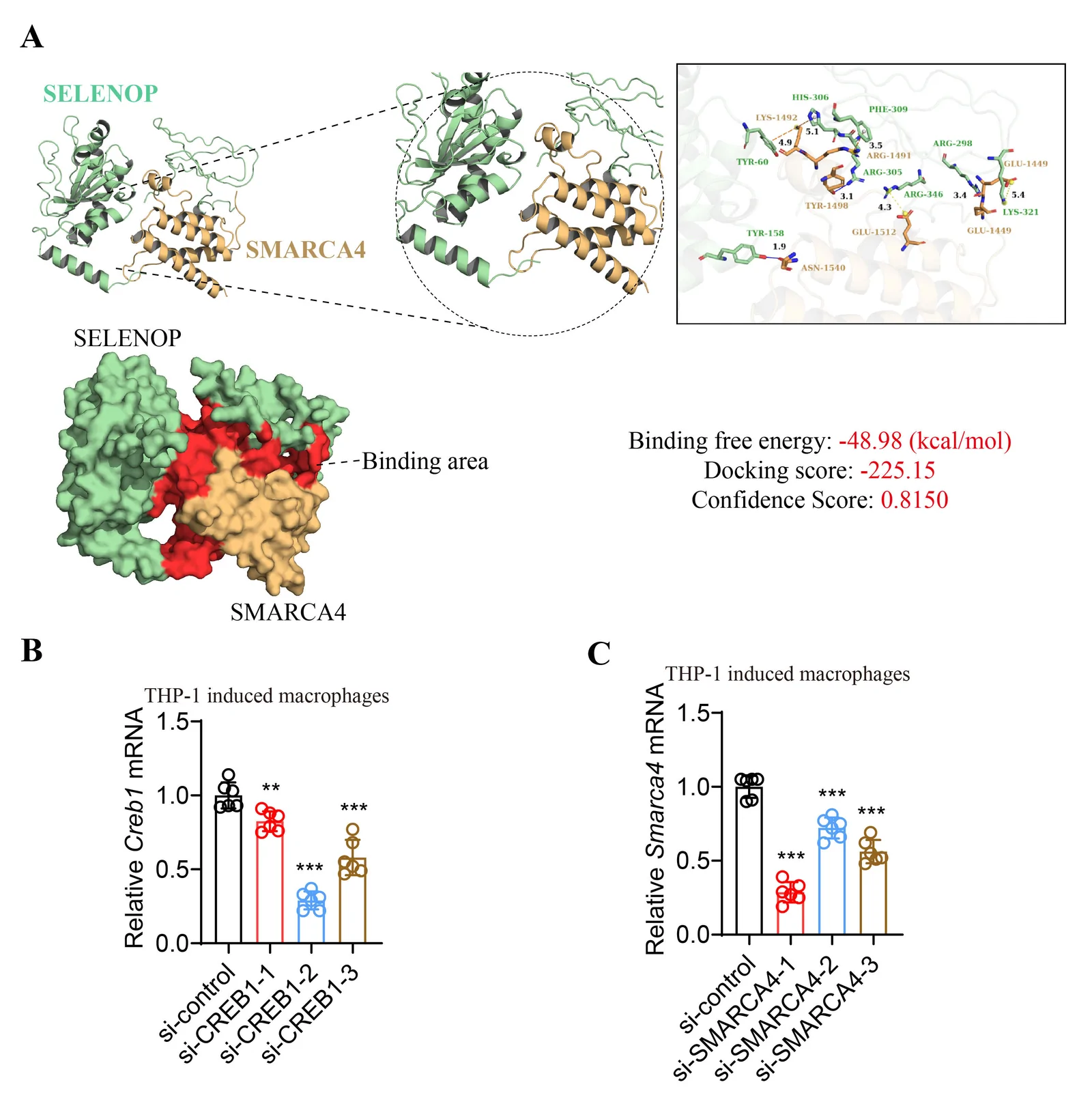
Structural interaction and knockdown validation of SMARCA4/CREB1. (A) Predicted protein–protein docking model of SELENOP in complex with SMARCA4, showing the binding interface and interaction details. (B-C) qRT-PCR validation of CREB1 (B) and SMARCA4 (C) knockdown efficiency in THP-1–derived macrophages. Data are presented as mean ± SD. Statistical analysis was performed using one-way ANOVA followed by Tukey's post hoc test. *P < 0.05; **P < 0.01; ***P < 0.001.

SMARCA4, a known transcriptional co-regulator, was also identified as a top-ranked SELENOP-interacting protein in our previous Co-IP/mass spectrometry analysis (Fig. 3A), we therefore examined the transcriptional regulation of SELENOP. The multiple transcription factor prediction databases identified CREB1 and ATF3 as candidate upstream regulators (Fig. 5A). In macrophages from inflamed tissues, *Atf3* mRNA was unchanged, whereas *Creb1* expression was markedly reduced (Fig. 5B). Consistently, *Creb1* expression positively correlated with *Selenop* mRNA levels (Fig. 5C), and negatively correlated with CRP (Fig. 5D) and CDAI (Fig. 5E), with no significant correlation with SES-CD (Fig. 5F). Although total CREB1 protein remained unchanged, phosphorylated CREB1 was significantly decreased in inflamed macrophages, indicating impaired CREB1 activation under inflammatory conditions (Fig. 5G). Reciprocal Co-IP confirmed an endogenous interaction between CREB1 and SMARCA4 (Fig. 5H), which was further supported by protein docking (Fig. 5I) and intracellular colocalization (Fig. 5J). Notably, LPS/ATP stimulation weakened the CREB1-SMARCA4 interaction (Fig. 5K). In addition, reciprocal Co-IP confirmed an endogenous interaction between SELENOP and SMARCA4 (Fig. 5L), which was supported by protein docking (Fig. S4A) and molecular dynamics simulation (Fig. 5M and N).

Given the interaction between SMARCA4/CREB1 and SELENOP (Fig. 5J), we further assessed whether this complex regulates SELENOP transcription. Bioinformatic analysis identified two putative CREB1-binding sites in the SELENOP promoter (Fig. 5O). ChIP-qPCR confirmed CREB1 enrichment at both sites, which was markedly reduced after SMARCA4 silencing (Fig. 5P). Consistently, luciferase assays showed that CREB1 overexpression increased wild-type SELENOP promoter activity, whereas mutation of the predicted binding motif largely abolished this effect (Fig. 5Q). Functionally, silencing of either CREB1 or SMARCA4 (silencing efficiency see Fig. S4B and S4C) reduced SELENOP mRNA (Fig. 5R) and protein (Fig. 5S) expressions, with a stronger effect upon combined knockdown, whereas overexpression of either factor increased SELENOP expression, and co-overexpression produced the greatest induction (Fig. 5T and U). Together, these data indicate that CREB1 transcriptionally activates SELENOP, while SMARCA4 acts as a cooperative co-regulator in this process.

### 3.6 Macrophage SELENOP deficiency promotes neutrophil inflammatory and oxidative injury in a co-culture model

**Fig. 6:**
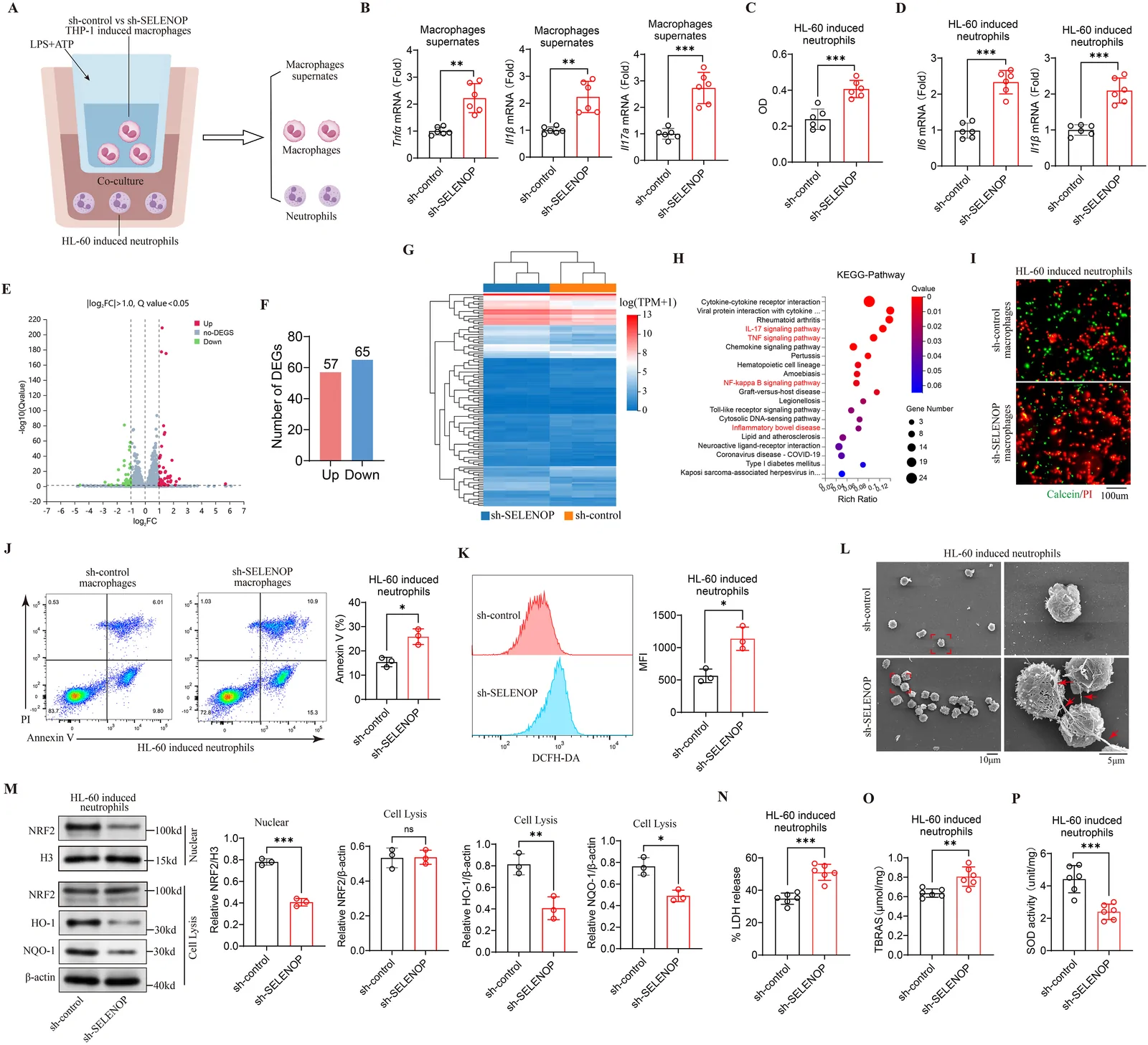
Macrophage SELENOP deficiency promotes neutrophil inflammatory and oxidative injury in a co-culture model. (A) Schematic illustration of the transwell co-culture system in which THP-1-derived macrophages transduced with sh-control or sh-SELENOP under LPS/ATP stimulation were co-cultured with HL-60-induced neutrophils. (B) Relative *Tnfα, Il1β*, and *Il17A* mRNA expression in sh-control and sh-SELENOP macrophages after co-culture. (C) OD values of HL-60-induced neutrophils co-cultured with sh-control or sh-SELENOP macrophages. (D) Relative *Il6* and *Il1β* mRNA expression in HL-60-induced neutrophils co-cultured with sh-control or sh-SELENOP macrophages. (E-G) Differentially expressed genes in HL-60-induced neutrophils co-cultured with sh-control or sh-SELENOP macrophages. (H) KEGG pathway enrichment analysis of differentially expressed genes in HL-60-induced neutrophils, highlighting inflammatory signalling pathways. (I) Representative Calcein/PI staining of HL-60-induced neutrophils co-cultured with sh-control or sh-SELENOP macrophages. Scale bar, 100 μm (J) Representative Annexin V/PI flow cytometric analysis of HL-60-induced neutrophils after co-culture with sh-control or sh-SELENOP macrophages. (K) Representative DCFH-DA flow cytometric analysis and quantification of intracellular ROS levels in HL-60-induced neutrophils after co-culture with sh-control or sh-SELENOP macrophages. (L) Representative neutrophils extracellular trap-like structures by scanning electron microscopy images of HL-60-induced neutrophils after co-culture with sh-control or sh-SELENOP macrophages. Scale bars, 10 μm and 5 μm (M) Representative immunoblot analysis and quantification of NRF2/HO-1/NQO-1 pathway in HL-60-induced neutrophils after co-culture with sh-control or sh-SELENOP macrophages. H3 or β-actin were used as loading controls. (N–P) LDH release (N), TBARS levels (O), and SOD activity (P) in HL-60-induced neutrophils after co-culture with sh-control or sh-SELENOP macrophages. OD, optical density; KEGG, Kyoto Encyclopedia of Genes and Genomes; PI, propidium iodide; ROS, reactive oxygen species; DCFH-DA, 2′,7′-dichlorodihydrofluorescein diacetate; NRF2, nuclear factor erythroid 2-related factor 2; HO-1, heme oxygenase 1; NQO-1, NAD(P)H quinone dehydrogenase 1; LDH, lactate dehydrogenase; TBARS, thiobarbituric acid reactive substances; SOD, superoxide dismutase; LPS, lipopolysaccharide; ATP, adenosine triphosphate. Data are presented as mean ± SD. Statistical analysis was performed using Welch's two-tailed unpaired *t*-test. ns, not significant; *P < 0.05; **P < 0.01; ***P < 0.001.

**Fig. S5:**
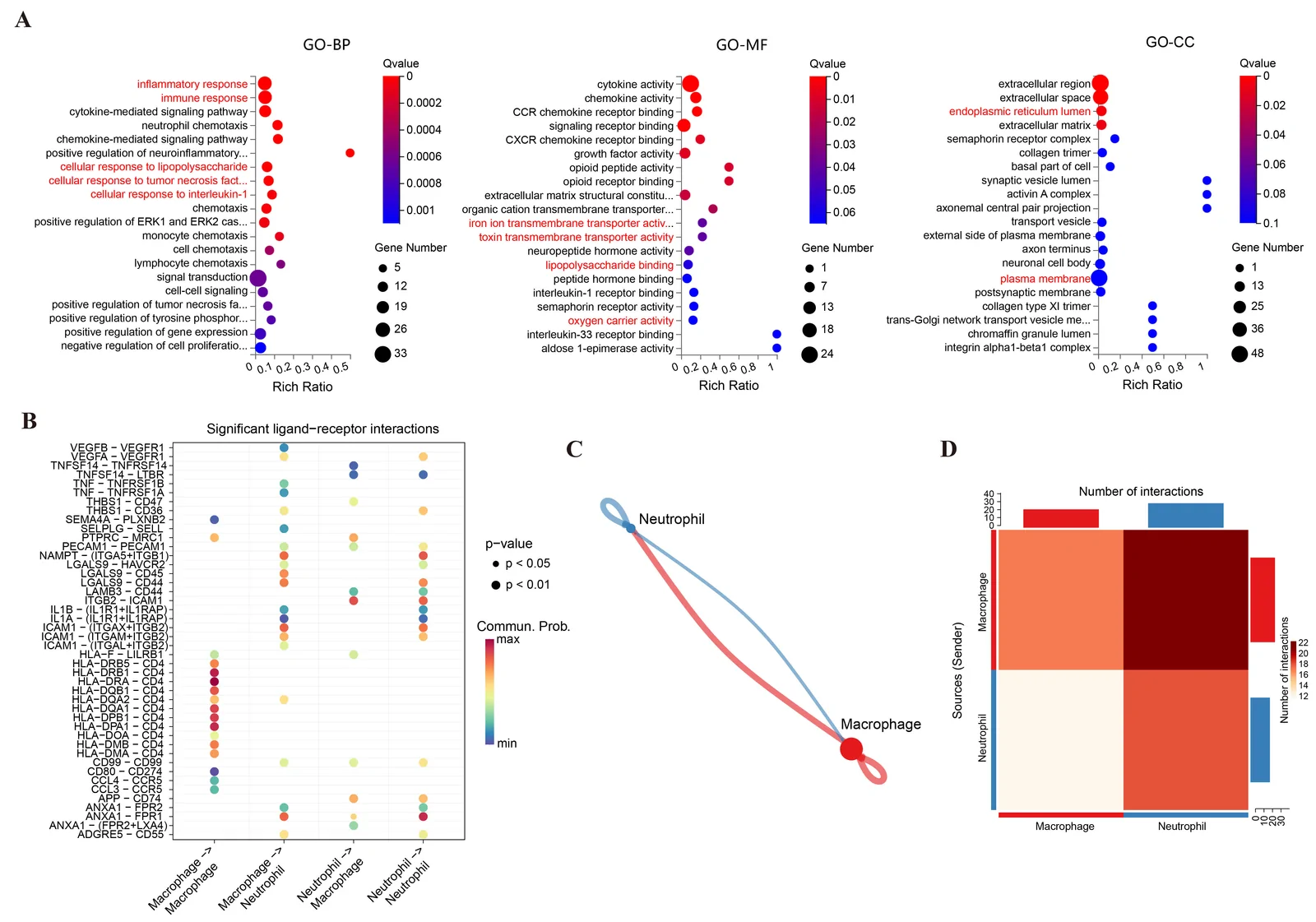
Cell–cell communication analysis between macrophages and neutrophils based on scRNA-seq. (A) GO enrichment analysis of neutrophil differentially expressed genes, showing enriched terms in BP, MF, and CC categories. (B-D) Cell–cell communication analysis depicting ligand–receptor interactions (B), network connectivity (C), and interaction strength (D) between macrophages and neutrophils. GO, Gene Ontology; BP, biological process; MF, molecular function; CC, cellular component; scRNA-seq, single-cell RNA sequencing.

To determine whether SELENOP-deficient macrophages affect neutrophil responses, we established a transwell co-culture system using THP-1–derived macrophages and HL-60–induced neutrophils under LPS/ATP stimulation (Fig. 6A). sh-SELENOP macrophages exhibited increased *Tnfα, Il1β,* and *Il17a* expression, indicating an enhanced proinflammatory phenotype (Fig. 6B). Accordingly, cocultured neutrophils showed increased inflammatory activation, as evidenced by elevated OD (Fig. 6C) values and increased *Il6* and *Il1β* expression (Fig. 6D). Transcriptomic analysis further identified 57 upregulated and 65 downregulated genes in neutrophils exposed to sh-SELENOP macrophages (Fig. 6E–F), with clear clustering between groups (Fig. 6G). GO (Fig. S5A) and KEGG (Fig. 6H) analysis revealed enrichment of inflammatory pathways, including IL-17, TNF, and NF-κB signaling pathway. Functionally, neutrophils cocultured with sh-SELENOP macrophages displayed increased cell death (Fig. 6I) and apoptosis (Fig. 6J), accompanied by elevated ROS production (Fig. 6K), and enhanced extracellular trap-like structures (Fig. 6L). Mechanistically, nuclear NRF2, HO-1, and NQO-1 levels were reduced, whereas total NRF2 remained unchanged (Fig. 6M). Consistent with impaired antioxidant defense, LDH release and TBARS levels were increased and SOD activity was decreased in neutrophils exposed to sh-SELENOP macrophages (Fig. 6N–P). In addition, CellChat analysis of macrophages-neutrophils ligand–receptor pathway interactions based on scRNA-seq was presented in Fig. S5B–D. Together, these data indicate that macrophage SELENOP deficiency drives neutrophil inflammatory and oxidative injury, at least partly through suppression of NRF2-dependent antioxidant signaling.

### 3.7 Macrophage-derived SELENOP protects neutrophils from inflammatory and oxidative injury through a cADPR-sensitive NRF2 pathway

**Fig. 7:**
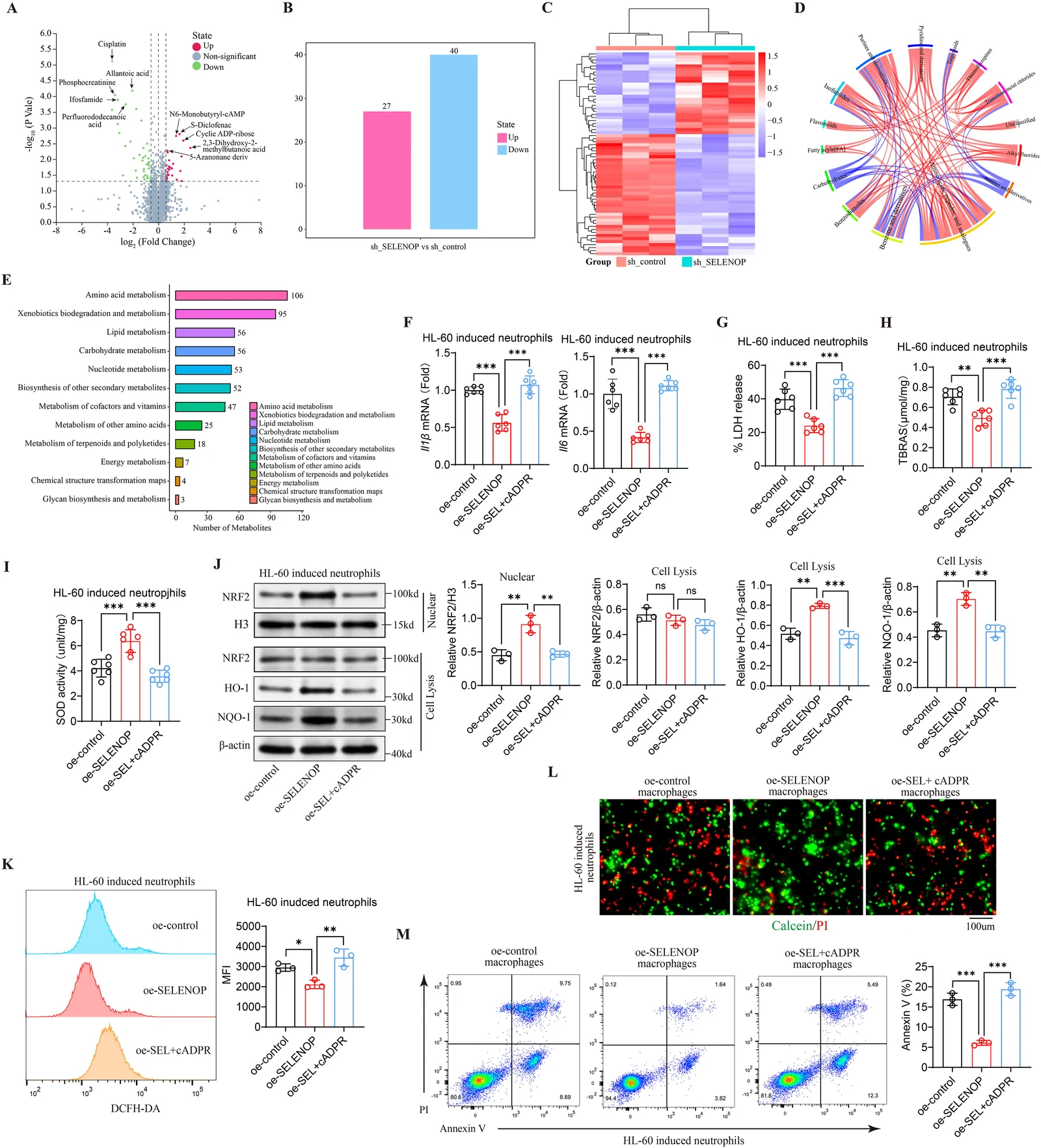
Macrophage-derived SELENOP protects neutrophils from inflammatory and oxidative injury through a cADPR-sensitive NRF2 pathway. (A) Volcano plot showing differentially abundant metabolites in supernatants from LPS/ATP-stimulated sh-control and sh-SELENOP macrophages. Selected metabolites are labelled. (B-C) Increased and decreased metabolites in supernatants from sh-control and sh-SELENOP macrophages. (D) Chord diagram showing the relationships between differentially abundant metabolites and enriched metabolic pathways. (E) Pathway enrichment analysis of differentially abundant metabolites. (F) Relative *Il1β* and *Il6* mRNA expression in HL-60-induced neutrophils cultured with supernatants from oe-control or oe-SELENOP macrophages in the presence or absence of cADPR. (G–I) LDH release (G), TBRAS levels (H), and SOD activity (I) in HL-60-induced neutrophils cultured with supernatants from oe-control or oe-SELENOP macrophages in the presence or absence of cADPR. (J) Representative immunoblot analysis and quantification of NRF2/HO-1/NQO-1 pathway in HL-60-induced neutrophils cultured with oe-control or oe-SELENOP macrophages in the presence or absence of cADPR. H3 or β-actin was used as loading controls. (K-M) Representative DCFH-DA flow cytometric analysis of ROS levels (K), Calcein/PI staining (L), and Annexin V/PI flow cytometric analysis (M) in HL-60-induced neutrophils cultured with oe-control or oe-SELENOP macrophages in the presence or absence of cADPR. cADPR, cyclic ADP-ribose; LPS, lipopolysaccharide; ATP, adenosine triphosphate; LDH, lactate dehydrogenase; TBARS, thiobarbituric acid reactive substances; SOD, superoxide dismutase; NRF2, nuclear factor erythroid 2-related factor 2; HO-1, heme oxygenase 1; NQO-1, NAD(P)H quinone dehydrogenase 1; ROS, reactive oxygen species; DCFH-DA, 2′,7′-dichlorodihydrofluorescein diacetate; PI, propidium iodide. Data are presented as mean ± SD. Statistical analysis was performed using one-way ANOVA followed by Tukey's post hoc test (F-M). ns, not significant; *P < 0.05; **P < 0.01; ***P < 0.001.

**Fig. S6:**
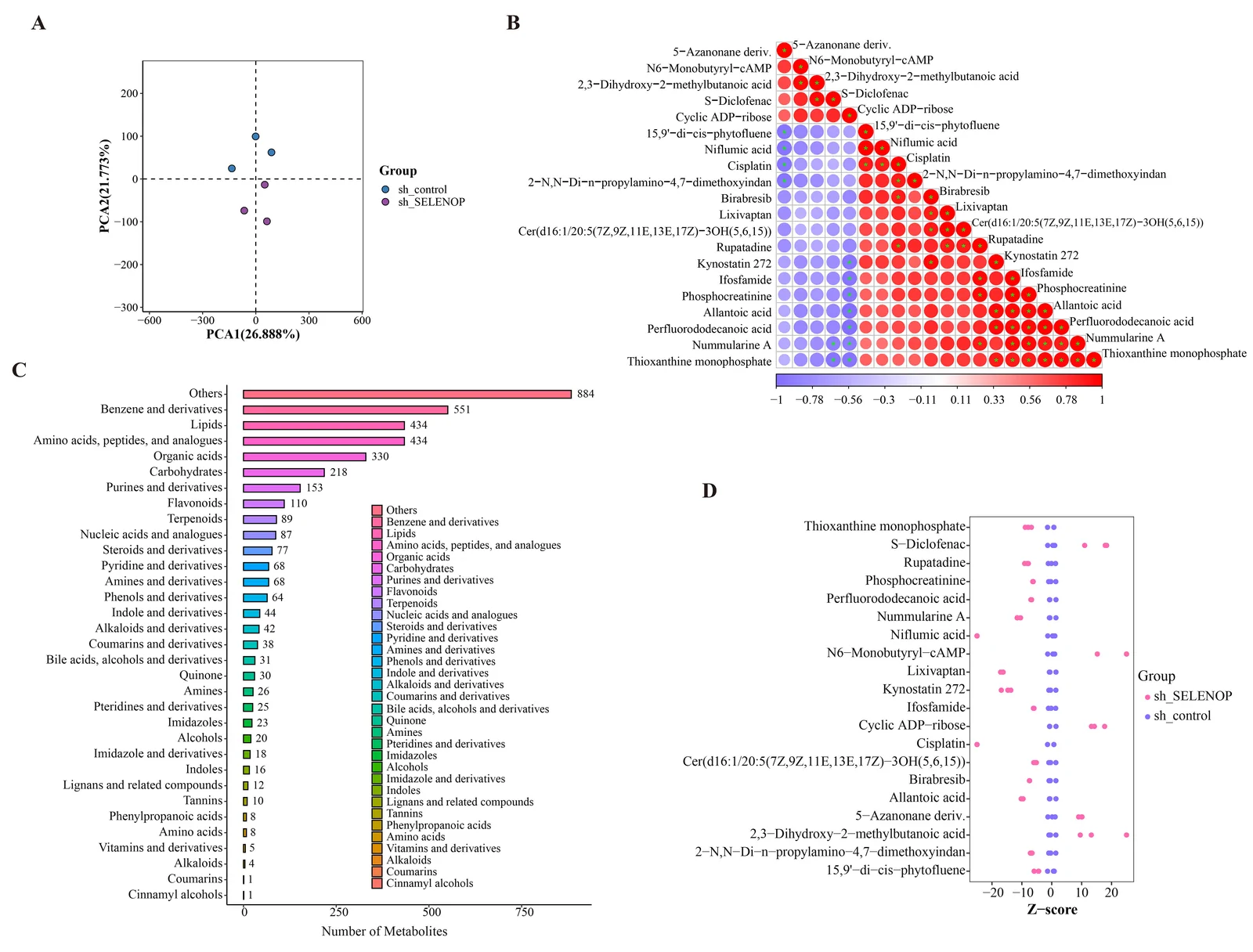
Metabolomic profiling of macrophage supernatants following SELENOP knockdown. (A) Principal component analysis (PCA) showing metabolic profile separation between sh-control and sh-SELENOP groups. (B) Correlation matrix of differentially abundant metabolites. (C) Classification of identified metabolites based on chemical categories. (D) Z-score plot showing relative abundance of representative differential metabolites between groups.

To further define how SELENOP-deficient macrophages affect neutrophil responses, we collected supernate from LPS/ATP induced sh-control and sh-SELENOP macrophages for metabolomic analysis. Compared with the sh-control group, sh-SELENOP supernate exhibited a distinct metabolic profile, with 27 metabolites increased and 40 metabolites decreased (Fig. 7A–B). Hierarchical clustering further showed clear separation between groups (Fig. 7C), and pathway enrichment analysis indicated that these differentially abundant metabolites were mainly involved in amino acid, lipid, carbohydrate, and nucleotide metabolism, as well as xenobiotics biodegradation and metabolism (Fig. 7D–E, Fig. S6A–D). Among the dysregulated metabolites, cADPR is closely associated with oxidative stress, as it links CD38-dependent Ca^2+^ mobilization to ROS amplification and oxidative injury [42]. To validate the functional relevance of these findings, neutrophils were then cocultured with oe-control or oe-SELENOP macrophages supernate in the presence or absence of cADPR. Macrophage SELENOP overexpression reduced *Il1β* and *Il6* expression in neutrophils, whereas cADPR largely abolished these effects (Fig. 7F). Likewise, LDH release (Fig. 7G) and TBARS (Fig. 7H) levels were decreased, while SOD activity (Fig. 7I) was increased, in neutrophils cocultured with oe-SELENOP macrophages; these protective effects were reversed by cADPR. Consistently, oe-SELENOP promoted nuclear NRF2 accumulation and increased HO-1 and NQO-1 expression in neutrophils without affecting total NRF2 levels, and these changes were attenuated by cADPR (Fig. 7J). In parallel, ROS production (Fig. 7K), cell death (Fig. 7L), and apoptosis (Fig. 7M) were all reduced in neutrophils exposed to oe-SELENOP macrophages, whereas cADPR largely abrogated these effects. Collectively, these findings indicate that macrophage-derived SELENOP protects neutrophils against inflammatory and oxidative injury, at least partly through metabolite cADPR.

### 3.8 Myeloid-derived SELENOP protects against experimental colitis by preserving epithelial barrier integrity and redox homeostasis

**Fig. 8:**
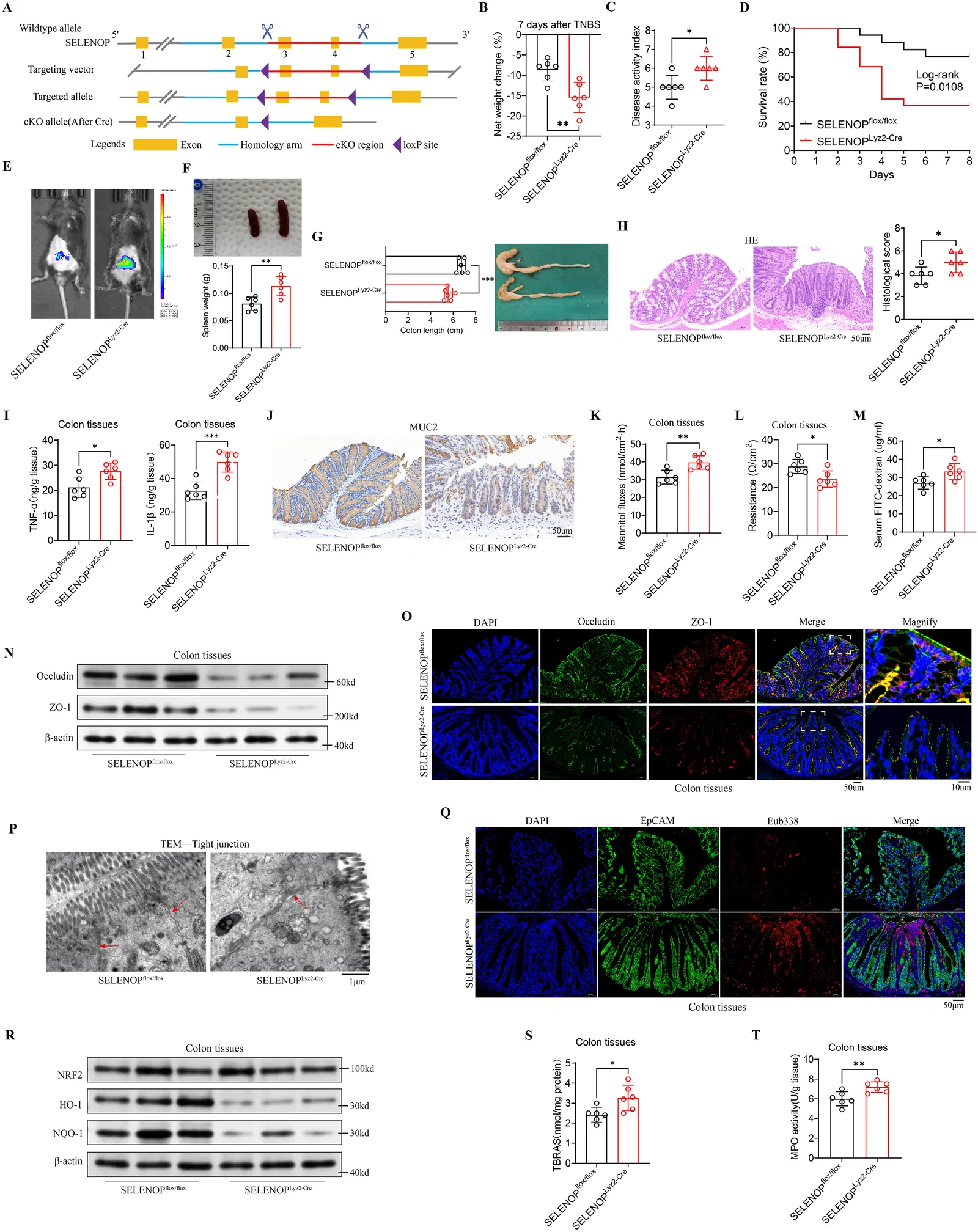
Myeloid-derived SELENOP protects against experimental colitis by preserving epithelial barrier integrity and redox homeostasis. (A) Strategy for generation of Lyz2-Cre-mediated myeloid-specific SELENOP conditional knockout mice (SELENOP^Lyz2−Cre^). (B–D) Body weight change (B), DAI (C), and survival (D) in SELENOPflox/flox and SELENOPLyz2-Cre mice following TNBS administration. (E) Representative in vivo imaging showing neutrephil infiltration in SELENOPflox/flox and SELENOPLyz2-Cre mice after TNBS treatment. (F, G) Spleen size and spleen weight (F), and colon length (G) in SELENOPflox/flox and SELENOPLyz2-Cre mice after TNBS treatment. (H) Representative H&E staining of colonic tissues and histological scores in SELENOPflox/flox and SELENOPLyz2-Cre mice after TNBS treatment. Scale bar, 50 μm. (I) TNF-α and IL-1β levels in colonic tissues from SELENOPflox/flox and SELENOPLyz2-Cre mice after TNBS treatment by ELISA. (J) Representative immunohistochemical staining of MUC2 in colonic tissues from SELENOPflox/flox and SELENOPLyz2-Cre mice. Scale bars, 50 μm. (K–M) Mannitol fluxes (K), transepithelial resistance (L), and serum FITC-dextran levels (M) in SELENOPflox/flox and SELENOPLyz2-Cre mice after TNBS treatment. (N-O) Representative western blotting (N) and immunofluorescence staining (O) of Occludin and ZO-1 protein expression in colonic tissues from SELENOPflox/flox and SELENOPLyz2-Cre mice. Scale bars, 50 μm and 10 μm (P) Representative transmission electron microscopy images showing tight junction ultrastructure in colonic tissues from SELENOPflox/flox and SELENOPLyz2-Cre mice. Arrows indicate tight junctions. Scale bar, 1 μm. (Q) Representative immunofluorescence staining of colonic tissues for EpCAM (green) and Eub338 (red). Scale bar, 50 μm (R) Representative western blotting of NRF2/HO-1/NQO-1 pathway in colonic tissues. (S–T) TBRAS levels (S), and MPO activity (T) in colonic tissues from SELENOP^flox/flox^ and SELENOP^Lyz2−Cre^ mice after TNBS treatment. TNBS, 2,4,6-trinitrobenzene sulfonic acid; DAI, disease activity index; H&E, haematoxylin and eosin; TNF-α, tumour necrosis factor α; IL-1β, interleukin 1β; MUC2, mucin 2; ELISA, enzyme-linked immunosorbent assay; FITC, fluorescein isothiocyanate; ZO-1, zonula occludens-1; DAPI, 4′,6-diamidino-2-phenylindole; EpCAM, epithelial cell adhesion molecule; NRF2, nuclear factor erythroid 2-related factor 2; HO-1, heme oxygenase 1; NQO-1, NAD(P)H quinone dehydrogenase 1; TBARS, thiobarbituric acid reactive substances; MPO, myeloperoxidase. Data are presented as mean ± SD. Statistical analysis was performed using Welch's two-tailed unpaired *t*-test. *P < 0.05; **P < 0.01; ***P < 0.001.

To investigate the role of myeloid-derived SELENOP in colitis, we generated Lyz2-Cre–mediated Selenop conditional knockout mice (SELENOP^Lyz2-Cre) and subjected them to TNBS-induced colitis (Fig. 8A). Compared with littermate controls, SELENOP^Lyz2-Cre mice developed more severe disease, as indicated by greater body weight loss, higher disease activity index (DAI), and reduced survival following TNBS challenge (Fig. 8B–D). In vivo imaging further revealed stronger abdominal inflammatory signals in knockout mice (Fig. 8E). Consistently, myeloid SELENOP deficiency was associated with splenomegaly and colon shortening, indicating aggravated systemic and intestinal inflammation (Fig. 8F and G). Histological examination showed more severe mucosal injury, inflammatory infiltration, and crypt damage in SELENOP^Lyz2-Cre mice, together with significantly increased histological scores (Fig. 8H). In parallel, colonic TNF-α and IL-1β levels were elevated in knockout mice (Fig. 8I), supporting a protective anti-inflammatory role for myeloid-derived SELENOP during acute colitis.

**Fig. S7:**
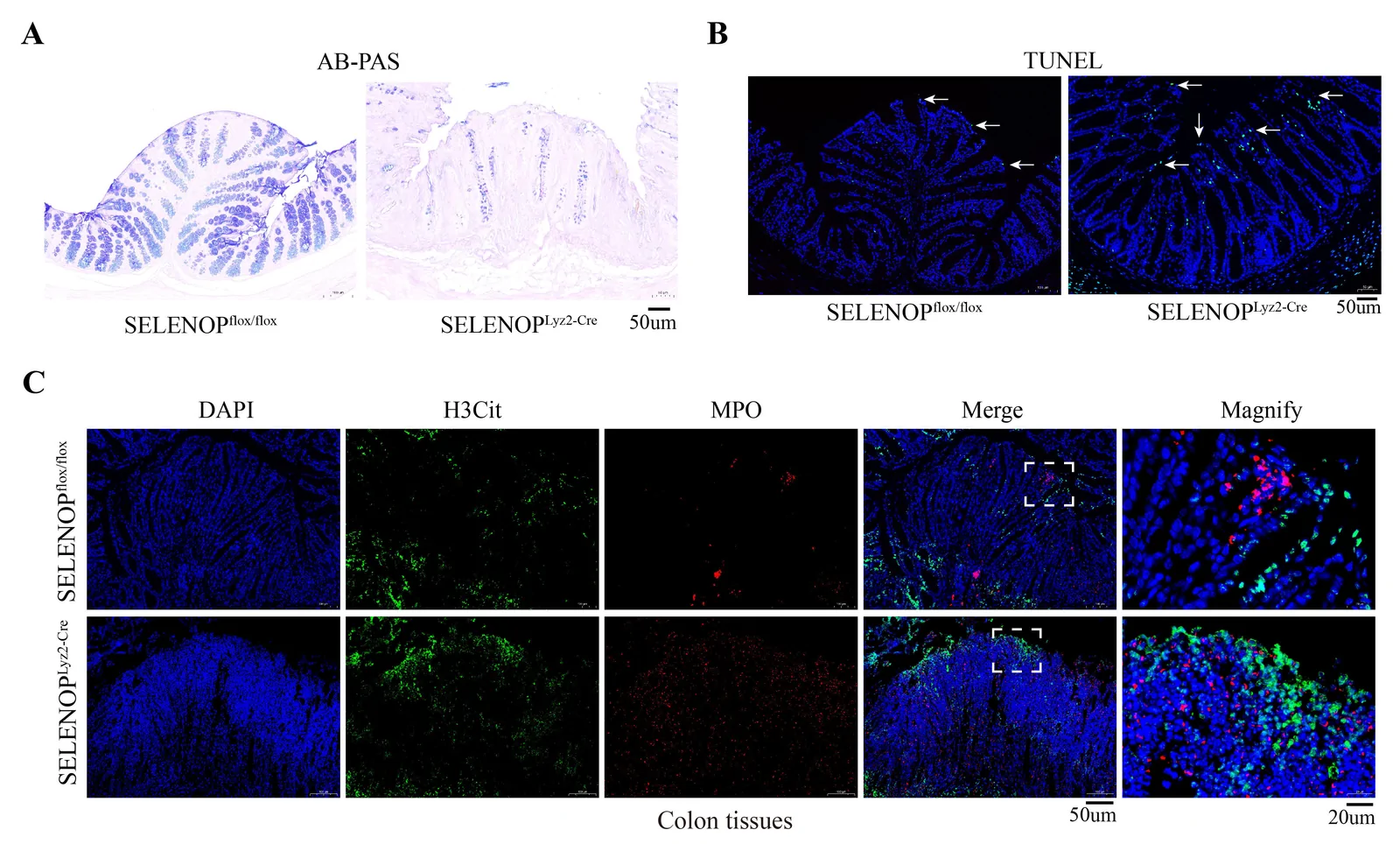
Myeloid SELENOP deficiency impairs mucus barrier integrity and promotes epithelial apoptosis and NET formation. (A) AB-PAS staining showing mucus layer integrity in colonic tissues from SELENOP^flox/flox^ and SELENOP^Lyz2−Cre^ mice. (B) TUNEL staining showing epithelial apoptosis in colonic tissues. (C) Immunofluorescence staining of MPO and H3Cit in colon tissues, with DAPI nuclear counterstaining. Representative images and magnified views are shown. AB-PAS, Alcian blue–periodic acid–Schiff; TUNEL, terminal deoxynucleotidyl transferase dUTP nick end labeling; MPO, myeloperoxidase; H3Cit, citrullinated histone H3; DAPI, 4′,6-diamidino-2-phenylindole.

We next assessed whether myeloid SELENOP contributes to epithelial barrier maintenance. MUC2 (Fig. 8J) and AB-PAS (Fig. S7A) staining showed impaired mucus barrier integrity in SELENOP^Lyz2−Cre^ mice. Functional assays further demonstrated that loss of myeloid SELENOP led to increased mannitol flux, decreased transepithelial resistance, and higher serum FITC-dextran levels, indicating enhanced intestinal permeability (Fig. 8K–M). At the molecular level, expression of the tight junction proteins Occludin and ZO-1 was markedly reduced in colonic tissues from knockout mice (Fig. 8N). Immunofluorescence confirmed disrupted localization and continuity of these proteins along the epithelial lining (Fig. 8O), while transmission electron microscopy revealed overt tight-junction ultrastructural abnormalities in SELENOP^Lyz2−Cre^ mice (Fig. 8P). In addition, increased epithelial microbial translocation (Fig. 8Q) and epithelial apoptosis (Fig. S7B) were observed in colonic tissues from SELENOP^Lyz2−Cre^ mice (Fig. 8Q).

Given the known antioxidant properties of SELENOP, we further examined redox-related pathways in the colon. Myeloid SELENOP deficiency suppressed the NRF2/HO-1/NQO-1 antioxidant axis (Fig. 8R), accompanied by increased TBARS, and MPO activity (Fig. 8S and T), and MPO/H3Cit immunofluorescence (Fig. S7C) indicating enhanced oxidative stress and inflammatory tissue injury. Collectively, these findings demonstrate that myeloid-derived SELENOP protects against TNBS-induced colitis by limiting inflammatory responses, preserving epithelial barrier integrity, and maintaining mucosal redox homeostasis.

### 3.9 SELENOP overexpression ameliorates colitis in IL-10-deficient mice in a macrophage-dependent manner

**Fig. 9:**
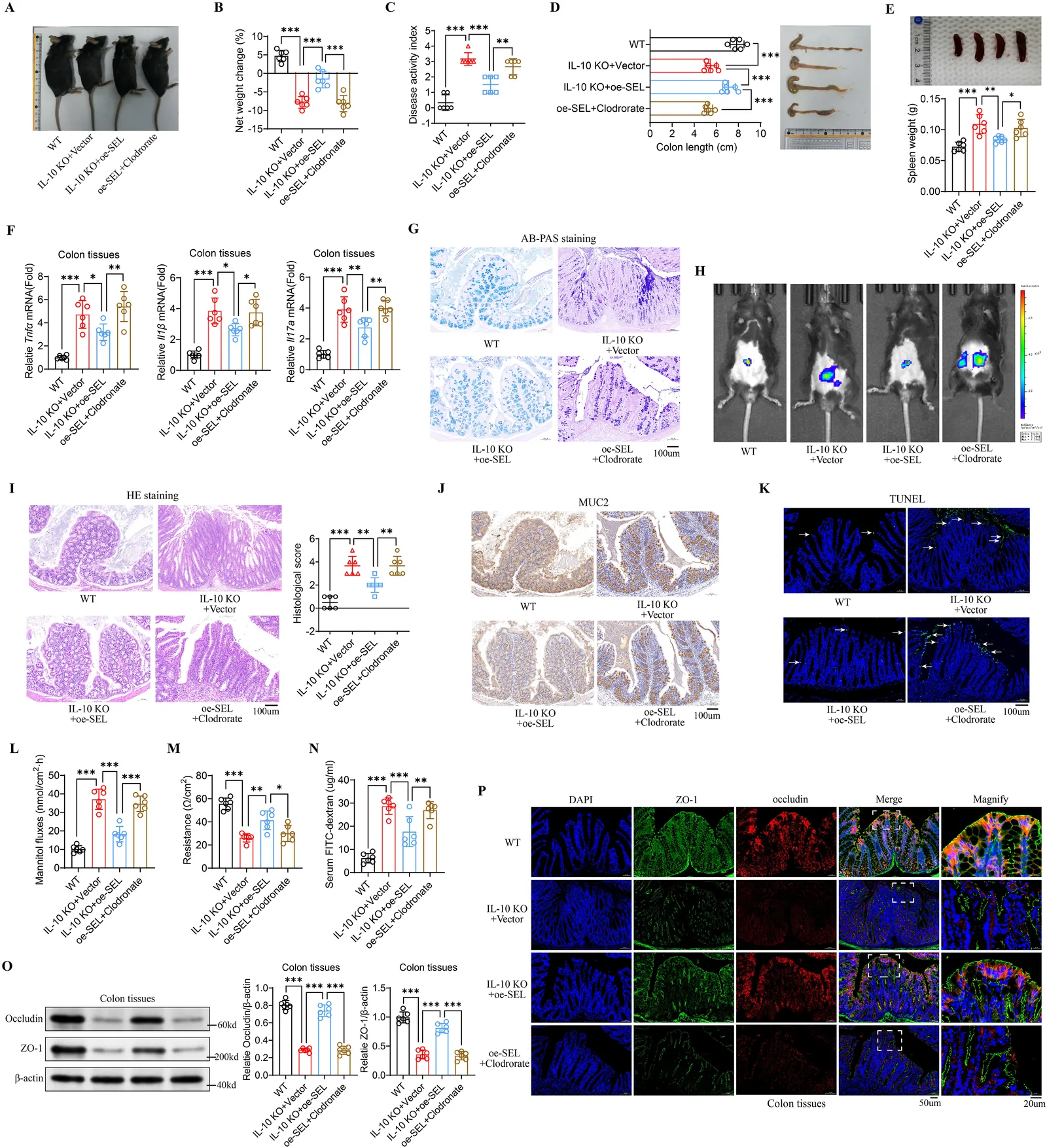
SELENOP overexpression ameliorates colitis in IL-10-deficient mice in a macrophage-dependent manner. (A) Representative gross appearance of WT, IL-10 KO + Vector, IL-10 KO + oe-SEL, and oe-SEL + Clodronate mice. (B–D) Net weight change (B), disease activity index (C), and colon length (D) in the indicated groups. (E) Representative spleen images and spleen weight in the indicated groups. (F) Relative colonic Tnfα, Il1β, and Il17a mRNA expression in the indicated groups. (G) Representative AB-PAS staining of colonic tissues from the indicated groups. Scale bar, 100 μm (H) Representative in vivo imaging of neutrephil infiltration in the indicated groups. (I) Representative H&E staining of colonic tissues and histological scores in the indicated groups. Scale bar, 100 μm. (J-K) Representative MUC2 immunohistochemical (J) and TUNEL (K) staining (J) of colon tissues from the indicated groups. Scale bar, 100 μm. (L–N) Mannitol fluxes (L), transepithelial resistance (M), and serum FITC-dextran levels (N) in the indicated groups. (O) Representative western blotting (O) and immunofluorescence staining of Occludin and ZO-1 in colonic tissues from the indicated groups. Scale bars, 50 μm and 20 μm. WT, wild type; KO, knockout; AB-PAS, Alcian blue-periodic acid-Schiff; H&E, haematoxylin and eosin; MUC2, mucin 2; TUNEL, terminal deoxynucleotidyl transferase dUTP nick end labelling; FITC, fluorescein isothiocyanate; ZO-1, zonula occludens-1; DAPI, 4′,6-diamidino-2-phenylindole. Data are presented as mean ± SD. Statistical analysis was performed using one-way ANOVA followed by Tukey's post hoc test. *P < 0.05; **P < 0.01; ***P < 0.001.

To further define the protective role of SEL in chronic intestinal inflammation, we evaluated SEL overexpression (oe-SEL) in IL-10-deficient mice, with or without clodronate-mediated macrophage depletion (Fig. 9). Gross examination showed that IL-10 knockout mice developed a severe wasting phenotype, whereas oe-SEL partially improved overall condition; this benefit was lost after clodronate treatment (Fig. 9A). Consistently, oe-SEL significantly reduced body weight loss, lowered DAI, and preserved colon length, while macrophage depletion largely abolished these effects (Fig. 9B–D). Likewise, splenomegaly was alleviated by oe-SEL but reappeared after clodronate administration (Fig. 9E). At the molecular level, oe-SEL markedly reduced colonic expression of Tnfα, Il1β, and Il17a, whereas these anti-inflammatory effects were weakened by macrophage depletion (Fig. 9F). In addition, AB-PAS staining showed improved goblet cell-associated mucus production, and in vivo imaging confirmed reduced inflammatory burden in oe-SEL-treated IL-10-deficient mice; both effects were substantially reversed by clodronate treatment (Fig. 9G and H).

**Fig. S8:**
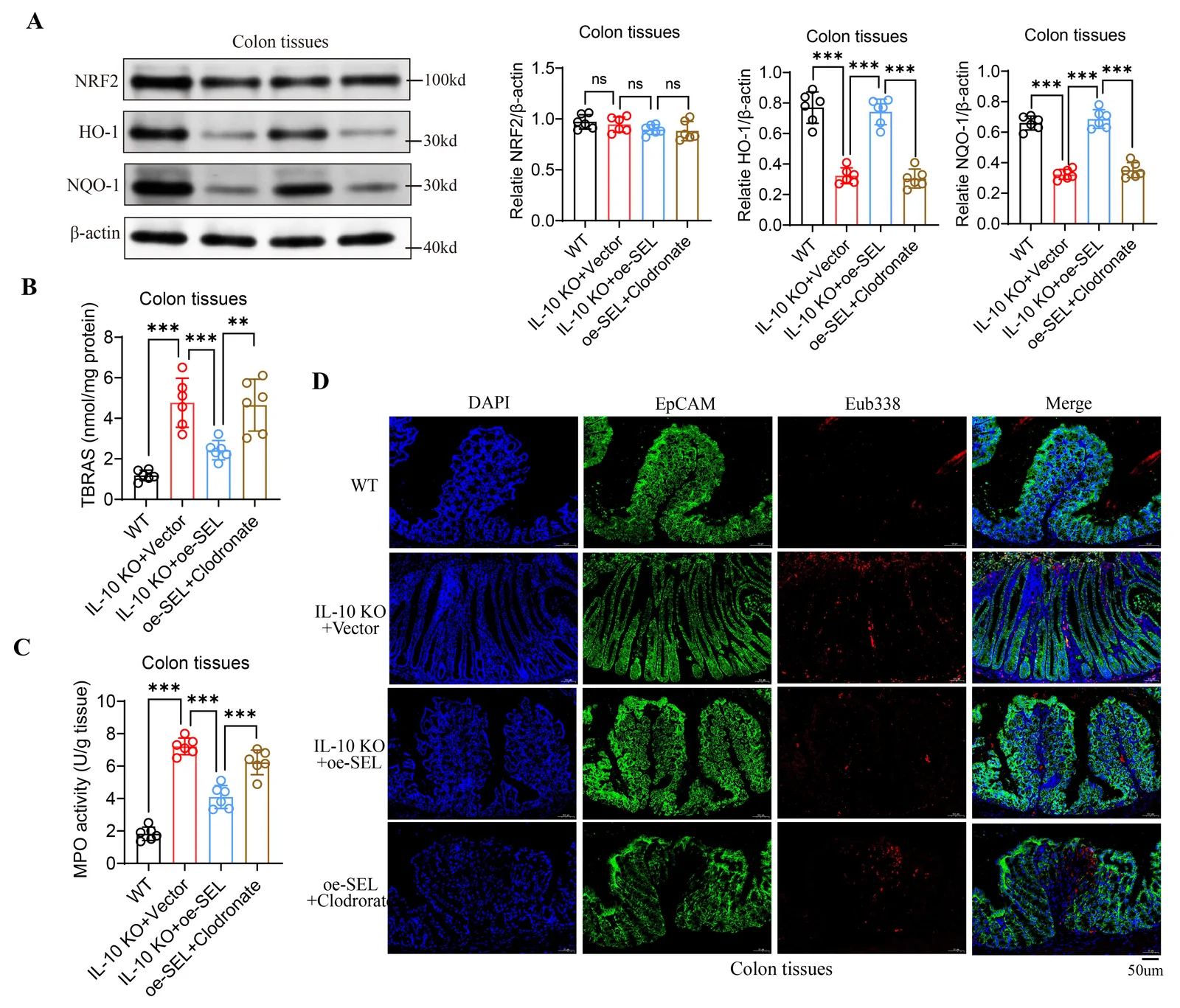
SELENOP overexpression attenuates oxidative stress and bacterial translocation in IL-10-deficient colitis. (A) Western blot analysis of NRF2/HO-1/NQO-1 pathway, with quantification. (B) TBRAS levels in colon tissues under the indicated conditions. (C) MPO activity in colon tissues. (D) Immunofluorescence staining of bacterial probe Eub338 and epithelial marker EpCAM in colon tissues. Scale bar: 50μm TBARS, thiobarbituric acid reactive substances; MPO, myeloperoxidase; NRF2, nuclear factor erythroid 2-related factor 2; HO-1, heme oxygenase 1; NQO1, NAD(P)H quinone dehydrogenase 1. Data are presented as mean ± SD. Statistical analysis was performed using one-way ANOVA followed by Tukey's post hoc test. *P < 0.05; **P < 0.01; ***P < 0.001.

Further histological and functional analyses supported a barrier-protective role for SEL. H&E staining showed that oe-SEL markedly alleviated epithelial injury, crypt damage, and inflammatory infiltration in IL-10-deficient colons, with lower histological scores; these improvements were again blunted by macrophage depletion (Fig. 9I). MUC2 staining demonstrated restoration of mucus-producing epithelial cells by oe-SEL (Fig. 9J), while TUNEL analysis showed reduced epithelial apoptosis (Fig. 9K). Functionally, oe-SEL decreased mannitol permeability, increased transepithelial resistance, and reduced serum FITC-dextran leakage, indicating improved epithelial barrier function (Fig. 9L–N). At the protein level, oe-SEL restored Occludin and ZO-1 expression, whereas clodronate weakened this recovery (Fig. 9O). Immunofluorescence further confirmed improved localization and continuity of tight junction proteins in the oe-SEL group, with clear disruption after macrophage depletion (Fig. 9P). Collectively, these data indicate that SEL exerts a protective effect in IL-10-deficient colitis by suppressing inflammation, reducing epithelial apoptosis, and restoring mucus and tight-junction integrity, and that these benefits are largely macrophage-dependent. Consistent with these findings, oe-SEL restored the expression of antioxidant pathway components, including HO-1 and NQO1, without significantly altering NRF2 levels, suggesting modulation of downstream antioxidant responses (Fig. S8A). Moreover, oe-SEL markedly attenuated oxidative stress in colonic tissues, as evidenced by reduced TBARS levels and MPO activity, whereas these effects were partially reversed upon macrophage depletion (Fig. S8B–C). Eub338 fluorescence in situ hybridization showed that oe-SEL reduced mucosal bacterial translocation, whereas this effect was reversed by clodronate treatment (Fig. S8D).

**Fig. 10:**
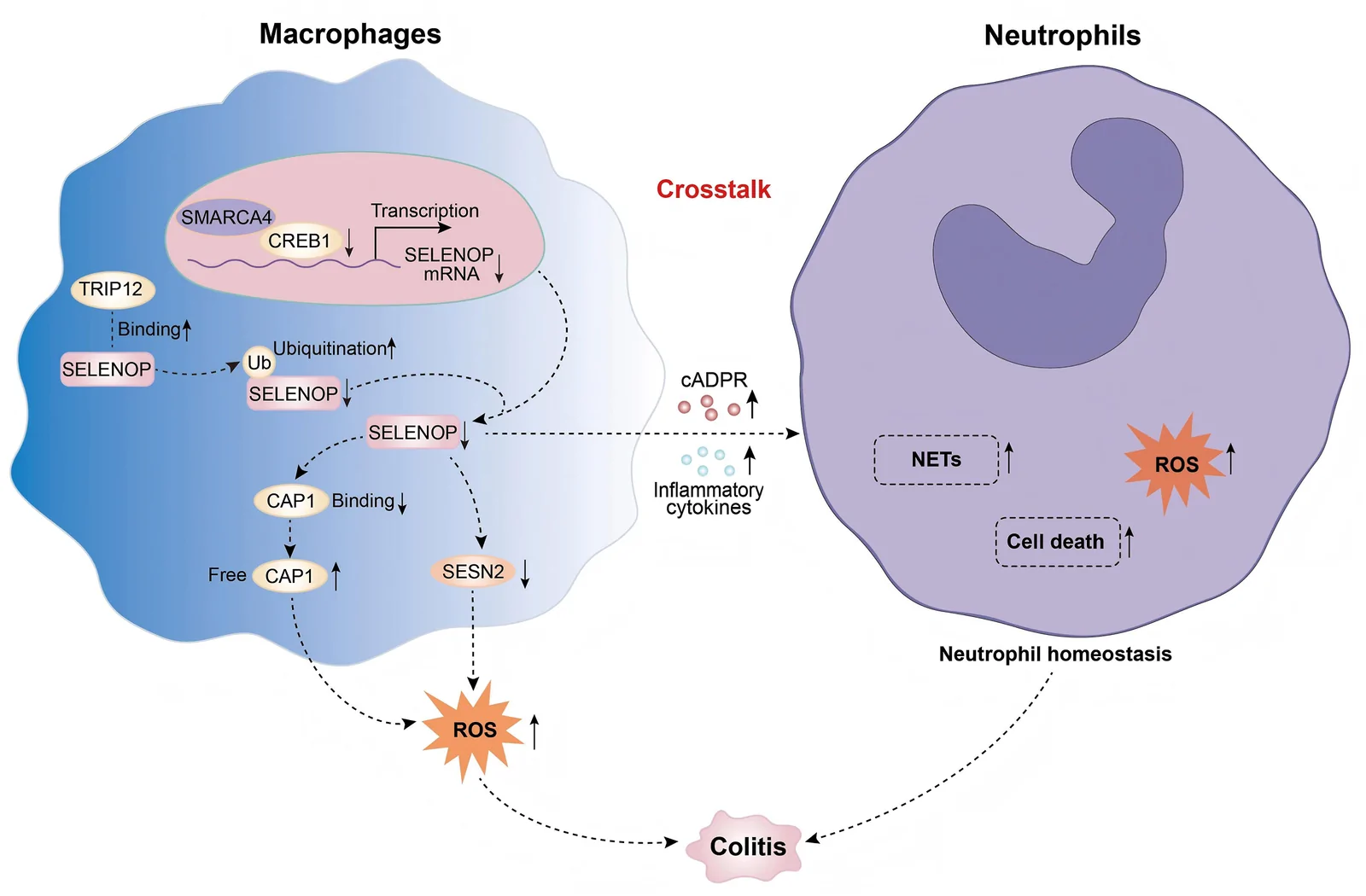
Schematic model of the SELENOP-mediated macrophage–neutrophil crosstalk in colitis. ROS, reactive oxygen species; cADPR, cyclic ADP-ribose; CAP1, cyclase-associated protein 1; SESN2, sestrin 2; NETs, neutrophil extracellular traps.

Collectively, our findings support a model in which macrophage-derived SELENOP serves as a protective hub linking intracellular redox regulation to macrophage–neutrophil crosstalk during colitis. In macrophages, SELENOP is positively regulated by the SMARCA4/CREB1 axis, whereas TRIP12-mediated ubiquitination limits its protein abundance. Loss of SELENOP weakens its association with CAP1 and is accompanied by reduced SESN2, thereby promoting macrophage ROS accumulation and inflammatory activation. In parallel, SELENOP-deficient macrophages release increased inflammatory cytokines and cADPR-associated signals, which disrupt neutrophil homeostasis and drive excessive ROS production, NET formation, and cell death. This pathogenic macrophage–neutrophil circuit amplifies mucosal inflammation, compromises epithelial barrier integrity, and accelerates colitis progression. Conversely, restoration of SELENOP interrupts this cascade and preserves intestinal homeostasis, identifying SELENOP as a key immunometabolic checkpoint with therapeutic potential in colitis (Fig. 10).

## 4 Discussion

The present study identifies macrophage-derived SELENOP as a previously underappreciated regulator of intestinal immune homeostasis in CD. By integrating single-cell transcriptomics, mechanistic in vitro assays, metabolomic analyses, and complementary in vivo models, we show that SELENOP is selectively downregulated in macrophages from inflamed colonic tissues. This cell type-specific reduction is clinically meaningful, as macrophage SELENOP expression was inversely associated with CRP, CDAI, and SES-CD, and lower SELENOP expression predicted a higher rate of postoperative endoscopic recurrence. These findings position macrophage-derived SELENOP not only as a mechanistically relevant molecule in CD pathogenesis but also as a candidate biomarker linked to disease activity and prognosis. This interpretation is consistent with recent literature highlighting the central role of intestinal macrophages in IBD pathobiology and emphasizing that disease-relevant signals can be obscured in bulk-tissue analyses because of marked cellular heterogeneity [43,44].

A major conceptual advance of this work is the demonstration that SELENOP functions within macrophages as an endogenous brake on inflammatory and oxidative injury. Although selenium biology and selenoproteins have long been linked to mucosal inflammation and redox control [45], most prior work focused on systemic selenium deficiency, epithelial selenoproteins, or tumor-associated contexts rather than macrophage-intrinsic SELENOP in CD. Previous studies showed that epithelial-derived SELENOP is protective in colitis-associated carcinogenesis and that selenium/selenoprotein networks broadly contribute to intestinal redox balance and immune regulation [46]. Our data extend this field by showing that, in macrophages, loss of SELENOP aggravates LPS/ATP-induced inflammatory responses, ROS accumulation, oxidative stress, and apoptosis, while impairing antioxidant defenses. Taken together, these findings provide evidence that macrophage-derived SELENOP may have a cell-specific protective role in intestinal inflammation.

Mechanistically, our findings support a model in which SELENOP protects macrophages at least partly through the SESN2/NRF2 axis. SESN2 is increasingly recognized as a stress-inducible regulator that coordinates antioxidant adaptation, metabolic homeostasis, and cytoprotection under inflammatory or oxidative conditions [47]. In parallel, NRF2 is widely viewed as a master transcriptional regulator of antioxidant responses and an important determinant of epithelial and immune homeostasis in IBD [48]. In our study, SELENOP deficiency reduced SESN2 expression, suppressed NRF2 nuclear accumulation, and diminished HO-1 and NQO1 expression, whereas restoring SESN2 partly rescued these defects and attenuated inflammatory and oxidative injury. These data suggest that SELENOP acts upstream of a SESN2-dependent antioxidant program that preserves macrophage redox fitness during inflammatory stress. Notably, our gain- and loss-of-function experiments argue that this is not simply a correlation but a functional signaling axis. Given the growing interest in NRF2-directed interventions in intestinal inflammation, the SELENOP–SESN2–NRF2 cascade may represent a therapeutically tractable antioxidant checkpoint in CD. In this study, lipid peroxidation was assessed using a TBARS-based assay, which is widely used as an indicator of oxidative stress. However, TBARS is not entirely specific for MDA, as thiobarbituric acid can also react with other aldehydic products [49,50]. Therefore, TBARS measurements should be interpreted as an estimation of MDA-related lipid peroxidation rather than absolute MDA quantification. More specific approaches, such as LC-MS-based MDA detection, may provide more accurate evaluation in future studies. Nevertheless, TBARS analysis was used here for comparative assessment of oxidative stress, and the results were consistent with other redox-related findings supporting the role of SELENOP in inflammatory regulation.

Another notable finding is the identification of CAP1 as a SELENOP-interacting partner required for the anti-inflammatory and anti-oxidative effects of SELENOP. CAP1 did not alter SELENOP abundance, but the interaction between the two proteins was strengthened under LPS/ATP stimulation and attenuated by NAC, suggesting that oxidative stress dynamically enhances their association. Functionally, CAP1 deficiency largely abolished the ability of SELENOP to promote NRF2 activation and suppress NLRP3 inflammasome signaling, GSDMD cleavage, and caspase-1 activation. These results connect SELENOP to the oxidative stress–inflammasome axis, a pathway increasingly implicated in gastrointestinal inflammatory disorders. Because excessive ROS is a known trigger of NLRP3 activation and pyroptotic injury [51,52], our data suggest that SELENOP may limit inflammatory amplification not only by buffering redox stress but also by restraining ROS-sensitive inflammasome activation through CAP1-dependent mechanisms. This finding broadens current understanding of how antioxidant proteins interface with innate immune signaling in macrophages.

Our study also provides a dual-layer explanation for why SELENOP is reduced in inflamed macrophages. At the post-translational level, TRIP12 was identified as an E3 ligase that binds SELENOP and promotes its ubiquitin-dependent proteasomal degradation, which is enhanced during inflammatory stimulation. At the transcriptional level, CREB1 and SMARCA4 cooperatively activated SELENOP expression, whereas inflammatory conditions were associated with reduced CREB1 expression, lower CREB1 phosphorylation, and weakened CREB1–SMARCA4 interaction. Together, these findings indicate that inflammatory stress reduces SELENOP through both impaired transcriptional activation and accelerated protein turnover. This dual regulatory architecture is biologically plausible, as inflammatory programs often combine transcriptional repression with ubiquitin-mediated protein destabilization to ensure sustained loss of protective molecules [53,54]. More broadly, our results place SELENOP within a multilayered regulatory network that may help explain why antioxidant defenses collapse in inflamed macrophages despite the presence of compensatory stress signals. Beyond macrophage-intrinsic biology, this study highlights macrophage–neutrophil crosstalk as a critical downstream consequence of SELENOP loss. Neutrophils are increasingly recognized as major effectors of mucosal damage in IBD, particularly through ROS overproduction, delayed resolution, and extracellular trap formation [55,56]. Here, SELENOP-deficient macrophages drove neutrophil inflammatory activation, oxidative injury, apoptosis, and extracellular trap-like formation in coculture, accompanied by suppression of neutrophil NRF2 signaling. Metabolomic profiling further identified cADPR-associated changes in macrophage supernatants, and exogenous cADPR largely reversed the protective effects of SELENOP-overexpressing macrophages on neutrophils. Given the established role of the CD38–cADPR system in calcium mobilization and stress signaling [57], our data suggest that macrophage-derived SELENOP may protect neutrophils partly by limiting a metabolite-dependent oxidative amplification loop. This is an important extension of current models of innate immune dysregulation in CD, as it indicates that SELENOP does not merely regulate a single cell type but instead restrains a pathogenic intercellular circuit linking activated macrophages to neutrophil-mediated tissue damage.

The in vivo data strongly support the physiological relevance of these mechanisms. Myeloid-specific Selenop deletion aggravated TNBS colitis, leading to greater weight loss, worse survival, stronger inflammatory signals, more severe histological injury, enhanced oxidative stress, and profound barrier dysfunction. Conversely, SELENOP overexpression ameliorated disease severity in IL-10-deficient mice, and this benefit was largely lost after macrophage depletion, indicating that macrophages are a principal effector compartment for SELENOP-mediated protection. Particularly noteworthy is the consistent barrier phenotype observed across models, including reduced MUC2, lower Occludin and ZO-1 expression, increased permeability, higher bacterial translocation, and increased epithelial apoptosis. These findings suggest that myeloid-derived SELENOP protects the intestine not only by dampening inflammation but also by indirectly preserving epithelial barrier integrity and mucosal redox homeostasis. Given that oxidative stress and defective barrier repair are core features of IBD [58], this macrophage-centered mechanism has substantial translational relevance.

Several limitations should also be acknowledged. First, although our data identify SESN2, CAP1, TRIP12, CREB1, and SMARCA4 as key nodes in SELENOP regulation and function, the precise structural determinants of these interactions, including the dominant ubiquitination sites of SELENOP under inflammatory conditions, require further validation. Second, the cADPR findings support metabolite-mediated macrophage–neutrophil communication, but the upstream enzymatic source and the full spectrum of bioactive metabolites in SELENOP-deficient macrophage supernatants remain to be defined. Third, while the animal and *ex vivo* data support therapeutic relevance, it remains unclear whether recombinant SELENOP, selenium supplementation, or pharmacological stabilization of endogenous SELENOP would be the most feasible translational strategy in patients with CD. These issues warrant further investigation in organoid systems, human primary-cell platforms, and preclinical intervention studies.

In conclusion, our study establishes macrophage-derived SELENOP as a protective immunometabolic checkpoint in colitis. Inflammatory stress suppresses SELENOP through impaired CREB1/SMARCA4-dependent transcription and TRIP12-mediated proteasomal degradation. Loss of SELENOP disrupts CAP1- and SESN2/NRF2-associated antioxidant programs, enhances inflammasome activity and pyroptotic signaling, and drives pathogenic macrophage–neutrophil crosstalk characterized by cADPR-associated oxidative injury. These events converge on epithelial barrier disruption and worsening colitis. Restoring SELENOP activity, stabilizing its expression, or targeting its downstream redox and intercellular signaling pathways may therefore represent promising therapeutic strategies for CD.

## CRediT authorship contribution statement

**Honggang Wang:** Methodology, Investigation, Funding acquisition, Formal analysis, Data curation, Conceptualization. **Juan Wang:** Project administration, Investigation. **Wenliang Jiang:** Resources. **Chao Cheng:** Software. **Shaoqi Cheng:** Supervision. **Shuqiang Fu:** Project administration. **Yi Zhang:** Visualization. **Cuixia Liu:** Visualization, Resources, Methodology. **Jing Sun:** Writing – original draft, Visualization, Software. **Jie Zhao:** Writing – review & editing, Writing – original draft, Visualization, Validation, Supervision.

## Ethics declaration

Written informed consent to take part in the study and to publish the article has been obtained from all participants or their legal representatives. The privacy rights of participants have been observed.

This study included organ or tissue donors. This study includes human biological material and consent was obtained by donors, or their next of kin or legal representatives, for use in this study and for publication of the article. The samples used in this research were not sourced from executed prisoners or prisoners of conscience.

This study was performed in compliance with relevant laws, regulatory frameworks and guidelines where the research took place. This study was approved by the Ethics Committee of The First Affiliated Hospital with Nanjing Medical University. (Approval No. No. 2023-SR-115)

This study was conducted in accordance with the ARRIVE (Animal Research: Reporting of In Vivo Experiments) guidelines. This study was approved by the institutional ethics committee of Jiangsu Hanjiang Biotechnology. (Approval No. No. HJSW-25090501)

## Ethical approval and consent to participate

Consent to participate is not applicable in this study. The clinical study was approved by the ethics committee of The First Affiliated Hospital of Nanjing Medical University (No. 2023-SR-115). All procedures were approved by the institutional ethics committee of Jiangsu Hanjiang Biotechnology (No. HJSW-25090501) according to the guidelines by the Chinese Council on Animal Care.

## Consent for publication

Not applicable.

## Availability of data and material

Please contact the corresponding author upon reasonable request.

## Funding

This work was supported by 10.13039/501100001809National Natural Science Foundation of China (82670704, 82300609), 10.13039/501100010854Jiangsu Provincial Commission of Health and Family Planning (M2025006), Social Development Plan of Taizhou (TSL202501), Changzhou Sci&Tech Program (2022CZBJ066, CJ20245024), Changzhou Medical Center of 10.13039/501100007289Nanjing Medical University Program (CMC2024PY17), the “333" Engineering Project 10.13039/501100002949Jiangsu Province [(2024) 3-2417], 10.13039/501100007289Nanjing Medical University Wuxi Medical Center “Young Elite Talent Program” (WMCQ202605), Yanzhen Talent Program for Emerging Academic Leaders (YZ-HBDTR-SJ-2025).

## Declaration of competing interest

The authors declare that they have no known competing financial interests or personal relationships that could have appeared to influence the work reported in this paper.

## Data Availability

Data will be made available on request.
