# Supplementary material for: Macrophage SELENOP controls neutrophil homeostasis through redox stress regulation in Crohn's disease
Source: Redox Biol. 2026 Sep 9;97:104377. doi: 10.1016/j.redox.2026.104377 (PMC13587815; doi:10.1016/j.redox.2026.104377)
Supplement: Supplementary file 1 [file mmc5.pdf]

Fig 1

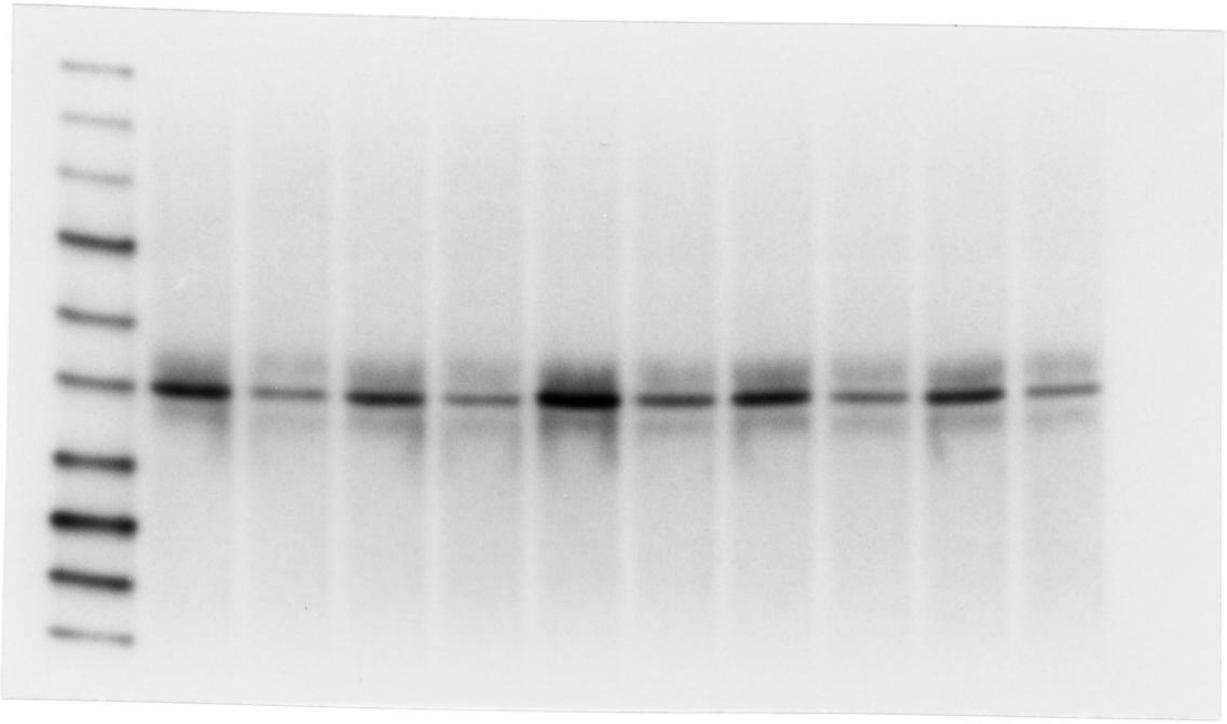

Fig 1G SELENOP

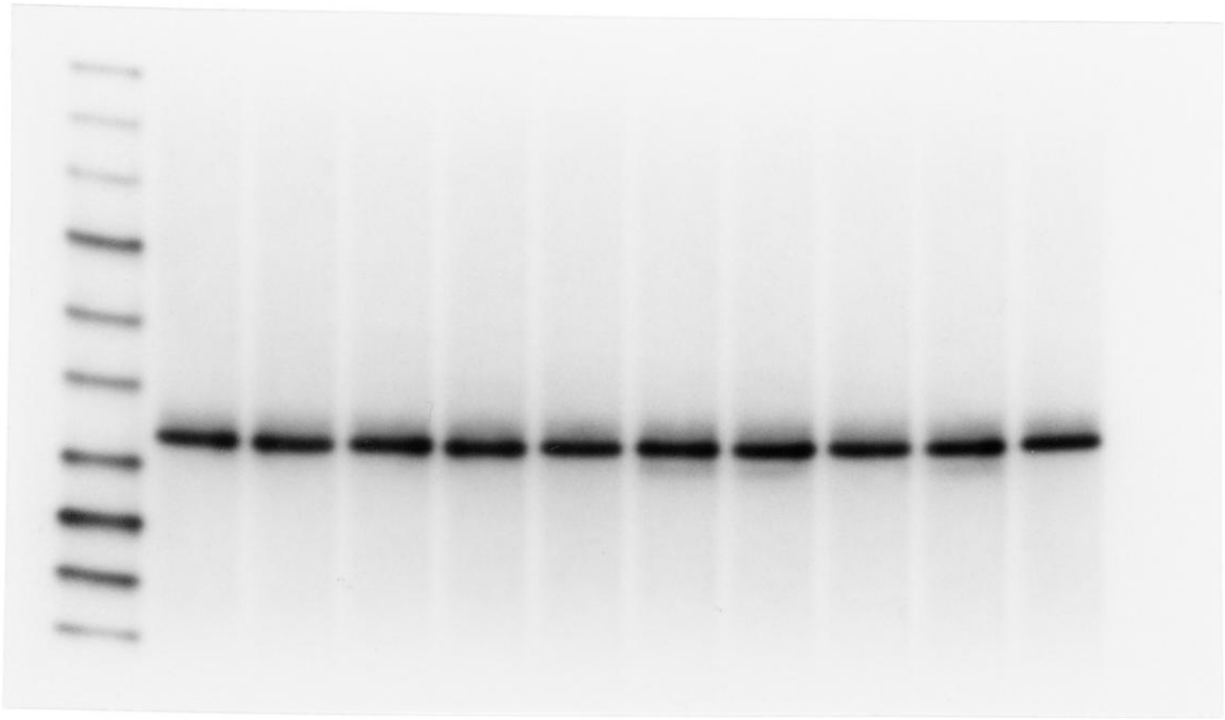

Fig 1G β-actin

Fig 2

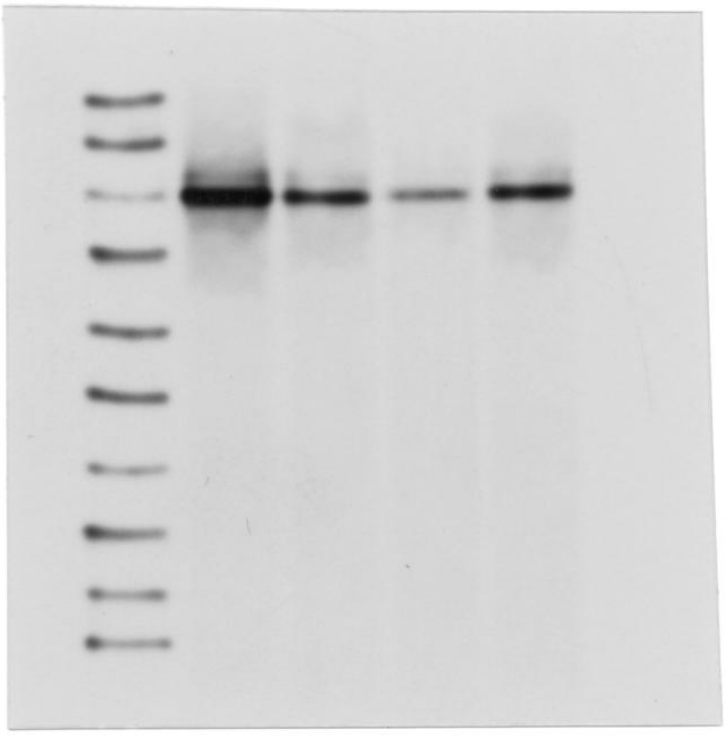

Fig 2J NRF2

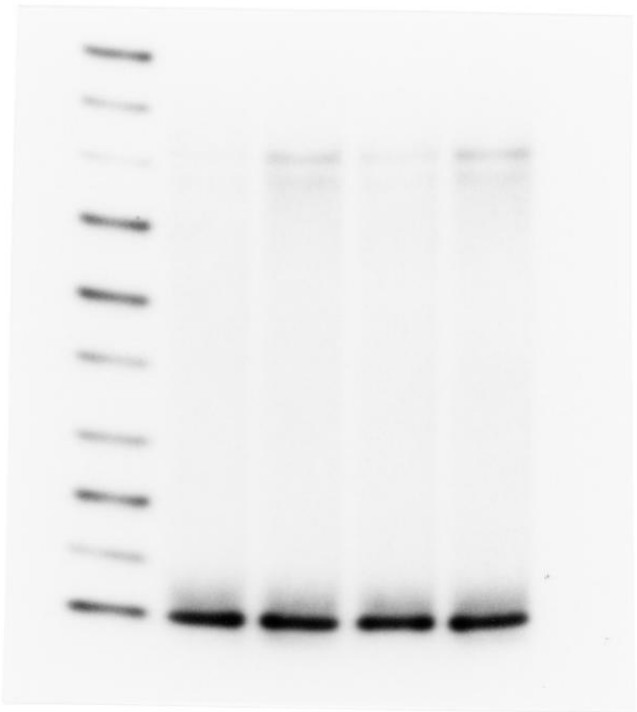

Fig 2J H3

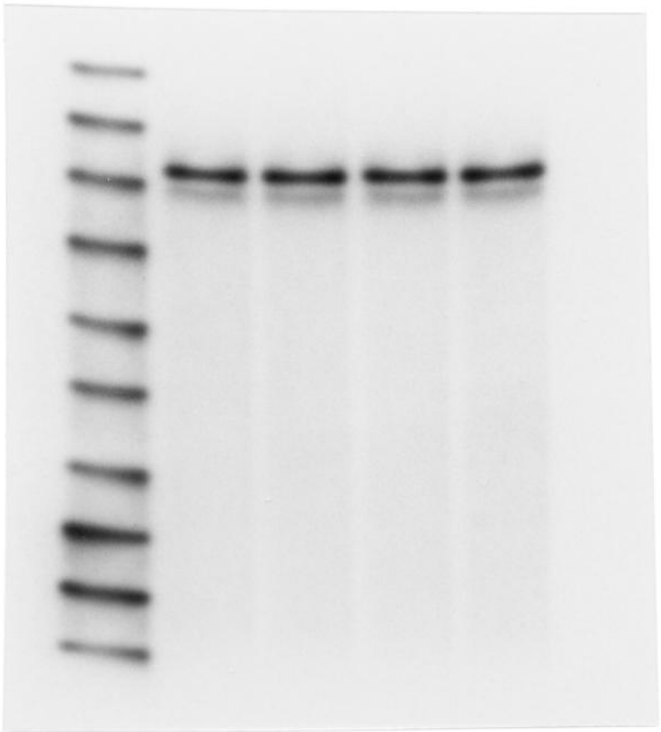

Fig 2J NRF2

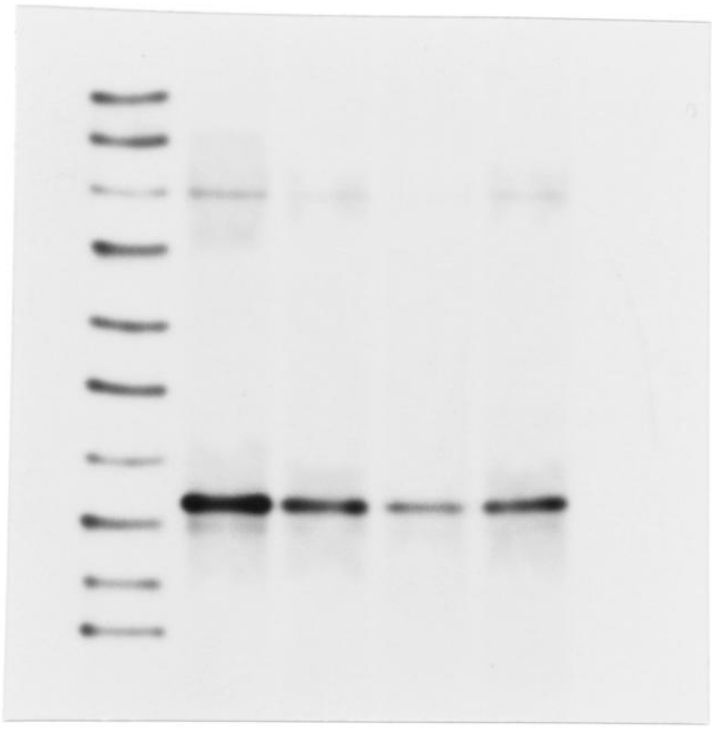

Fig 2J HO-1

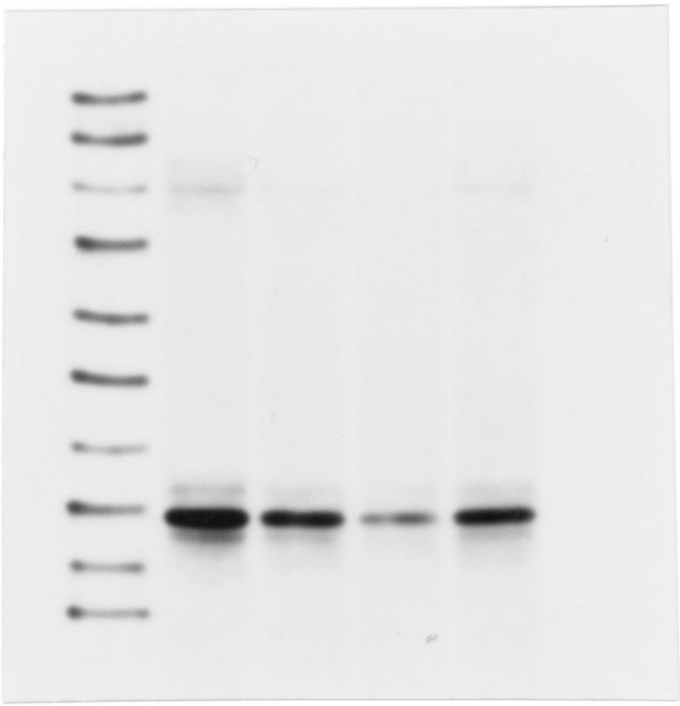

Fig 2J NQO-1

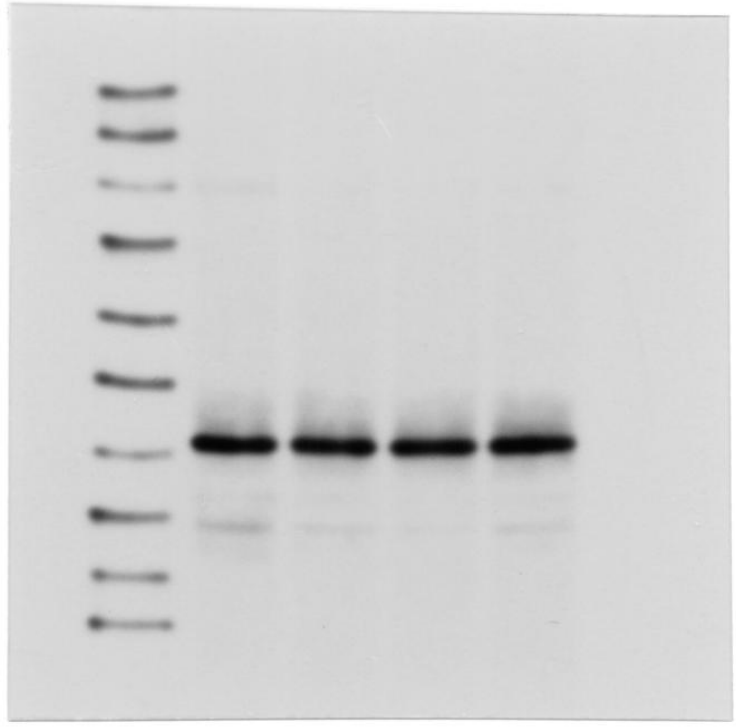

Fig 2J β-actin

Fig 2

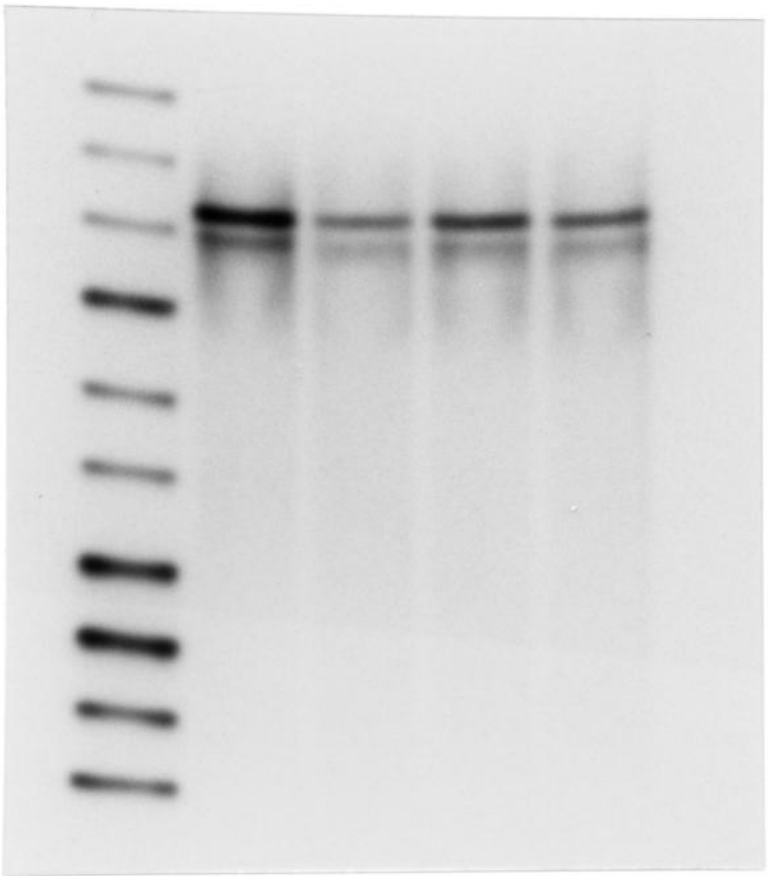

Fig 2M NRF2

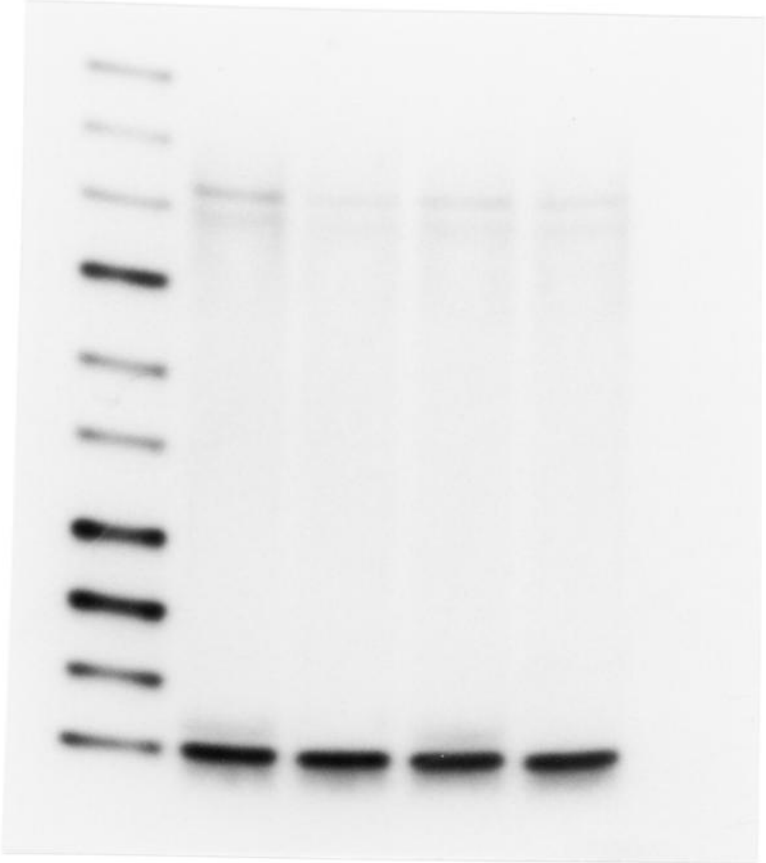

Fig2M H3

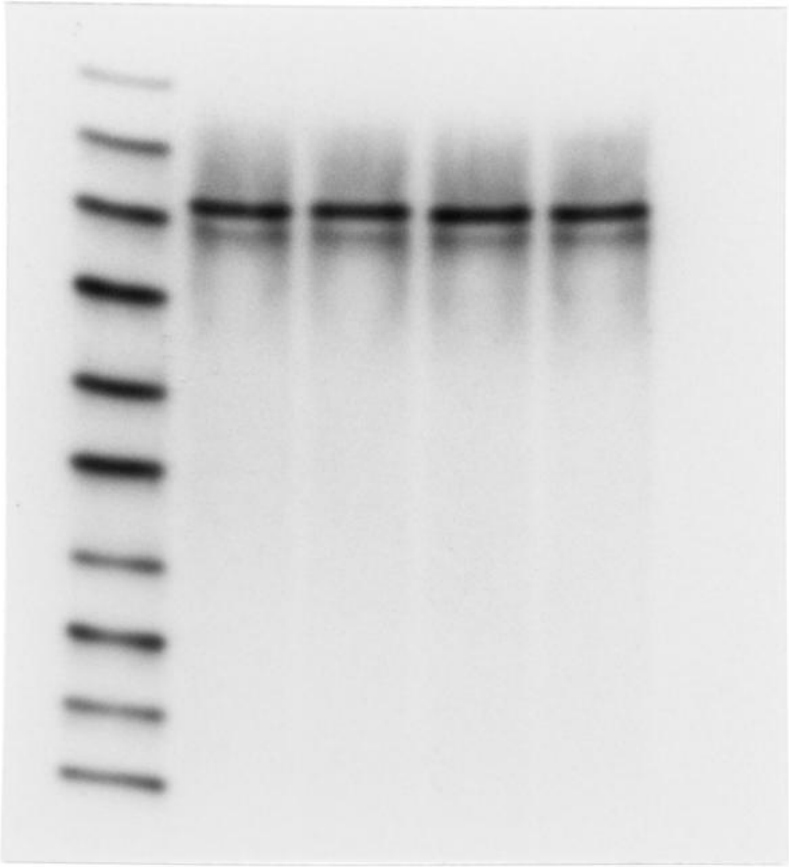

Fig 2M NRF2

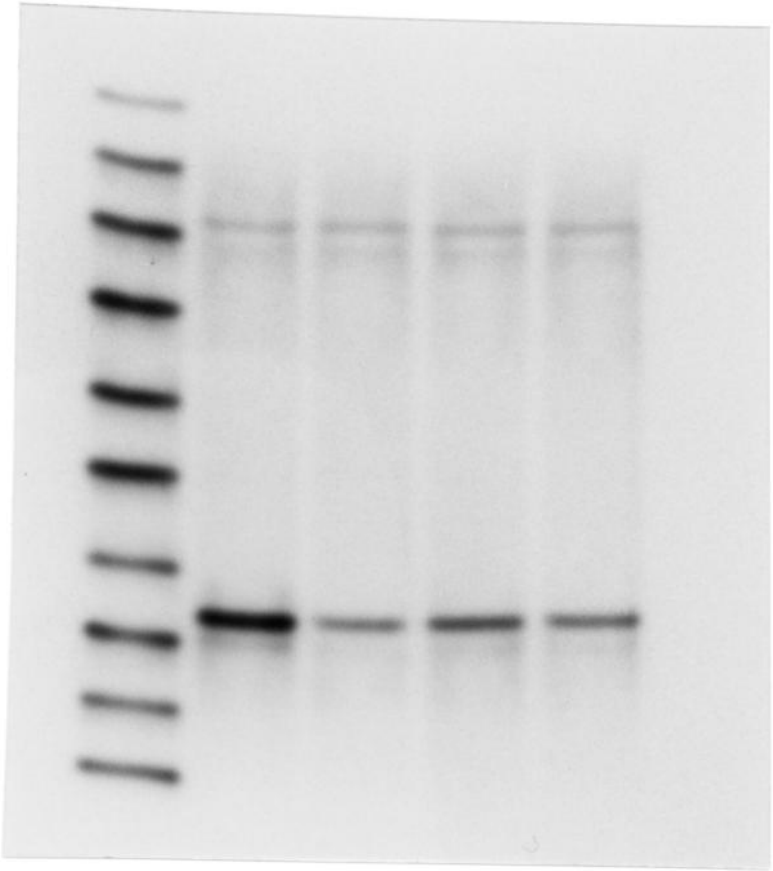

Fig 2M HO-1

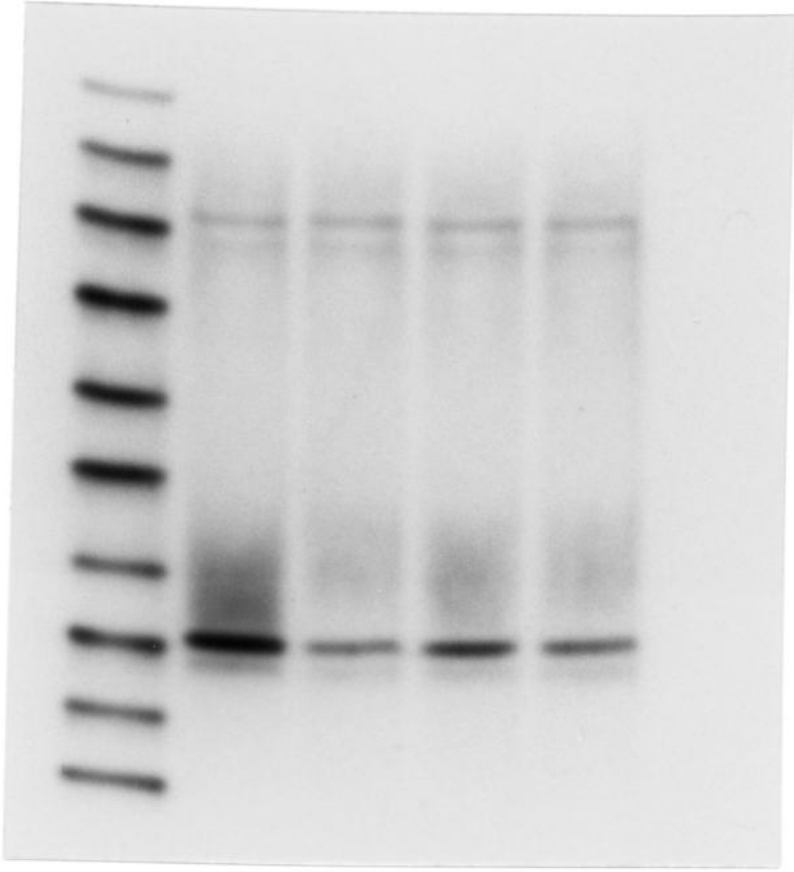

Fig2M NQO-1

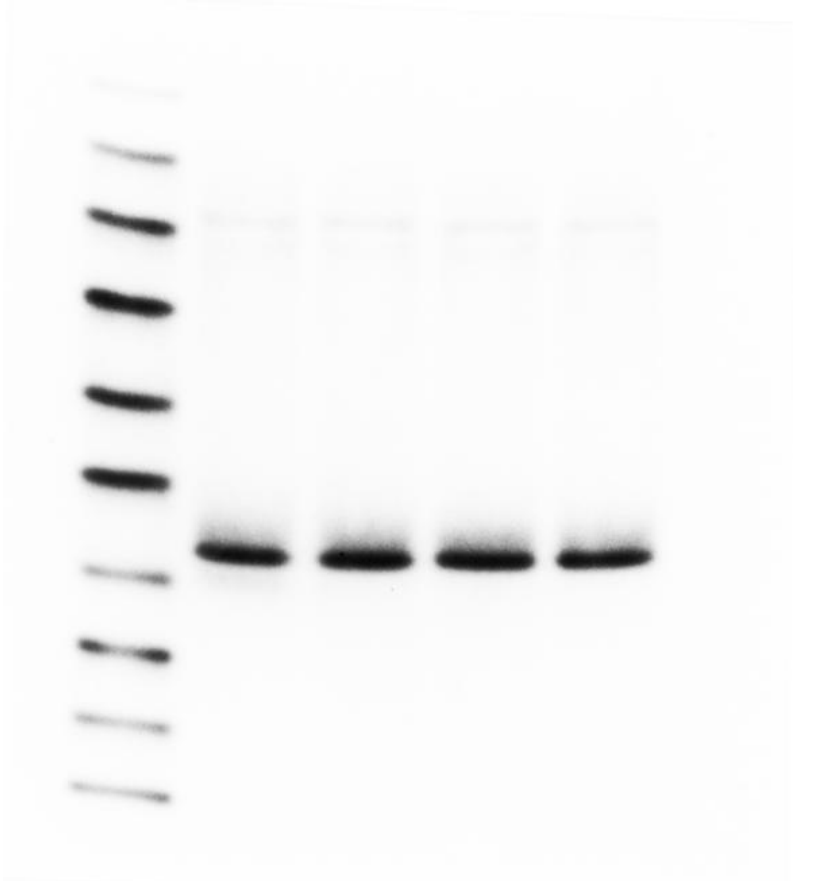

Fig 2M β-actin

Fig S2

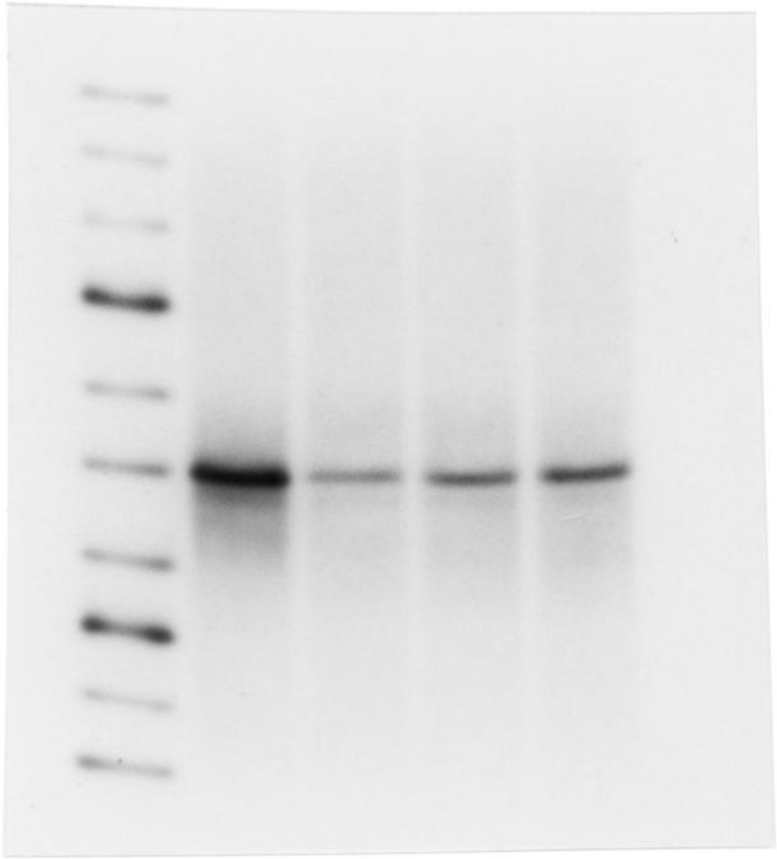

Fig S2B SELENOP

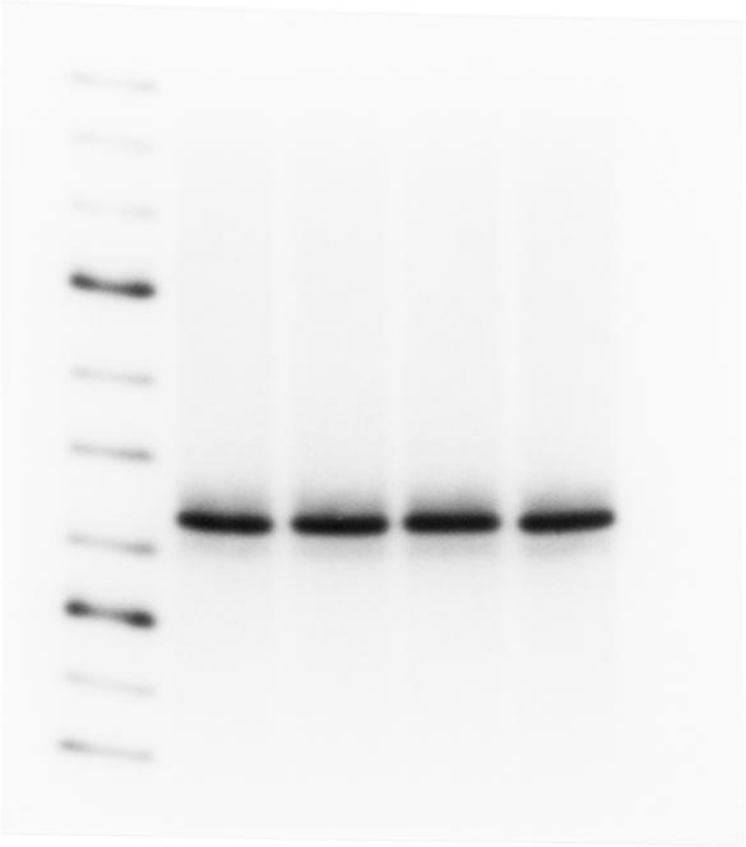

Fig S2B β-actin

Fig 3

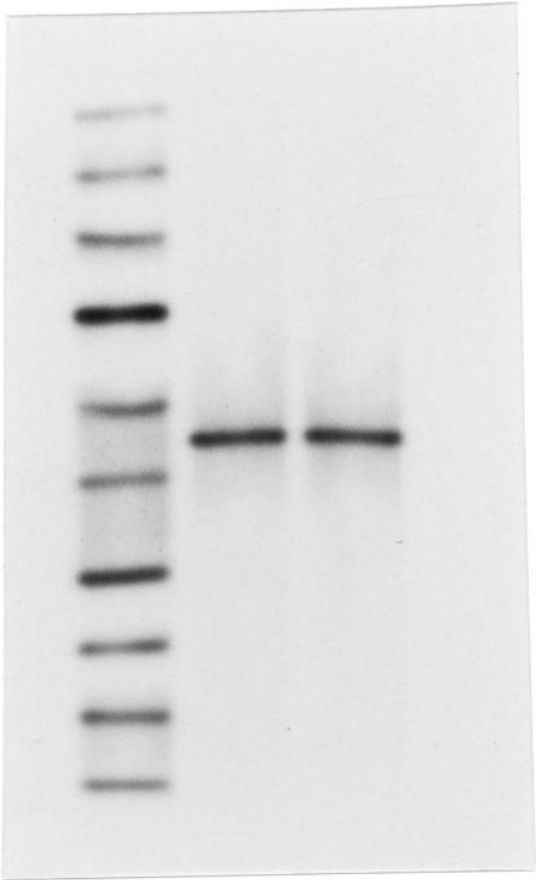

Fig 3B CAP1

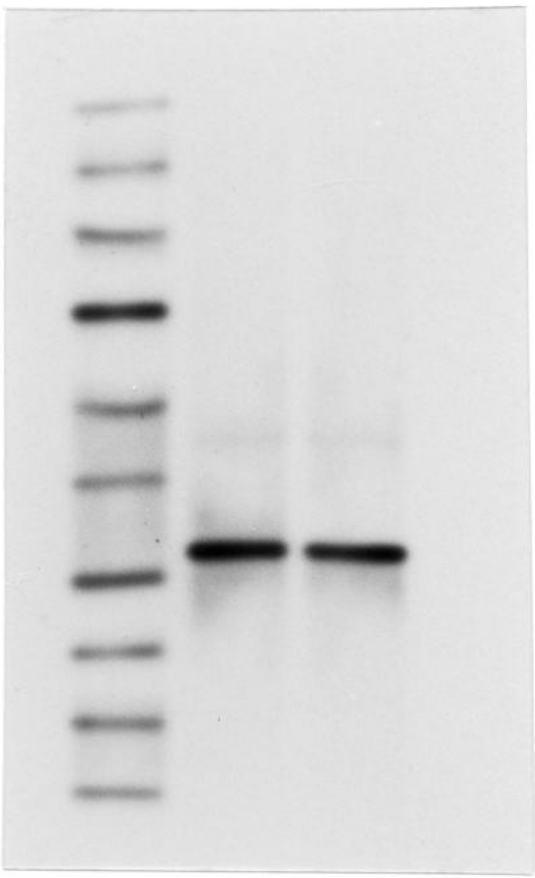

Fig 3B b-actin

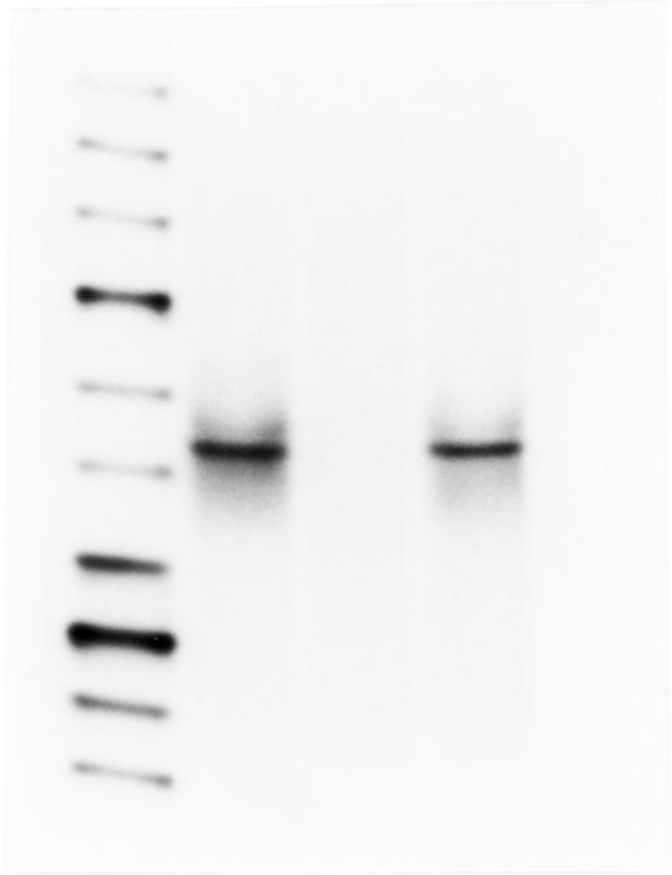

Fig 3D CAP1

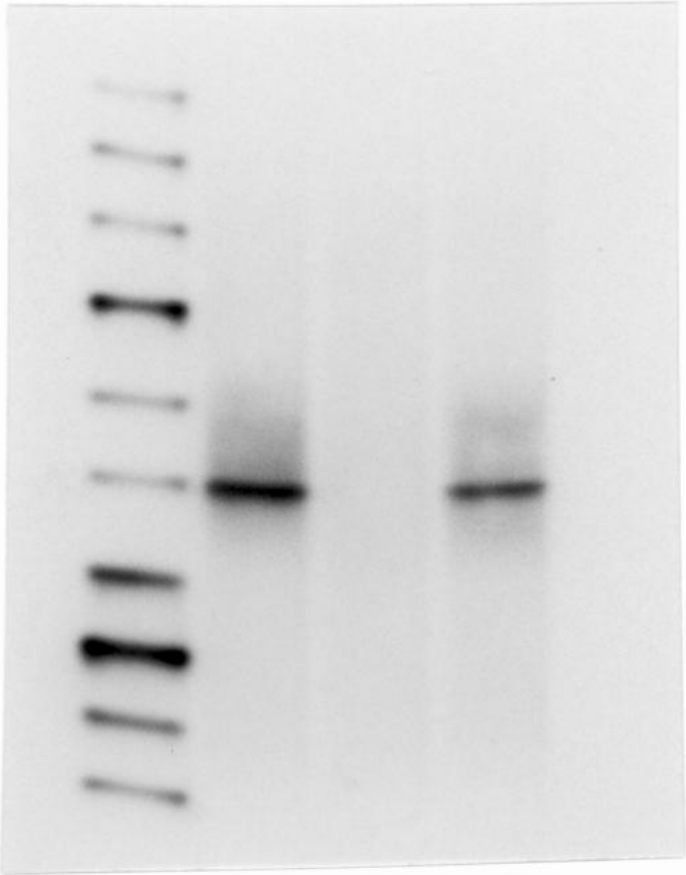

Fig 3D SELENOP

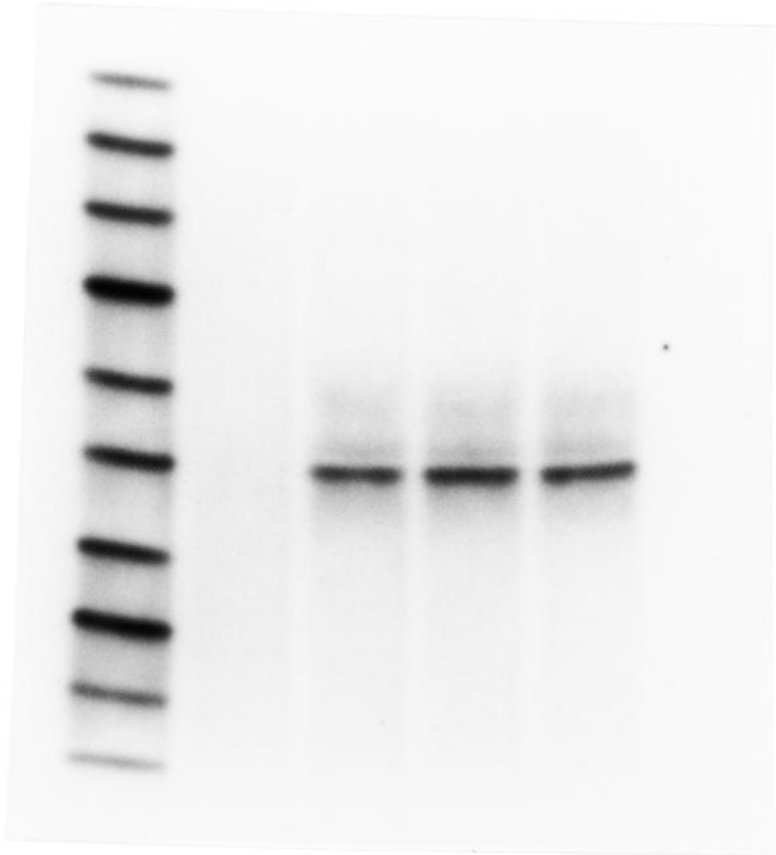

Fig 3G SELENOP

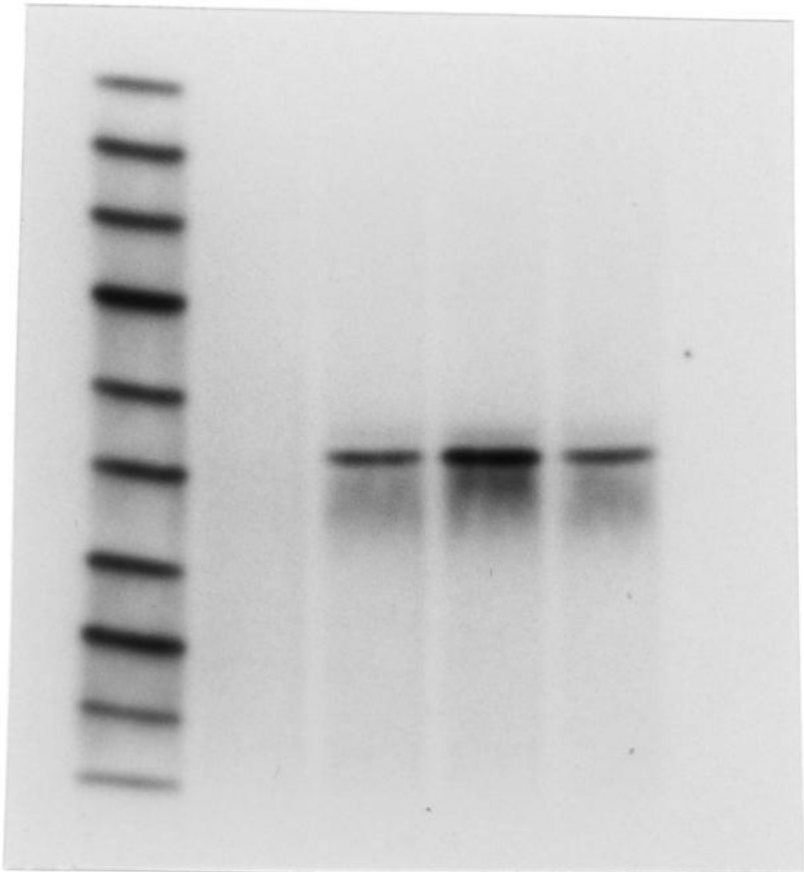

Fig 3G CAP1

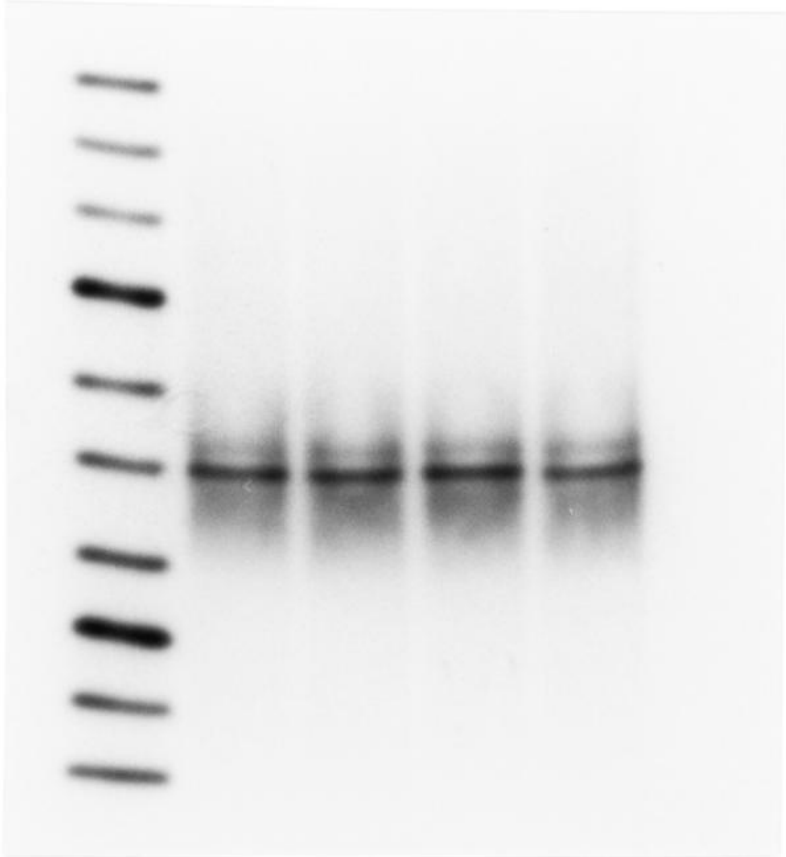

Fig 3G SELENOP

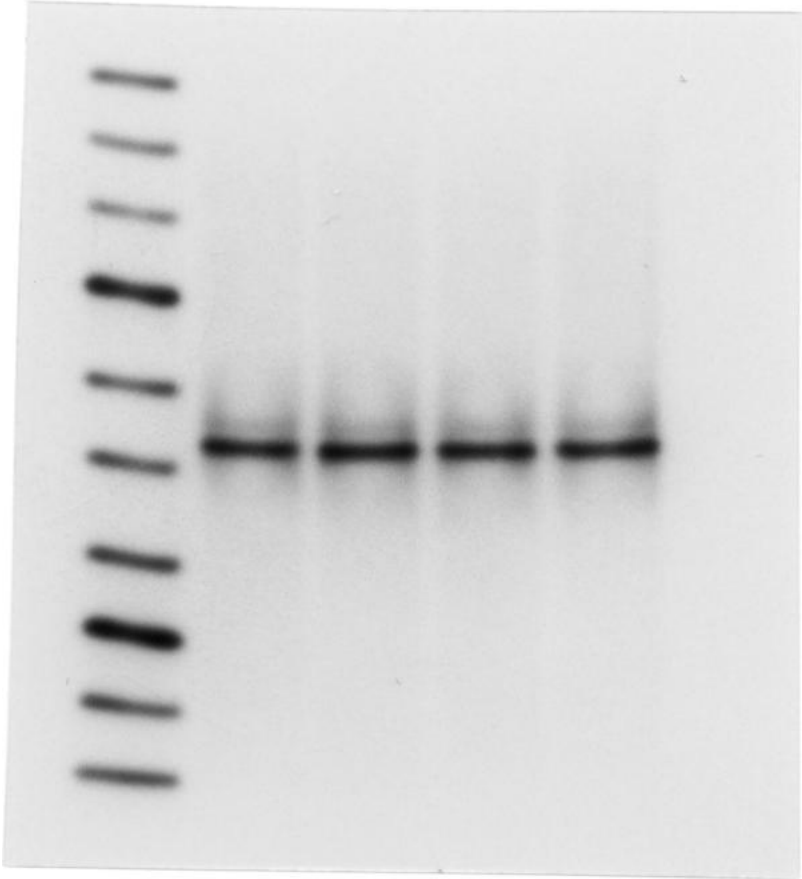

Fig 3G CAP1

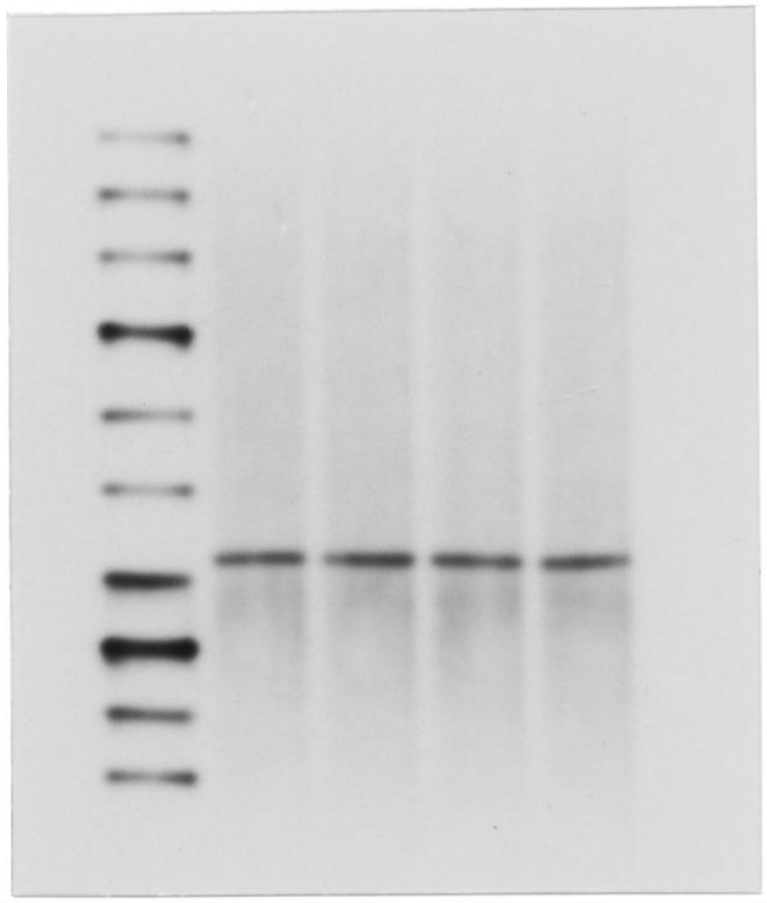

Fig 3G β-actin

Fig 3

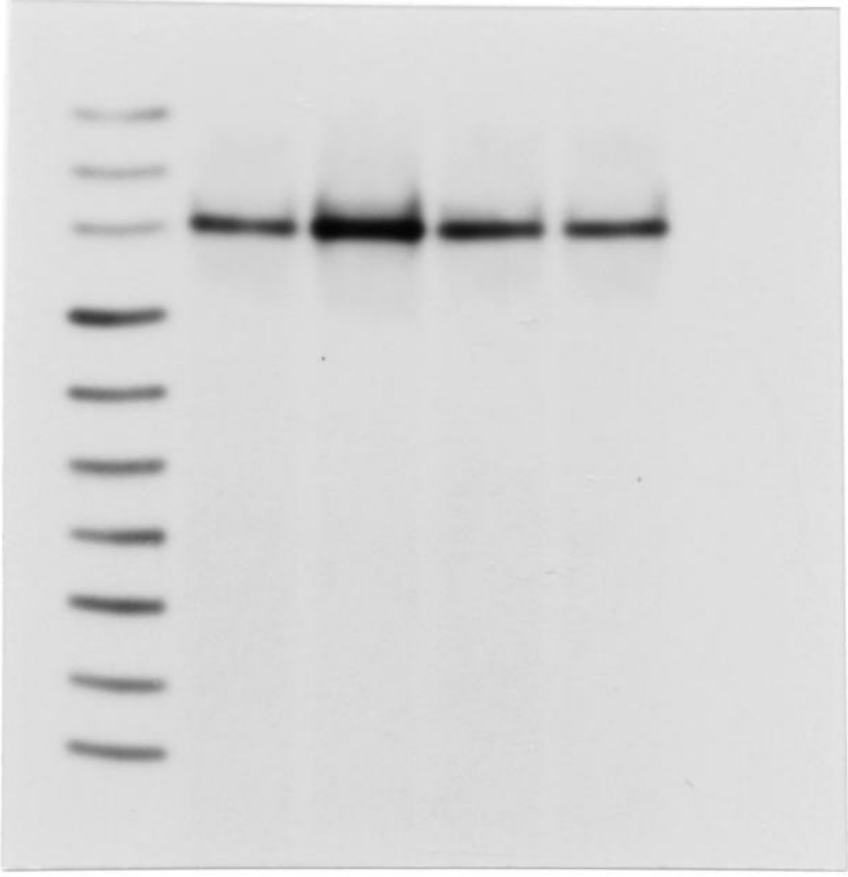

Fig 3I NRF2

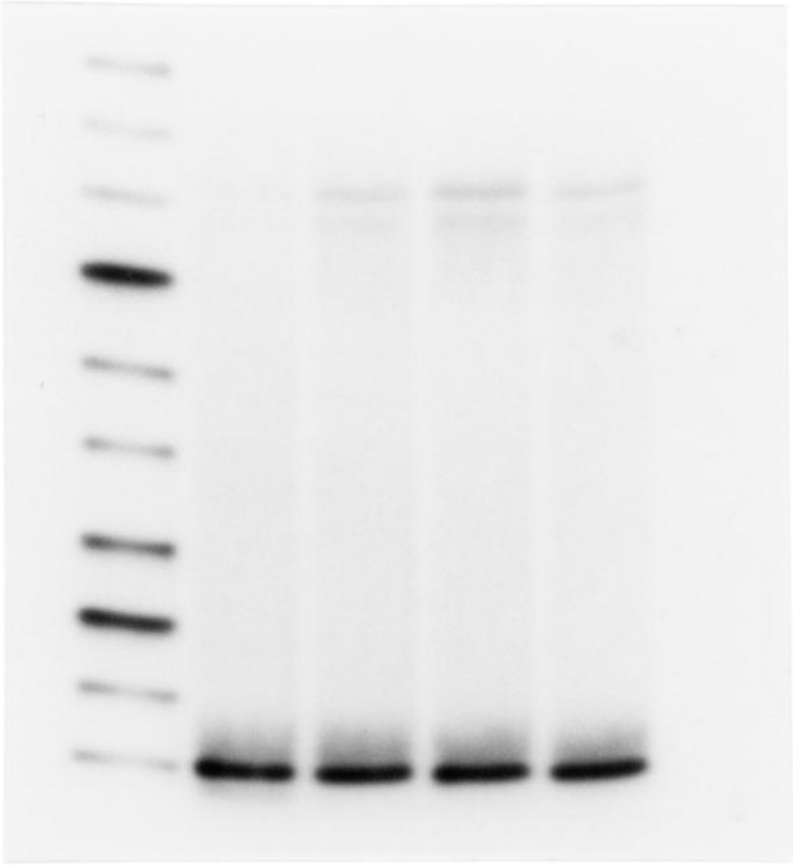

Fig 3I H3

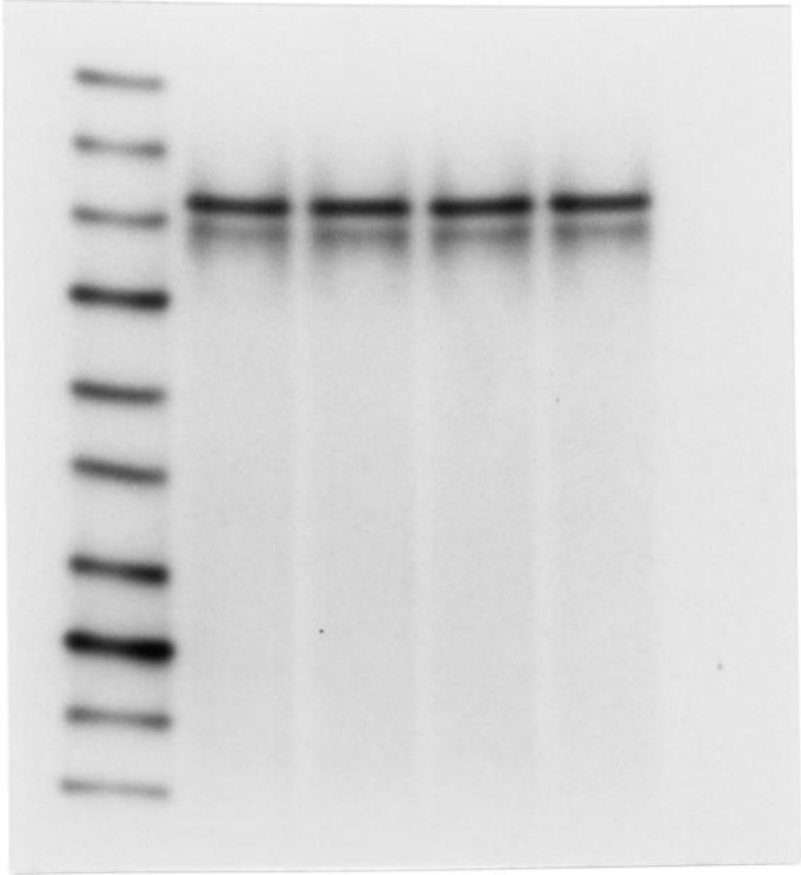

Fig 3I NRF2

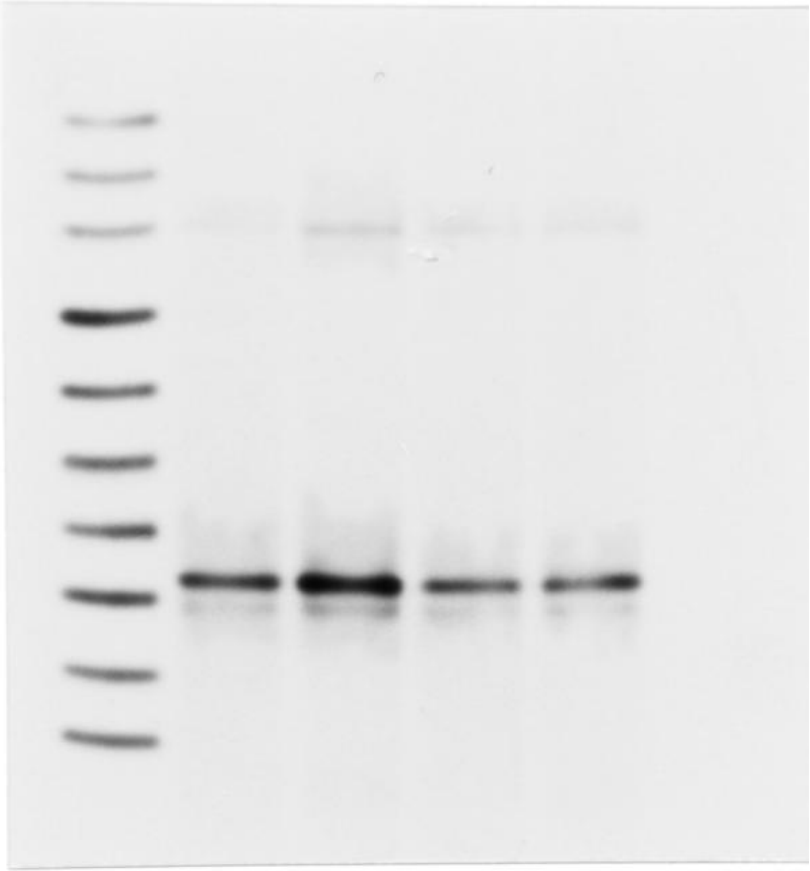

Fig 3I HO-1

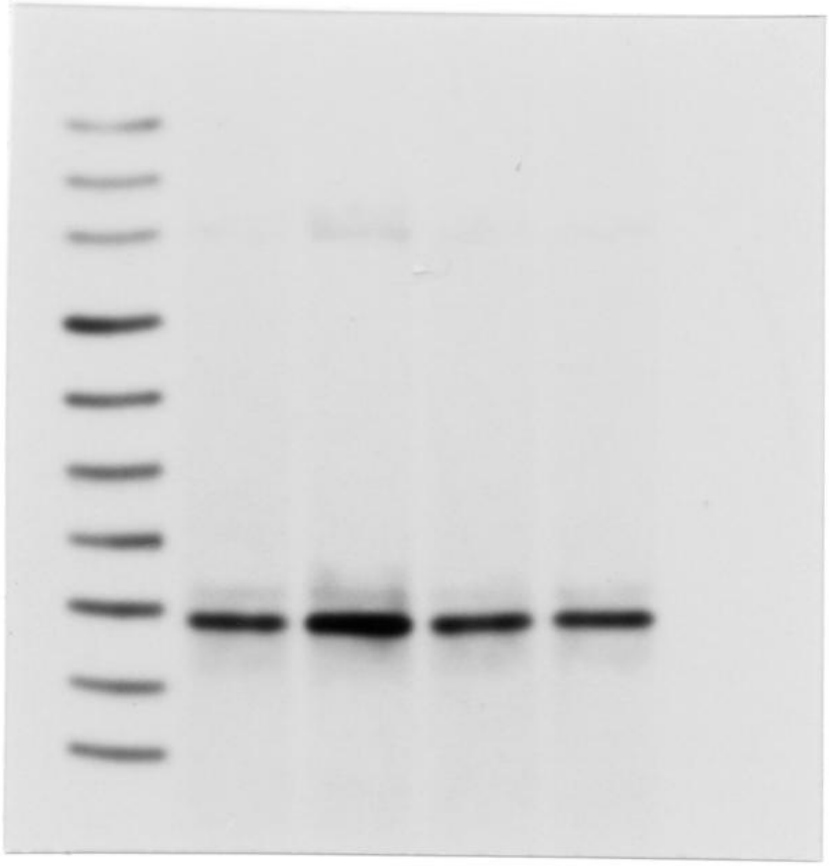

Fig 3I NQO-1

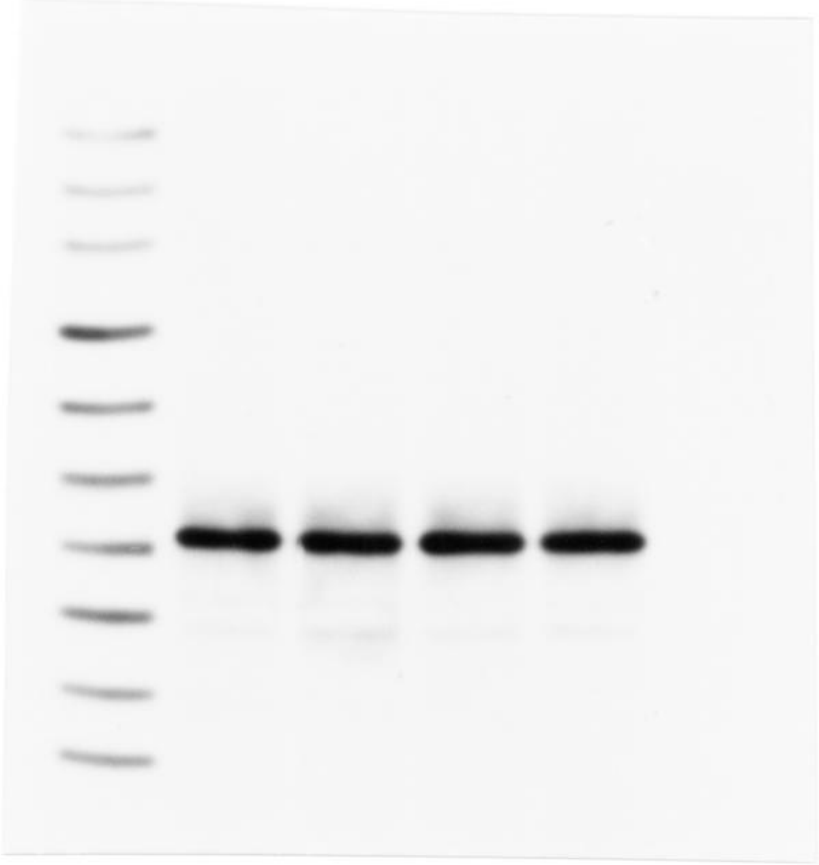

Fig 3I β-actin

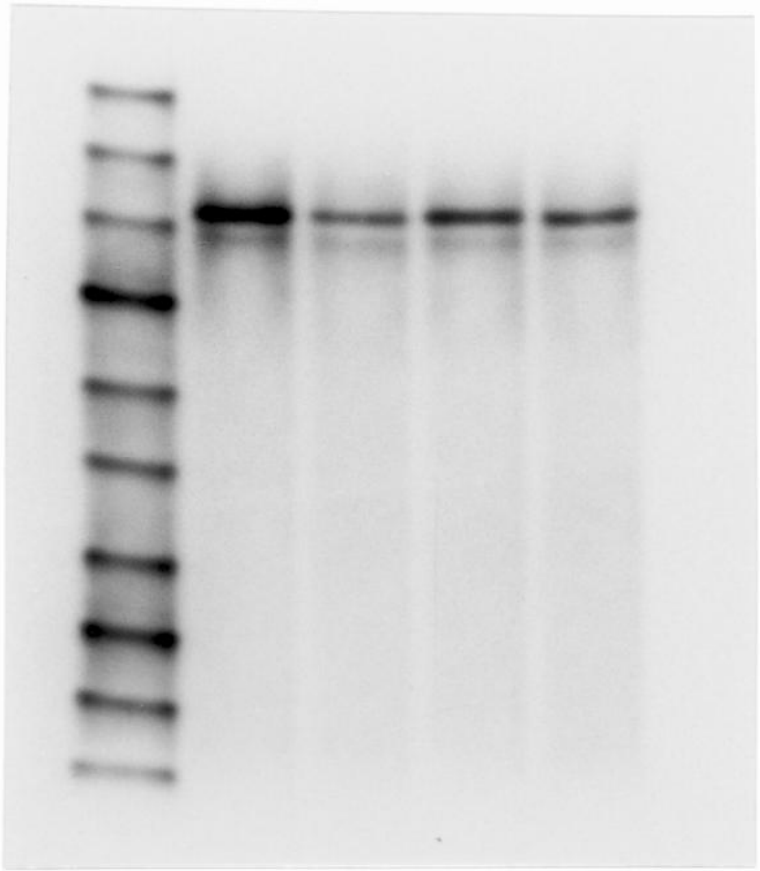

Fig 3J NLRP3

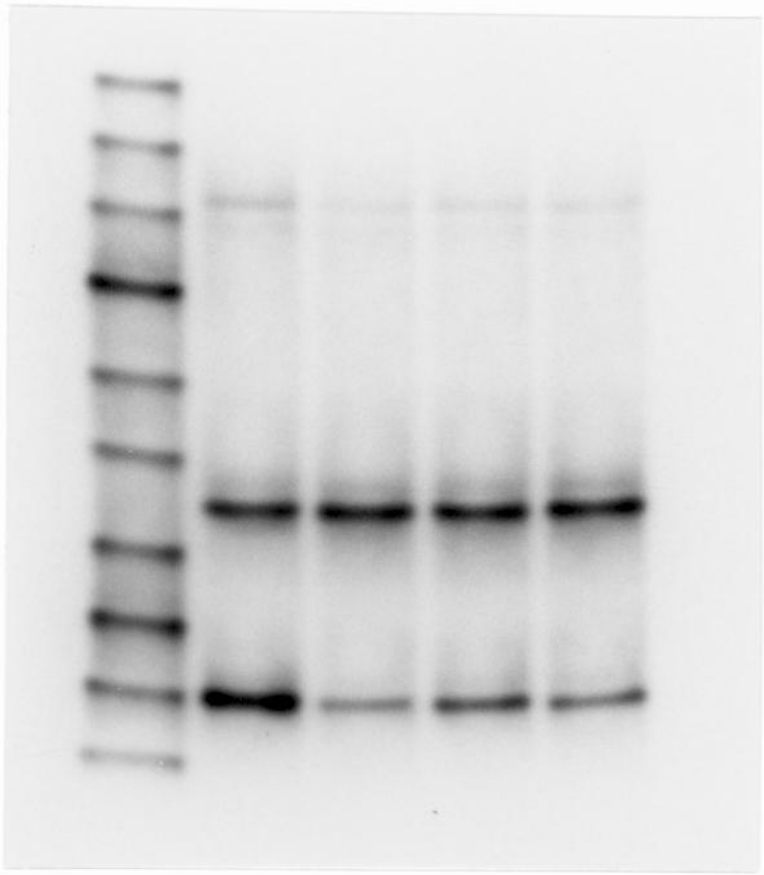

Fig 3J Caspase-1

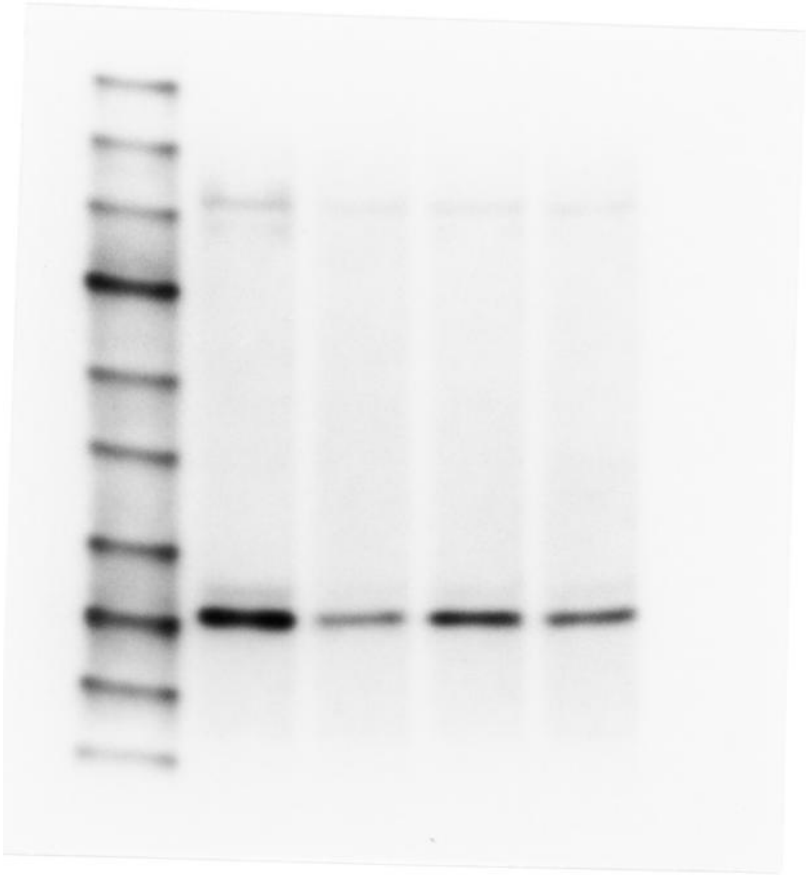

Fig 3J GSDMD-N

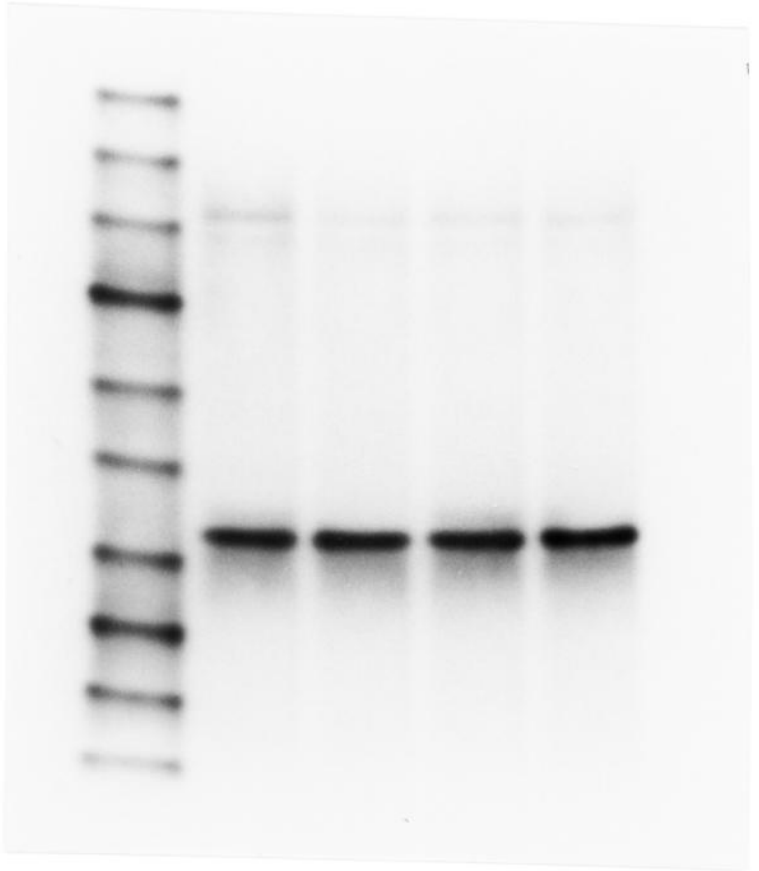

Fig 3J β-actin

Fig 4

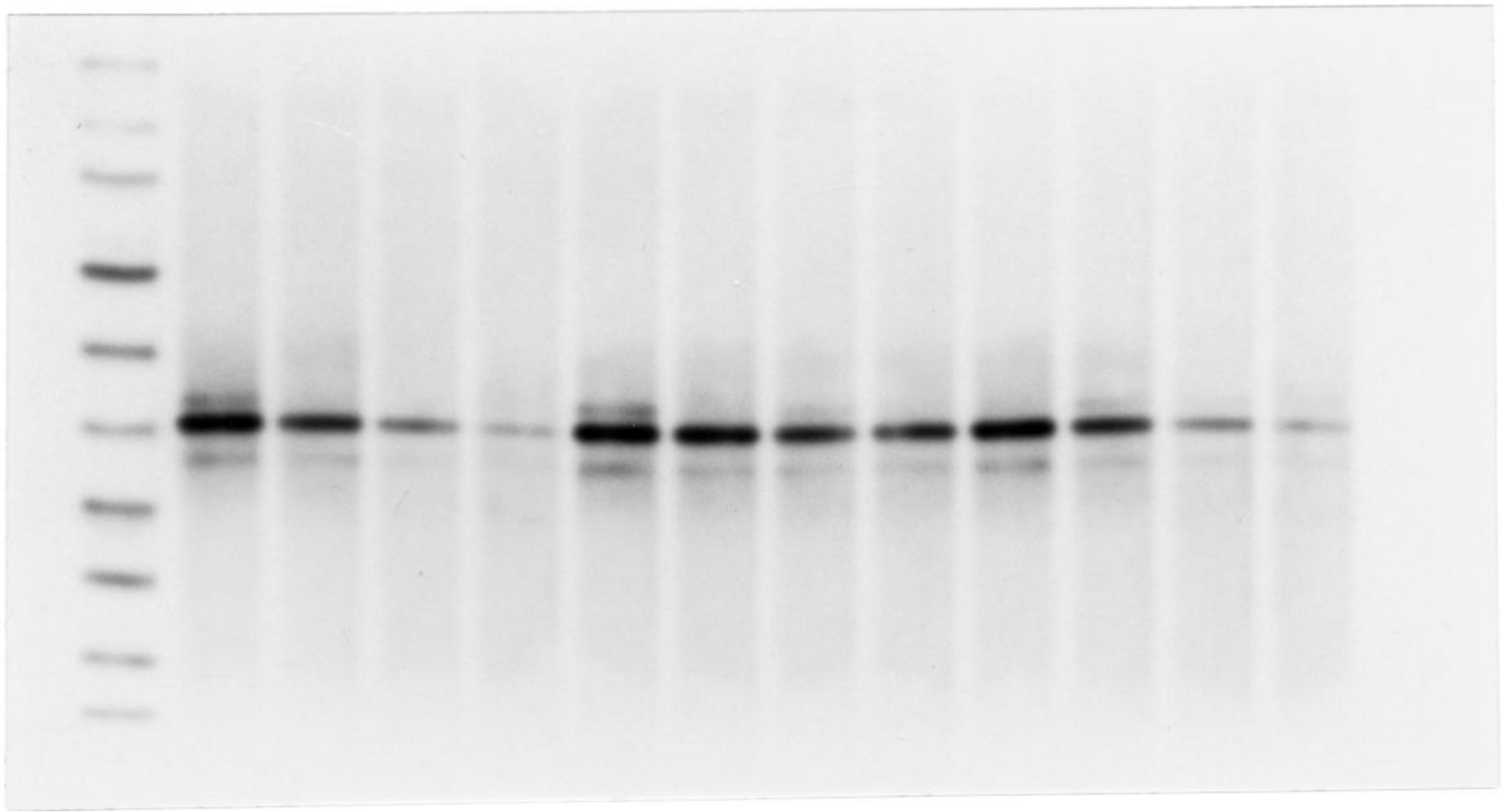

Fig 4A SELENOP

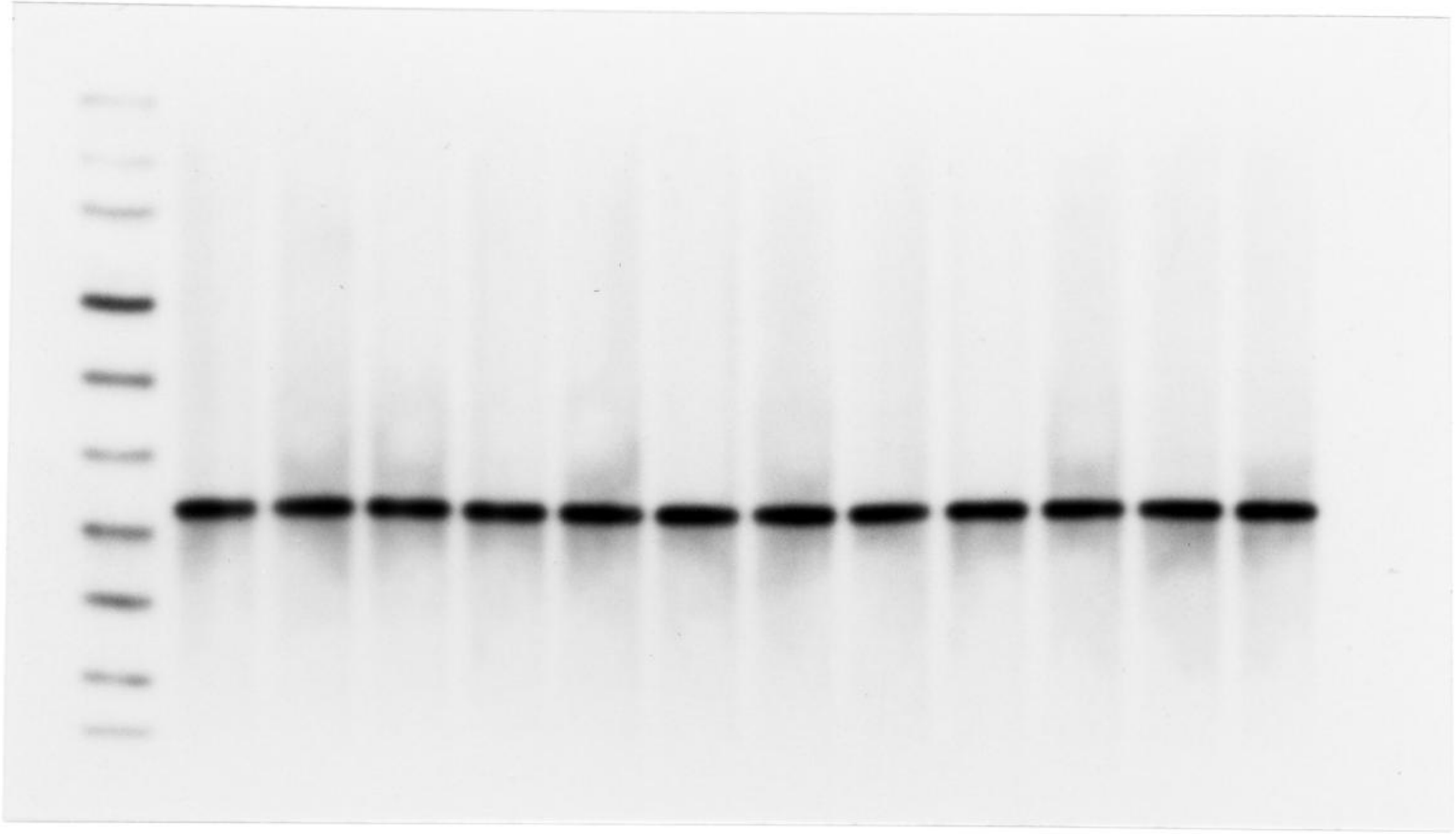

Fig 4A β-actin

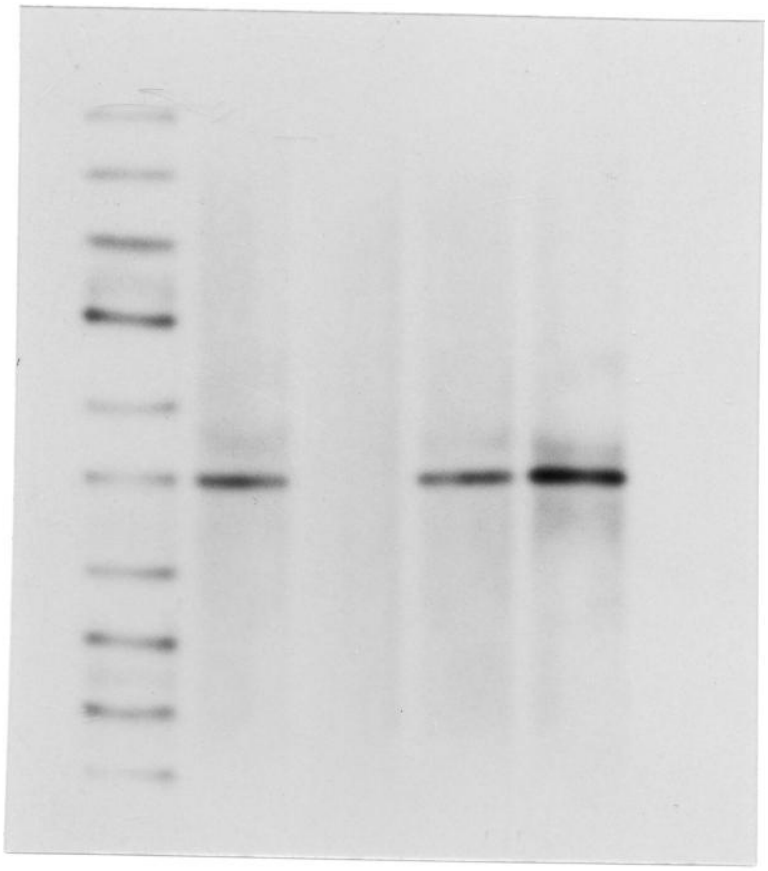

Fig 4C SELENOP

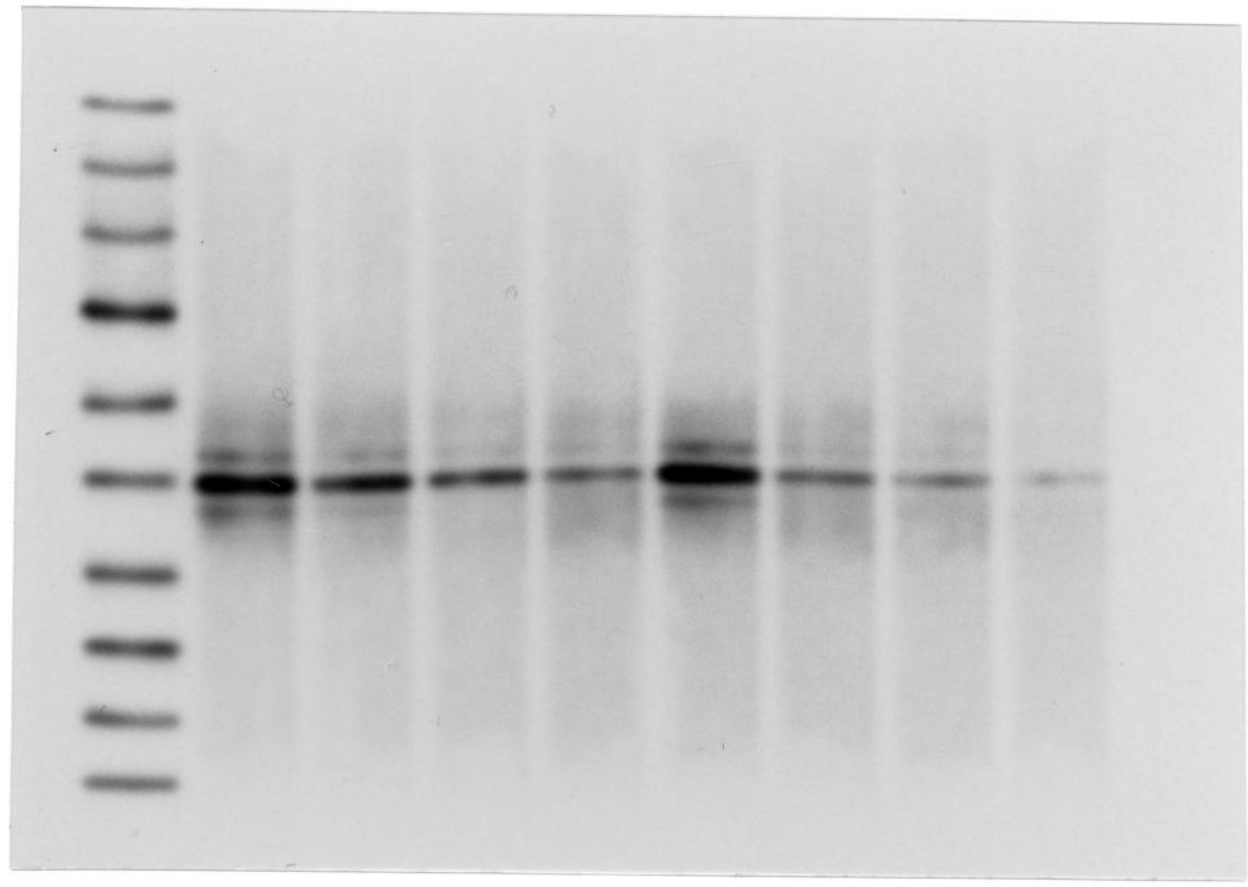

Fig 4B SELENOP

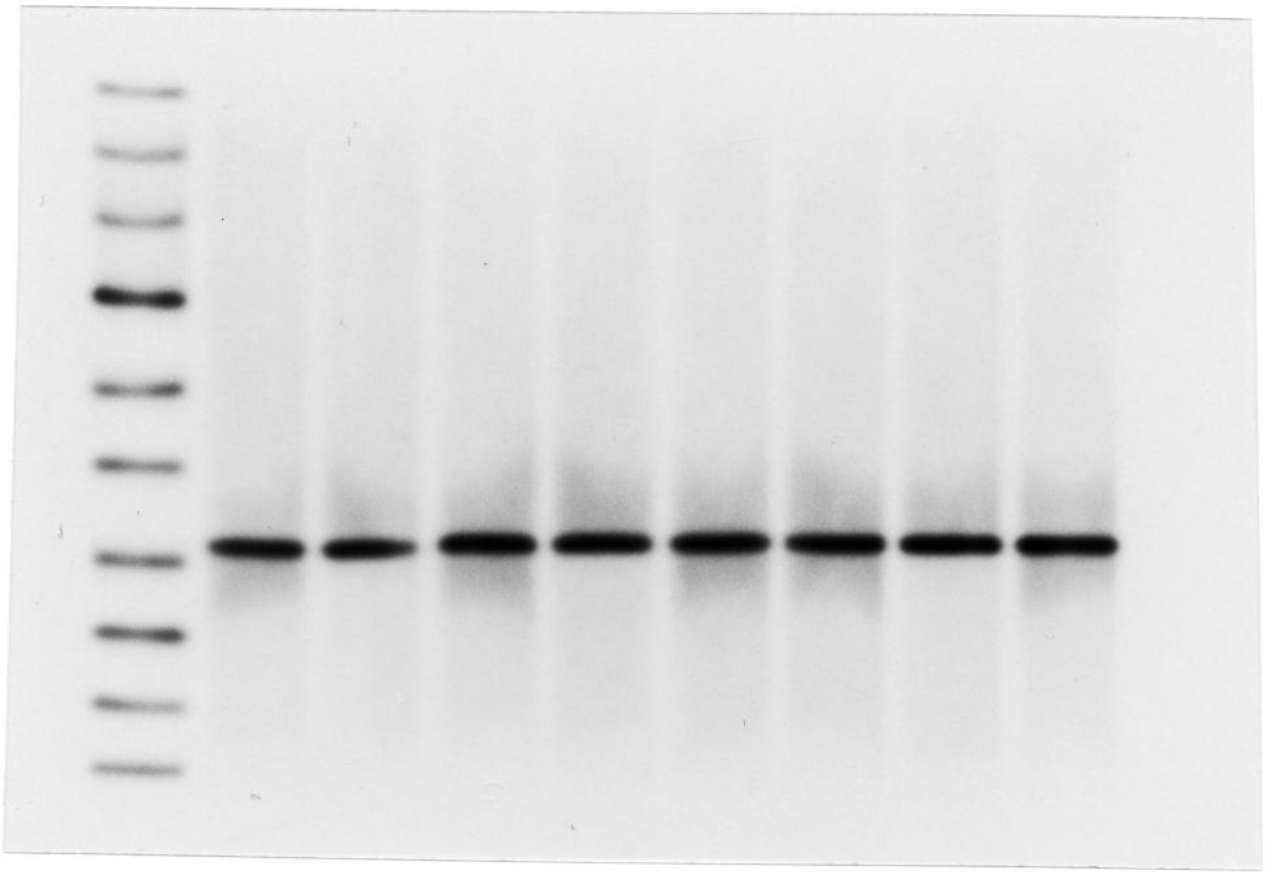

Fig 4B β-actin

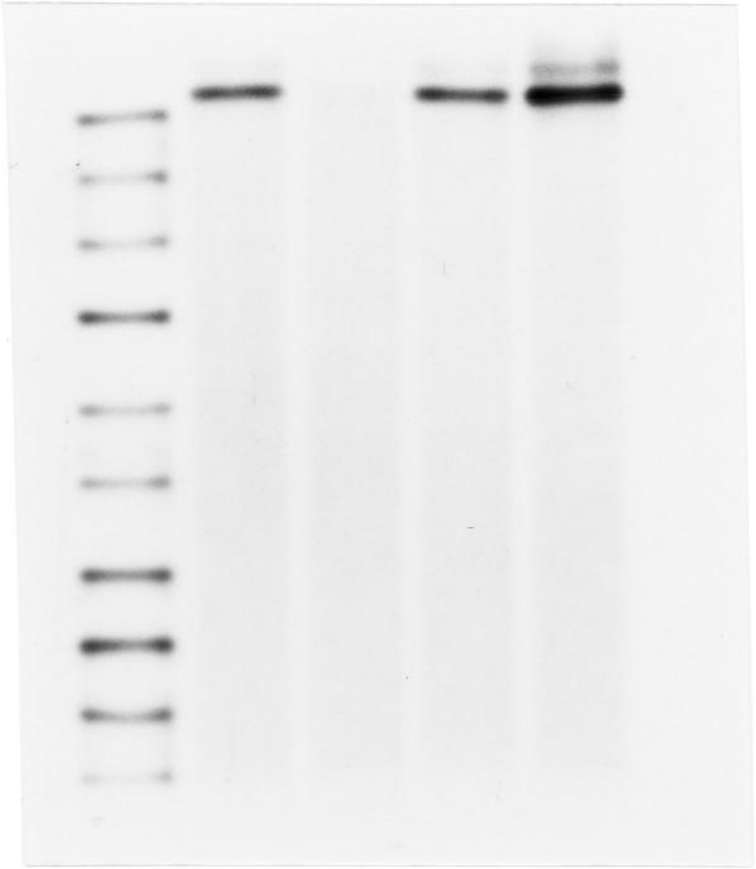

Fig 4C TRIP12

Fig 4

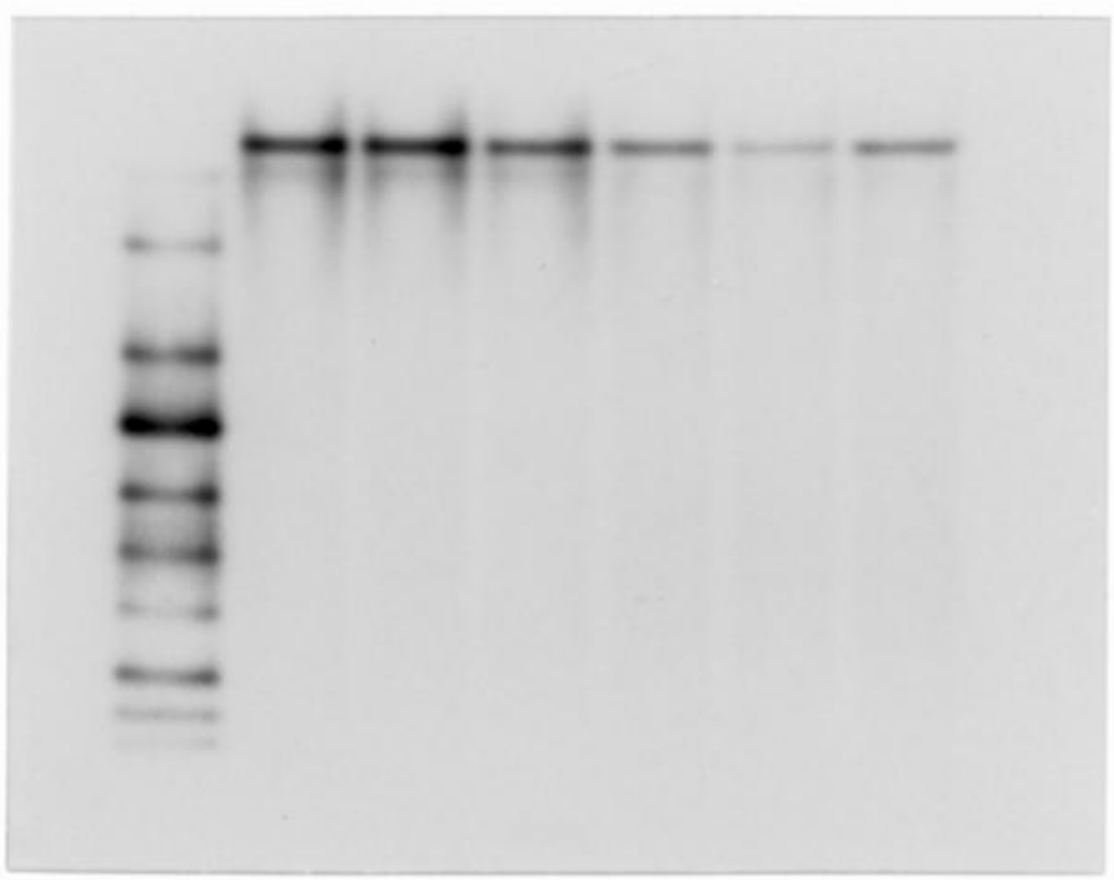

Fig 4E TRIP12
(Nuclear)

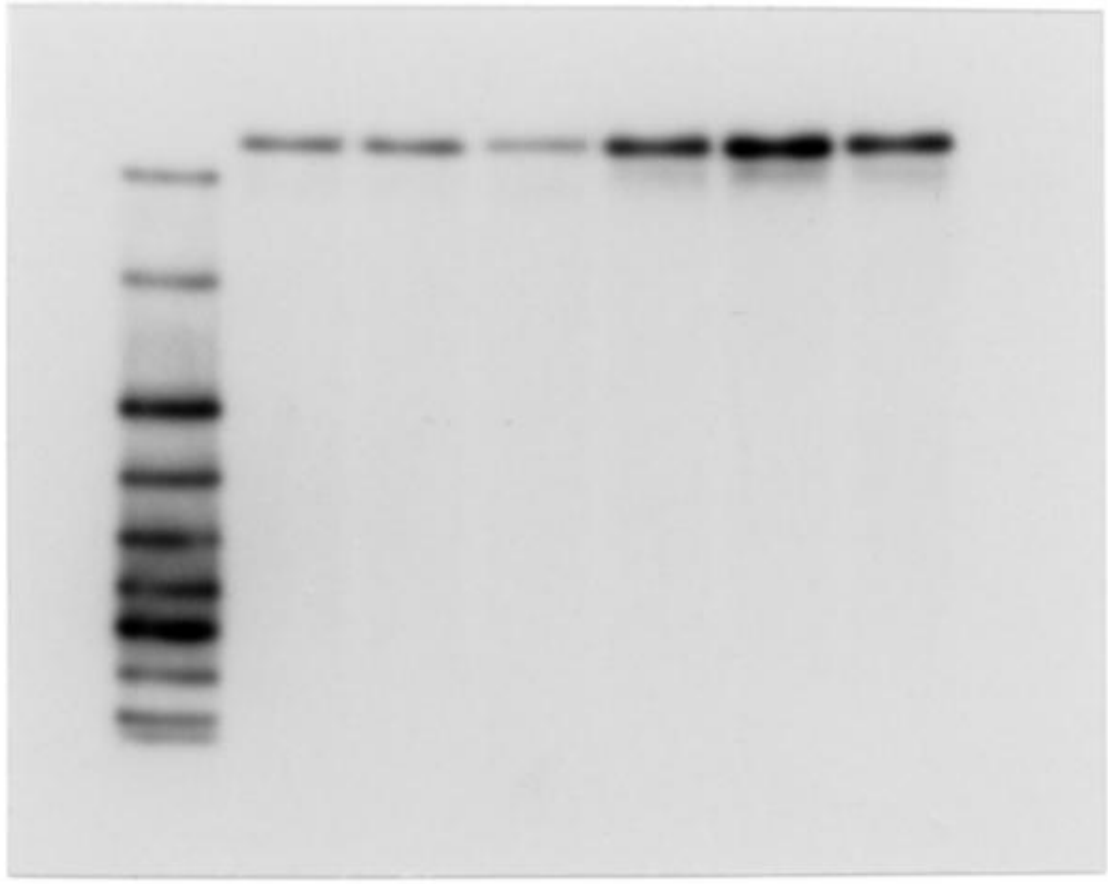

Fig 4E TRIP12
(Cytoplasm)

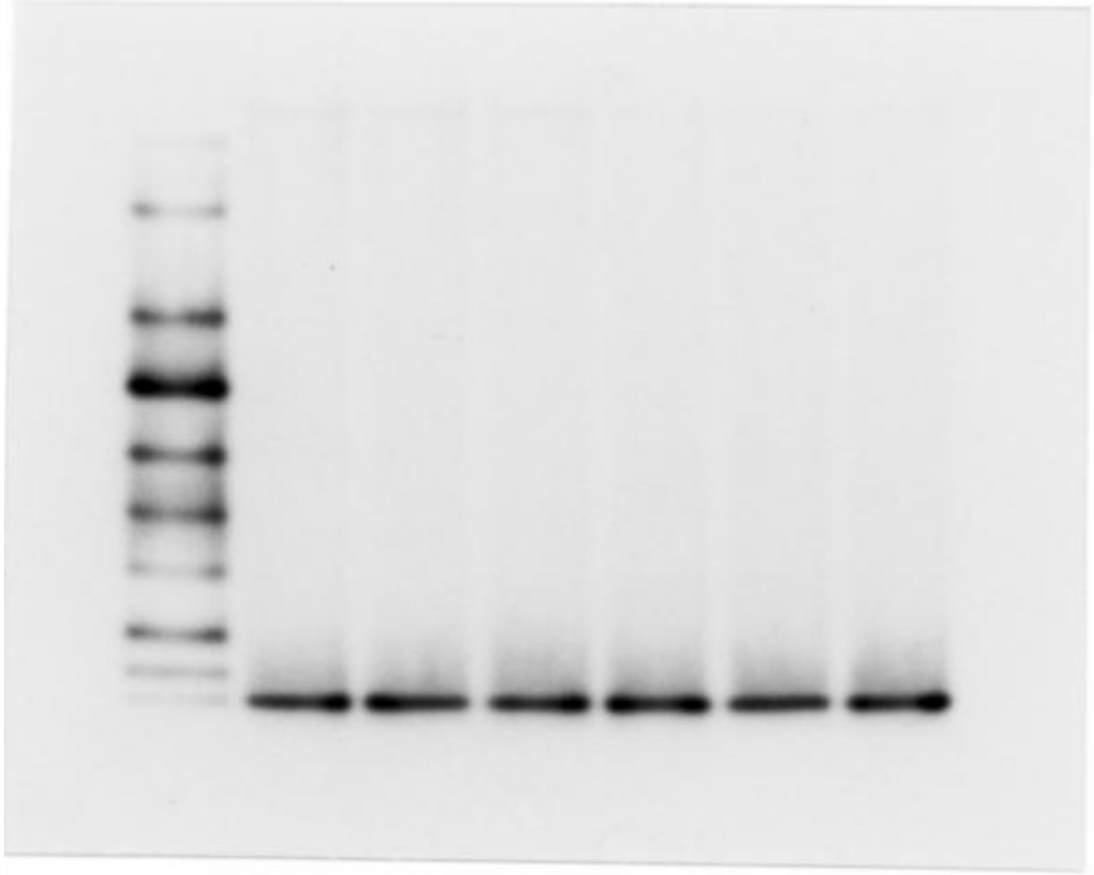

Fig 4E H3
(Nuclear)

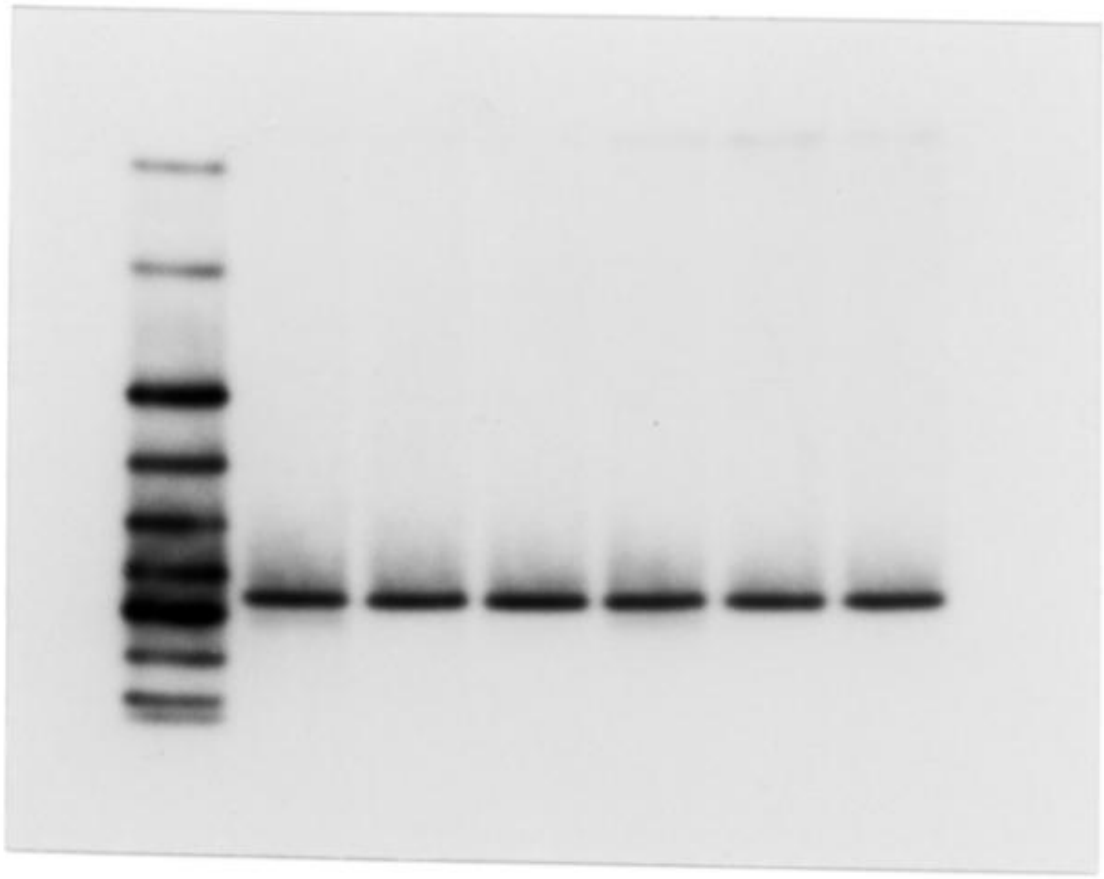

Fig 4E β-actin
(Cytoplasm)

Fig 4

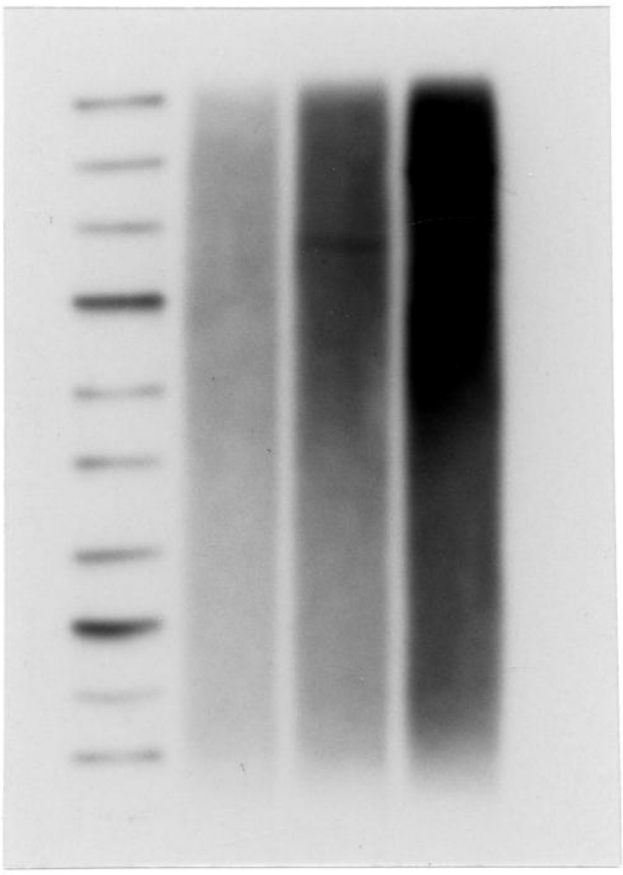

Fig 4I Ub

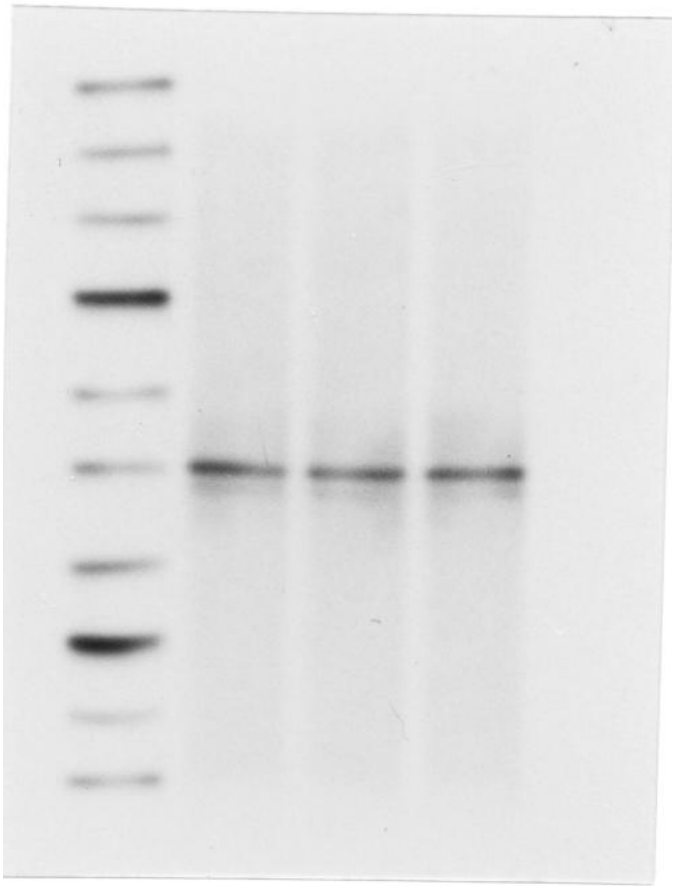

Fig 4I SELENOP

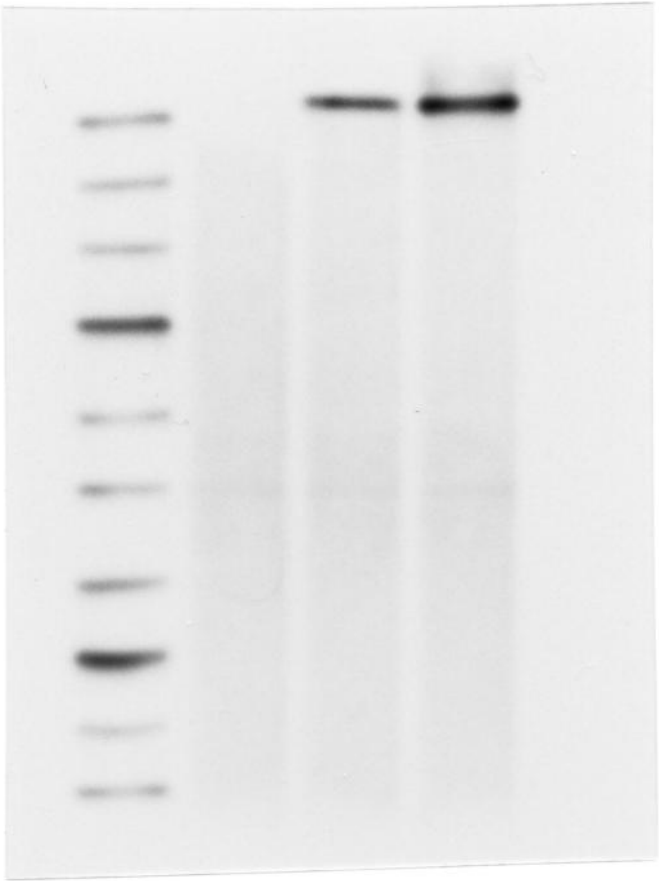

Fig 4I TRIP12

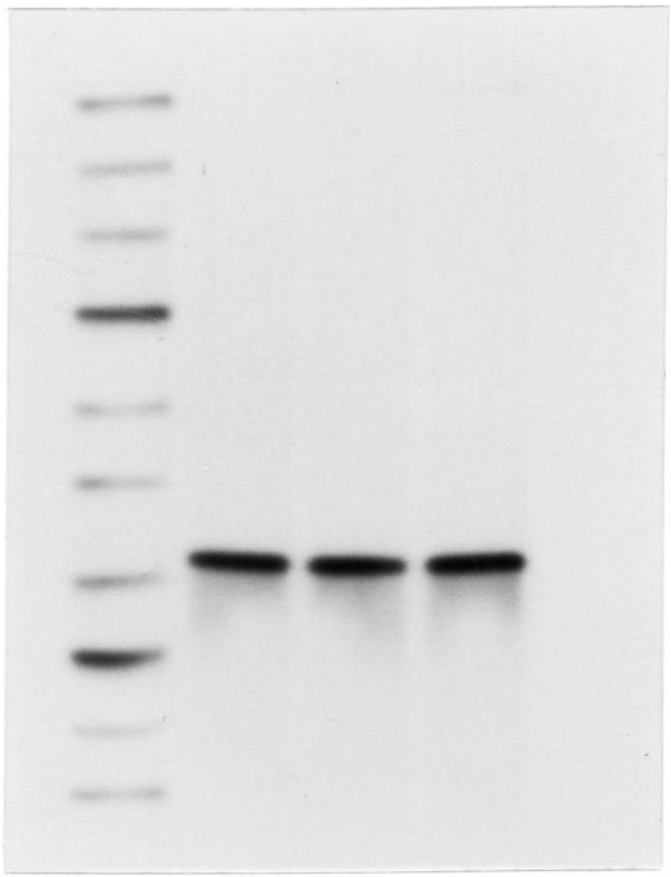

Fig 4I β-actin

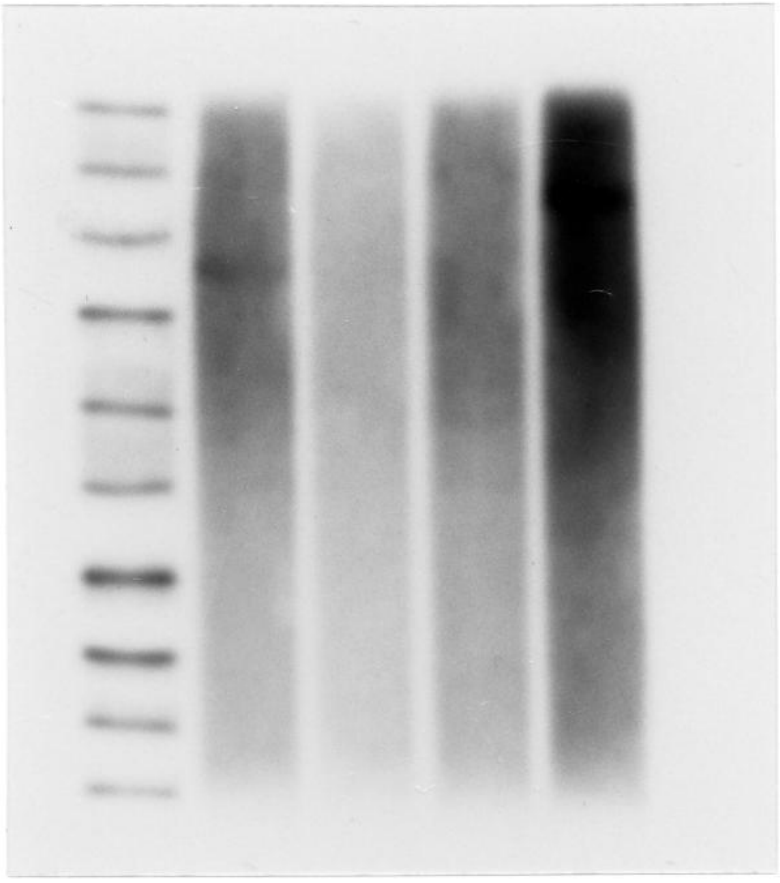

Fig 4J Ub

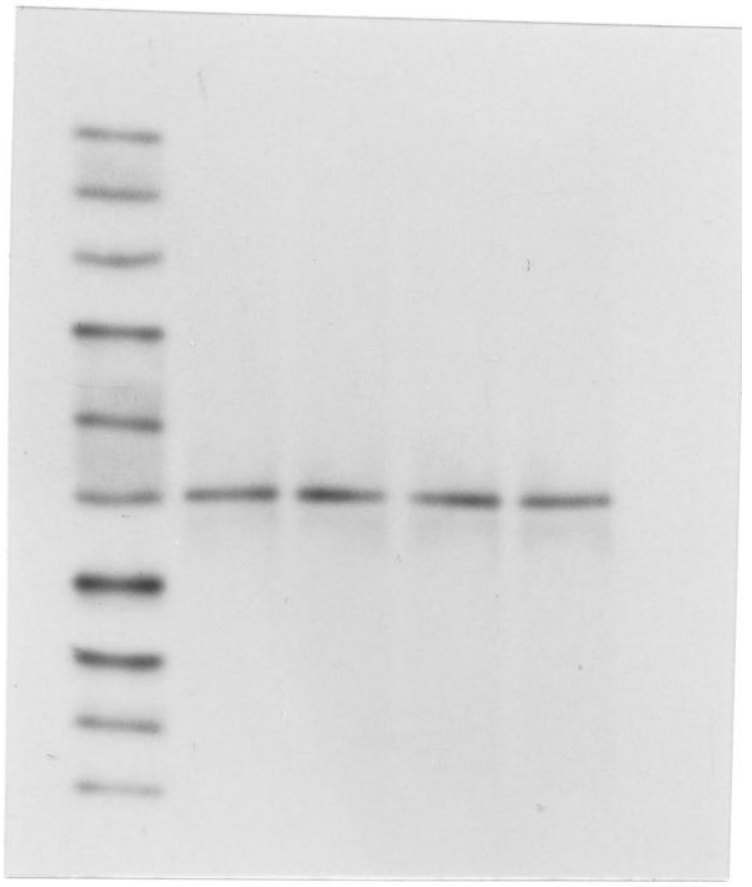

Fig 4J SELENOP

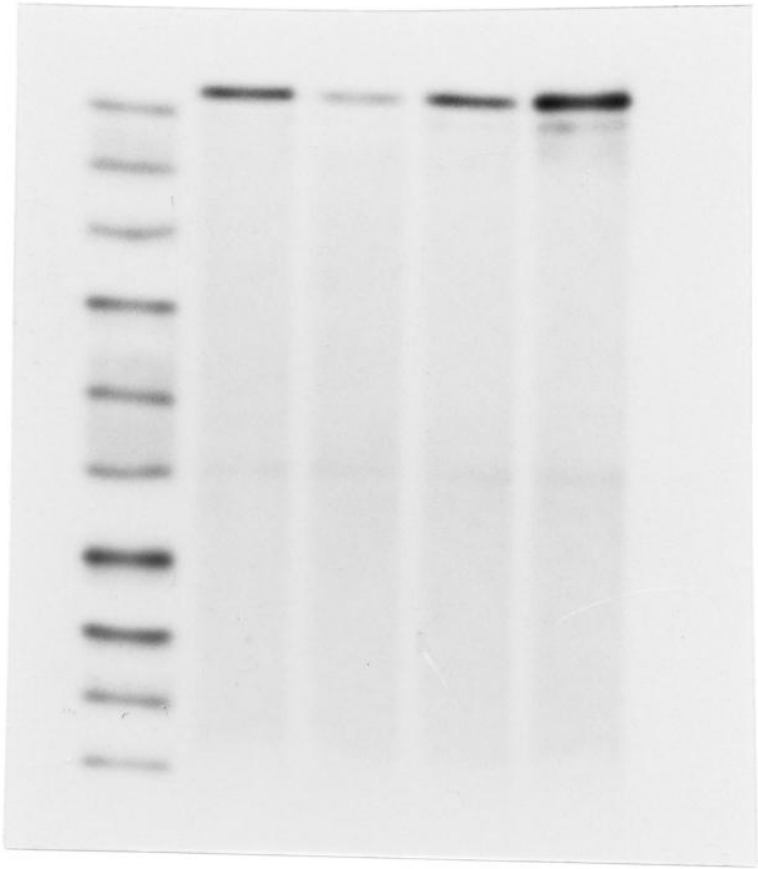

Fig 4J TRIP12

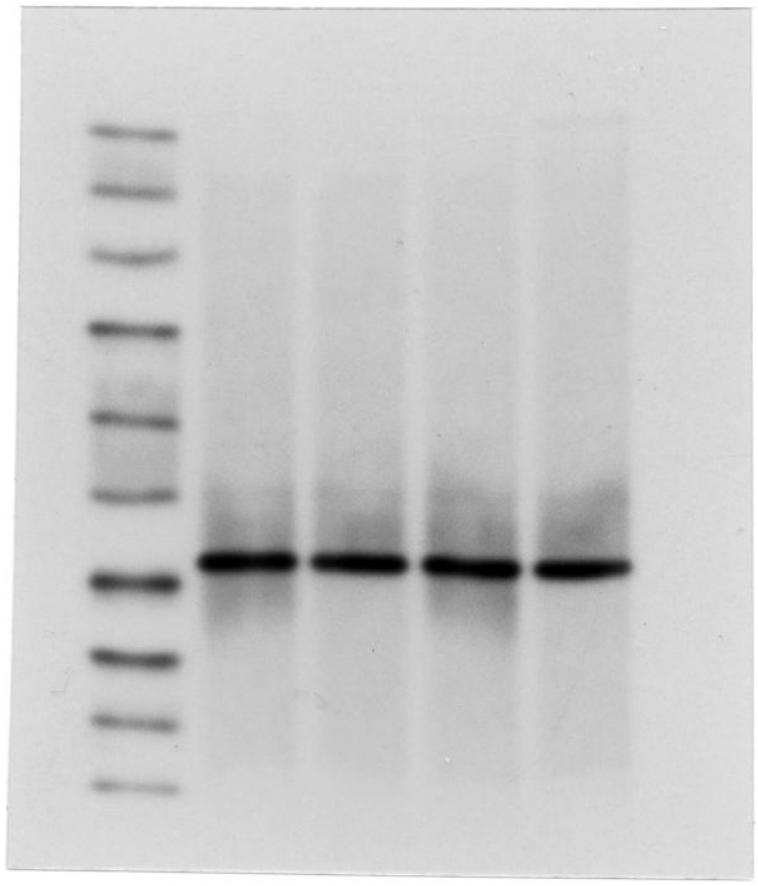

Fig 4J β-actin

Fig 4

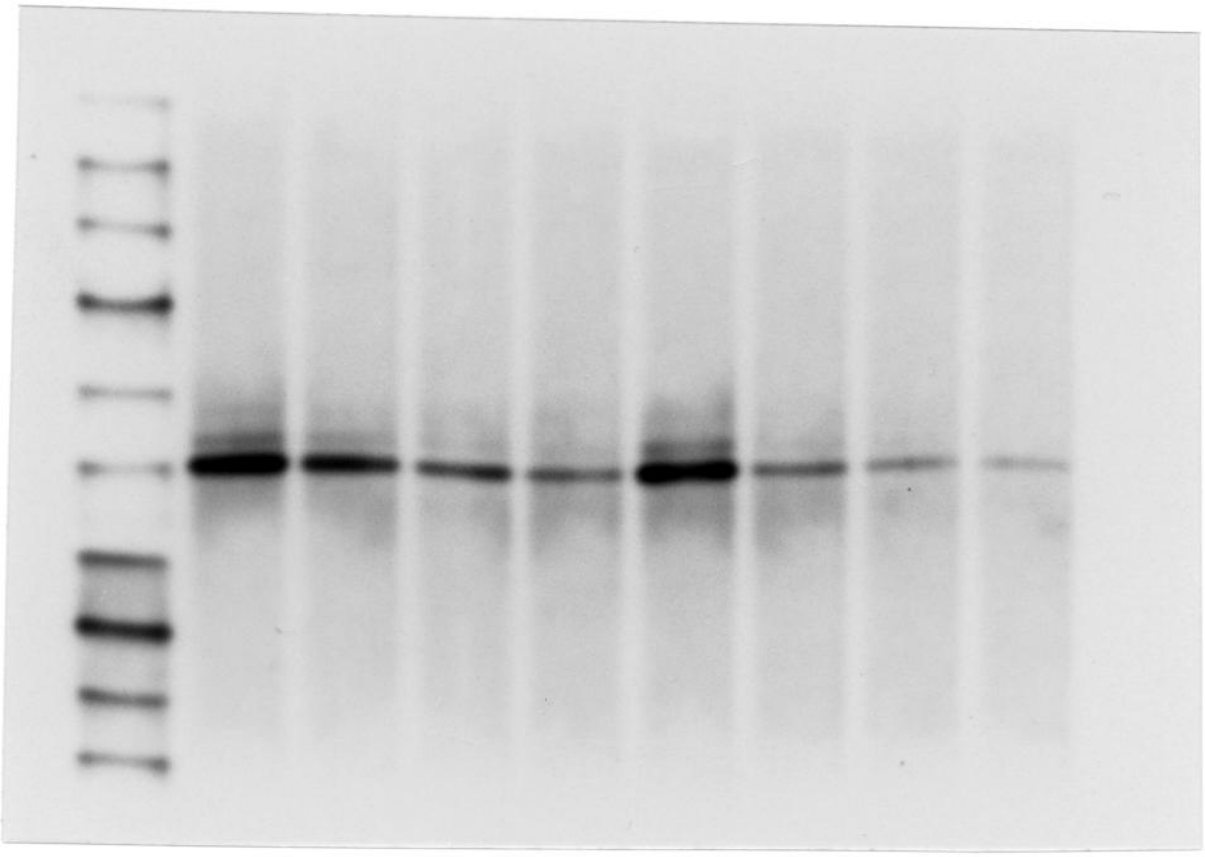

Fig 4K SELENOP

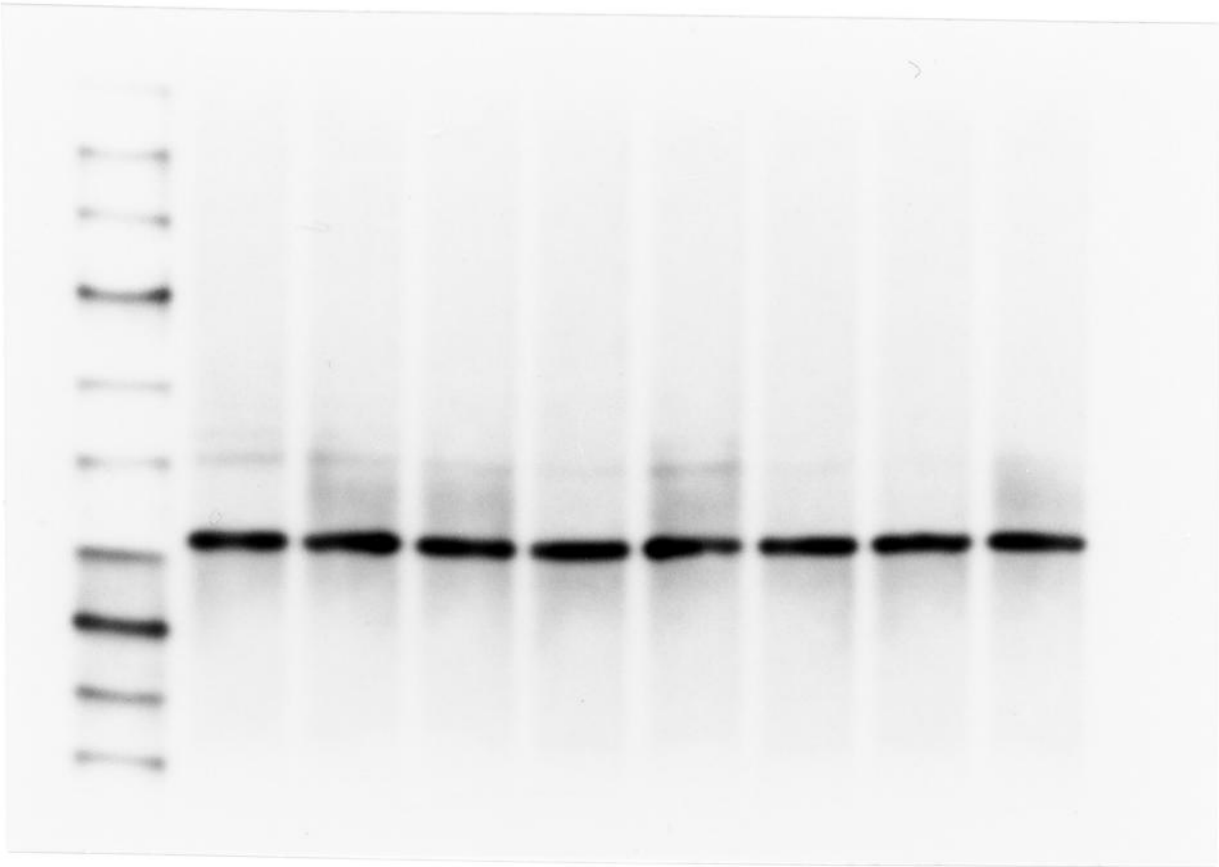

Fig 4K β-actin

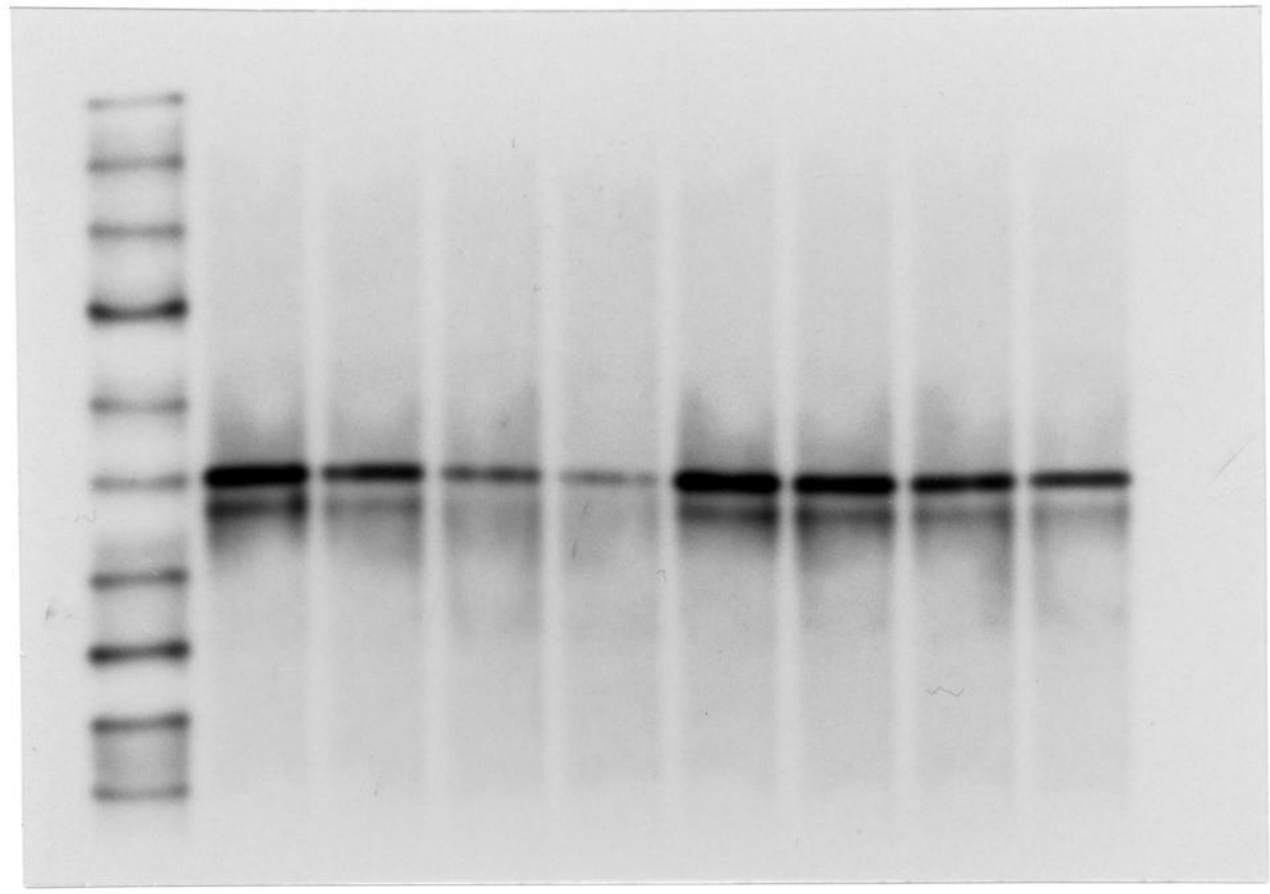

Fig 4L SELENOP

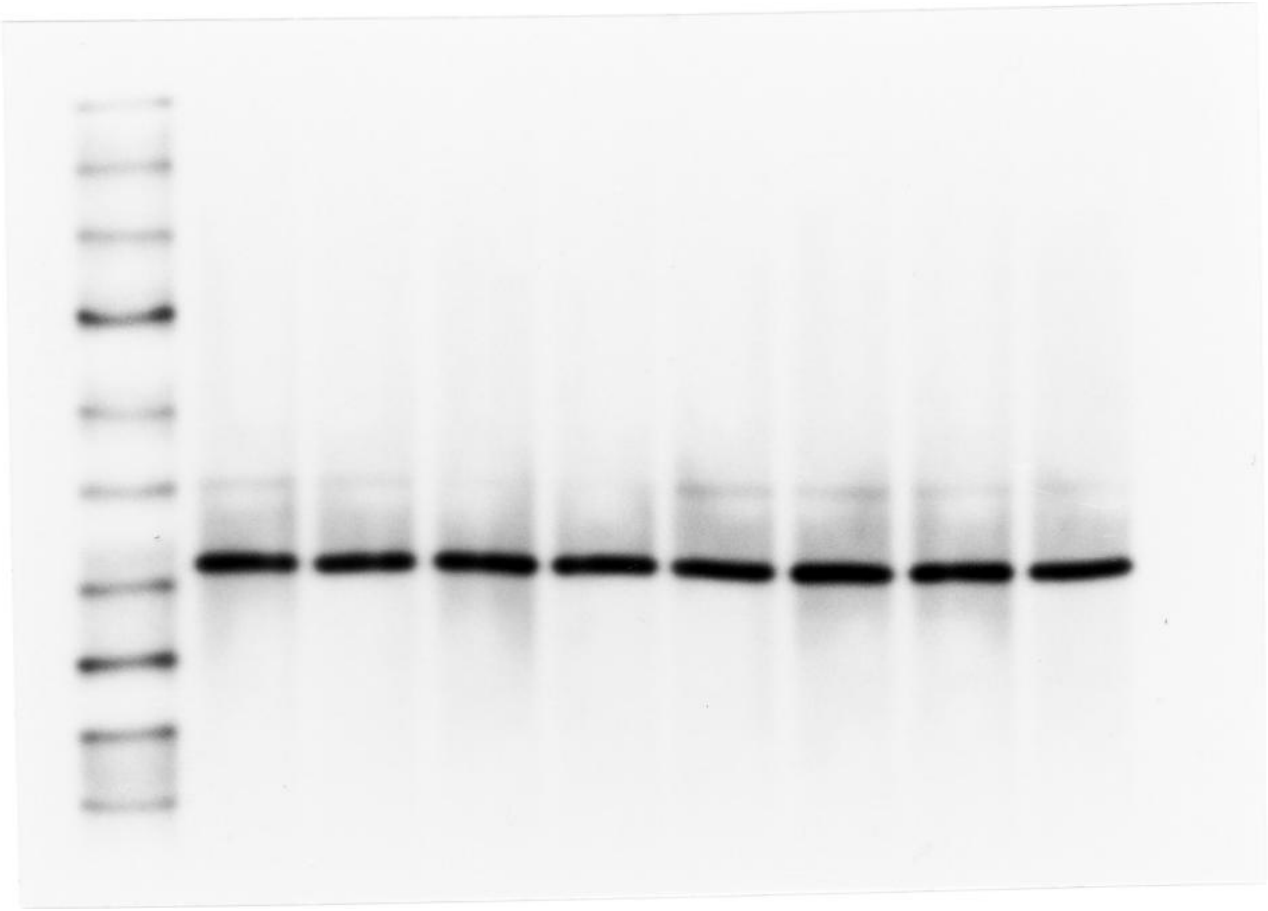

Fig 4L β-actin

Fig 5

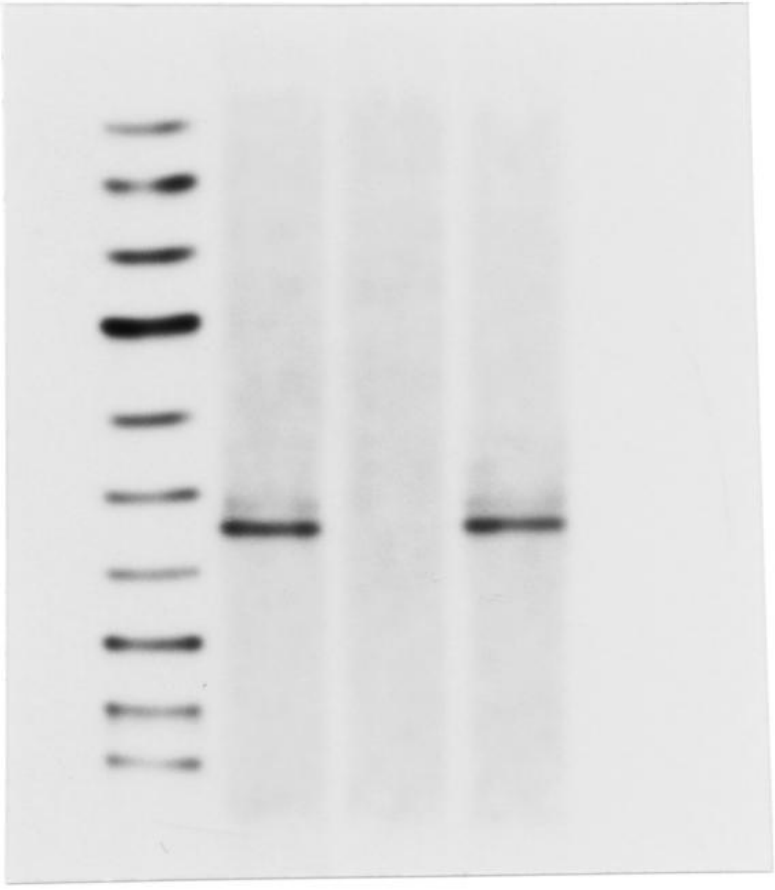

Fig 5H CREB1

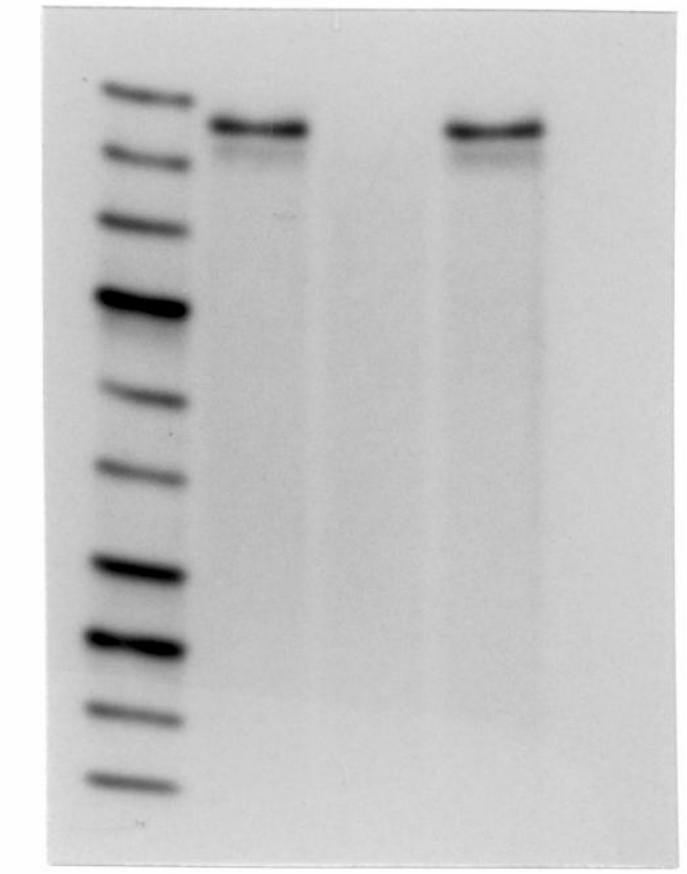

Fig 5H SMARCA4

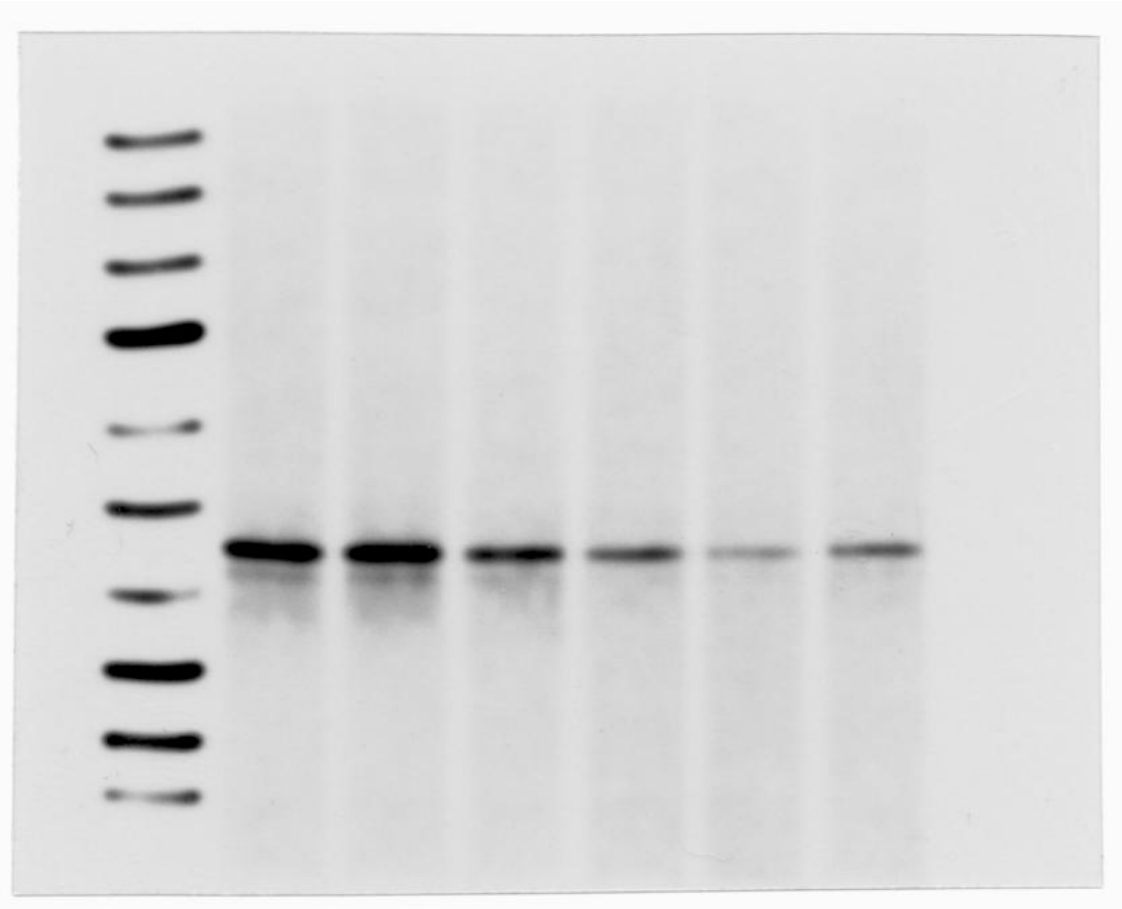

Fig 5K CREB1

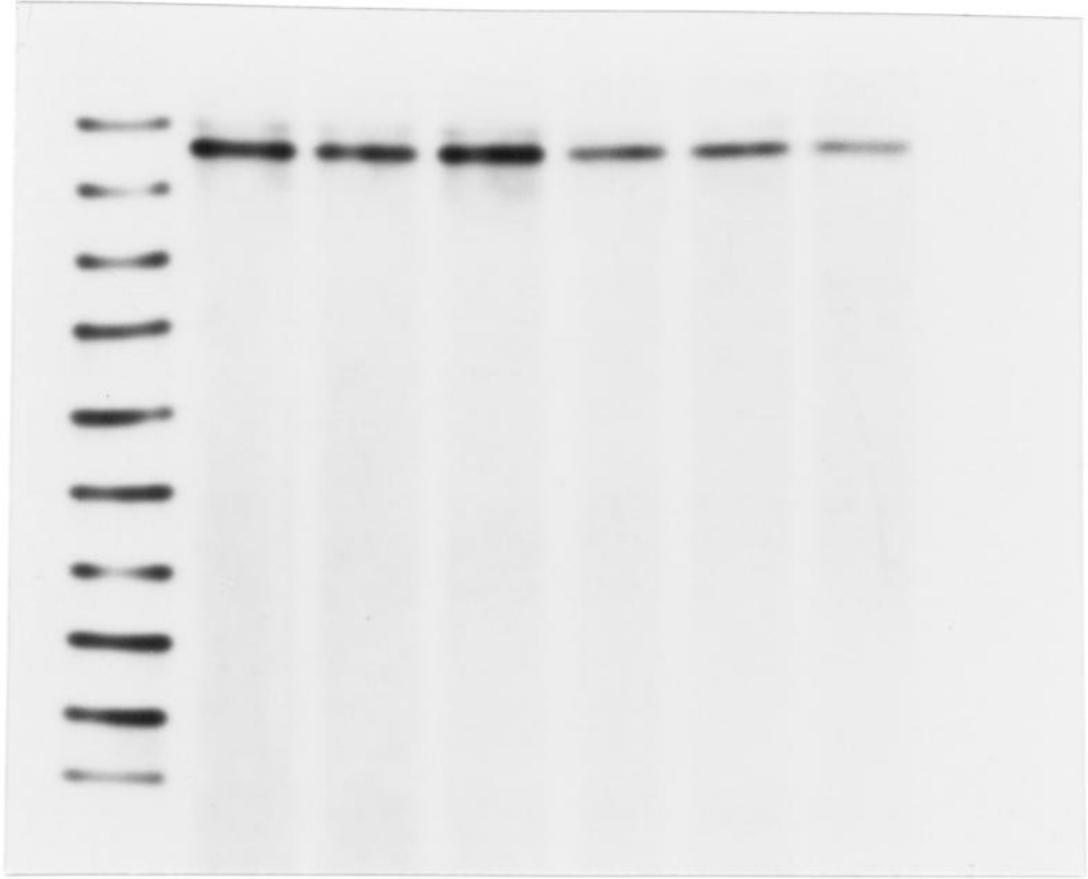

Fig 5K SMARCA4

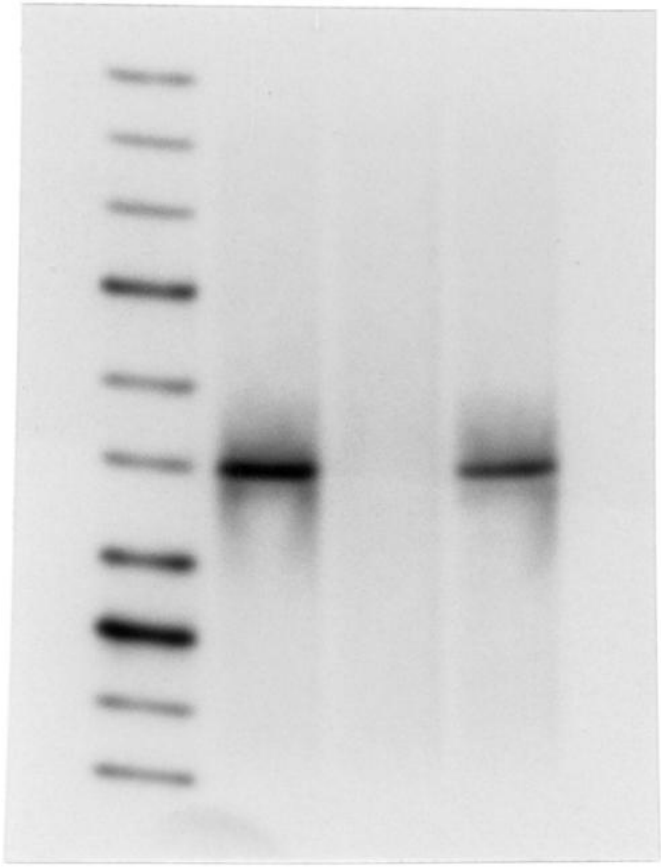

Fig 5L SELENOP

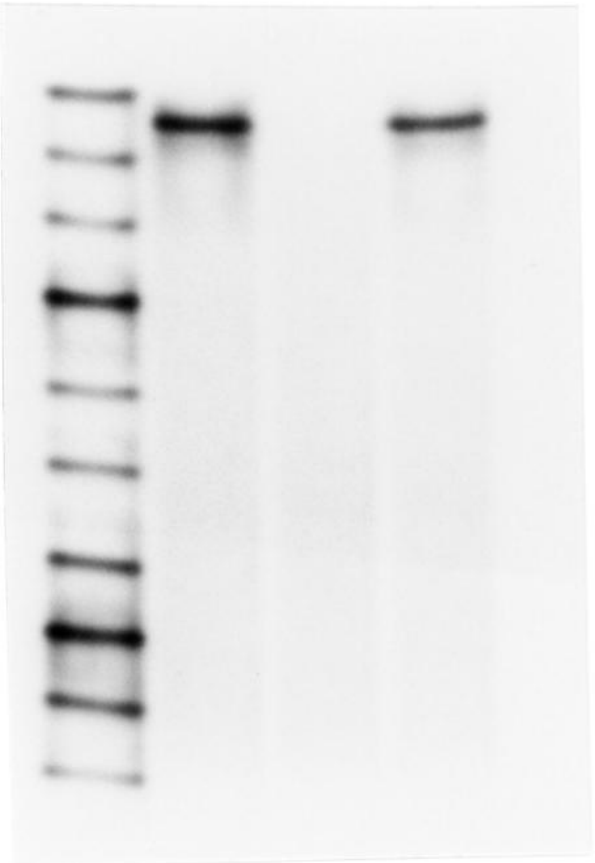

Fig 5L SMARCA4

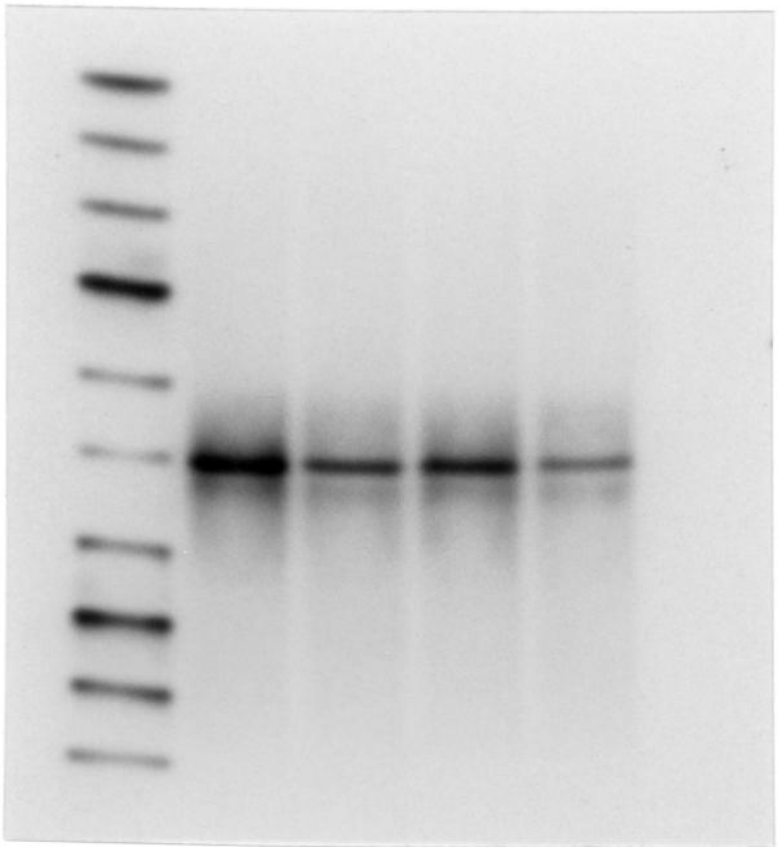

Fig 5S SELENOP

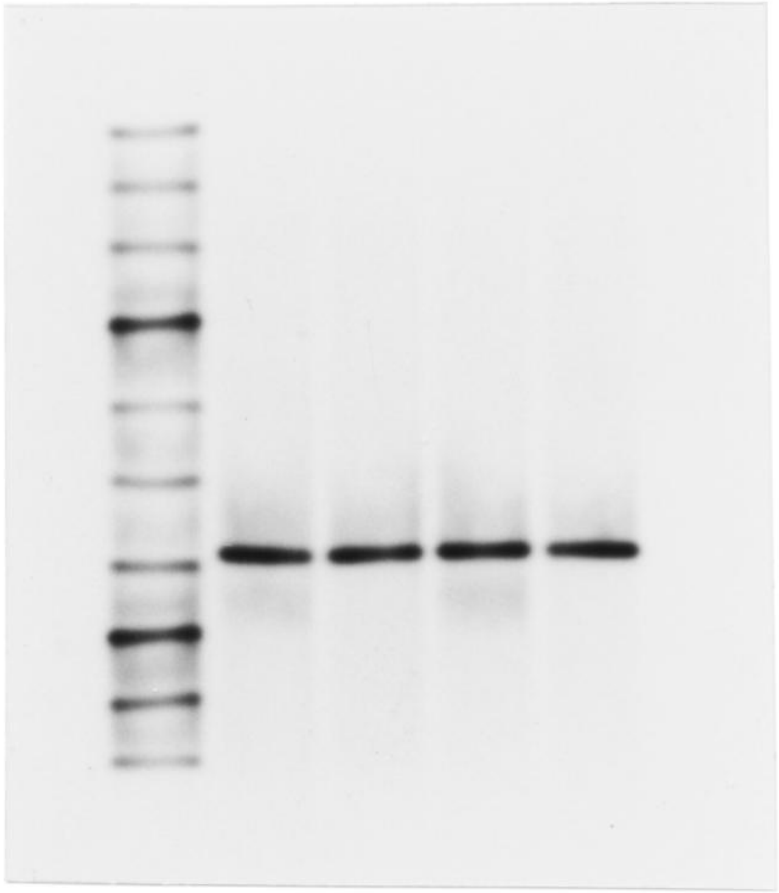

Fig 5S β-actin

Fig 5

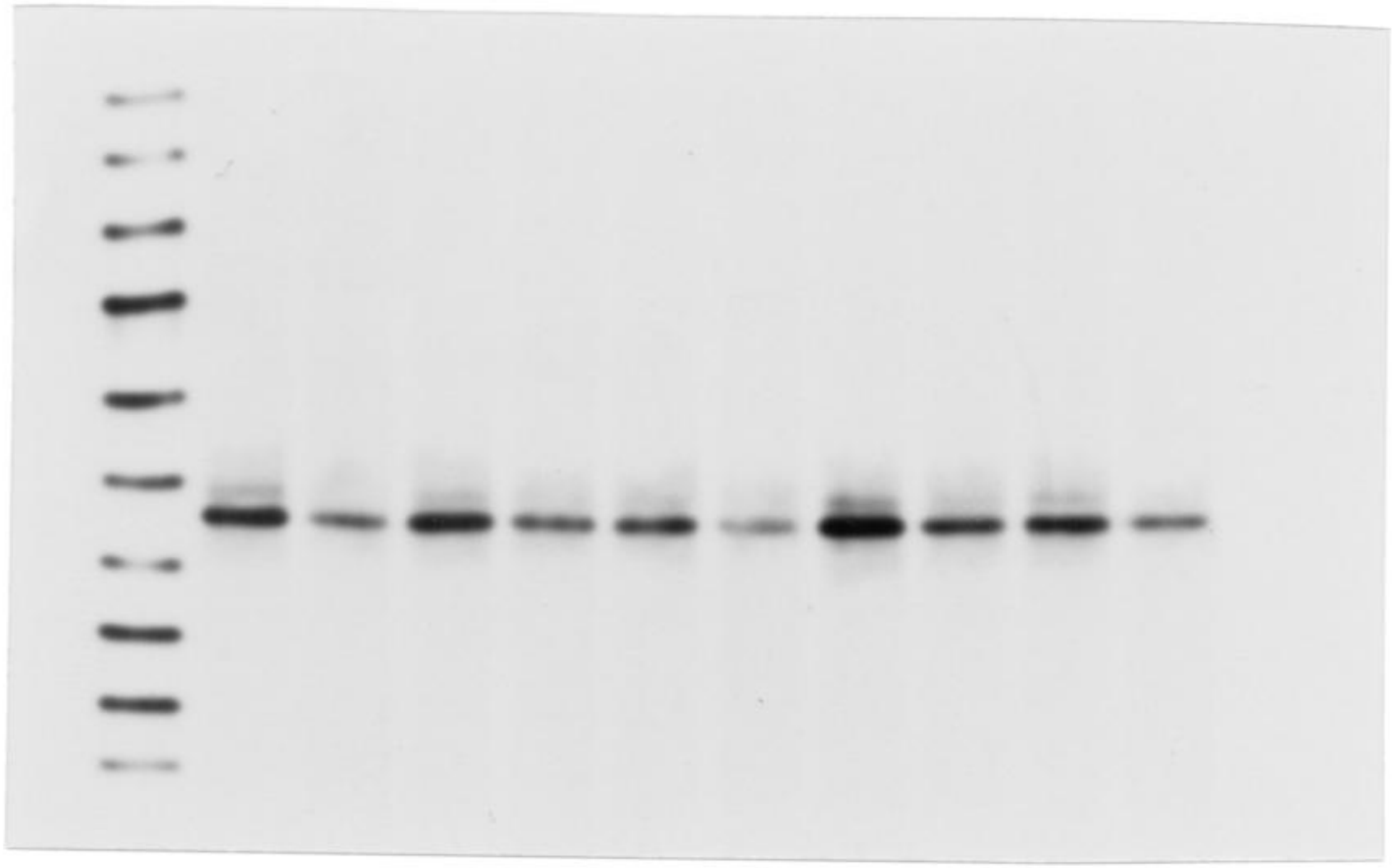

Fig 5G p-CREB1

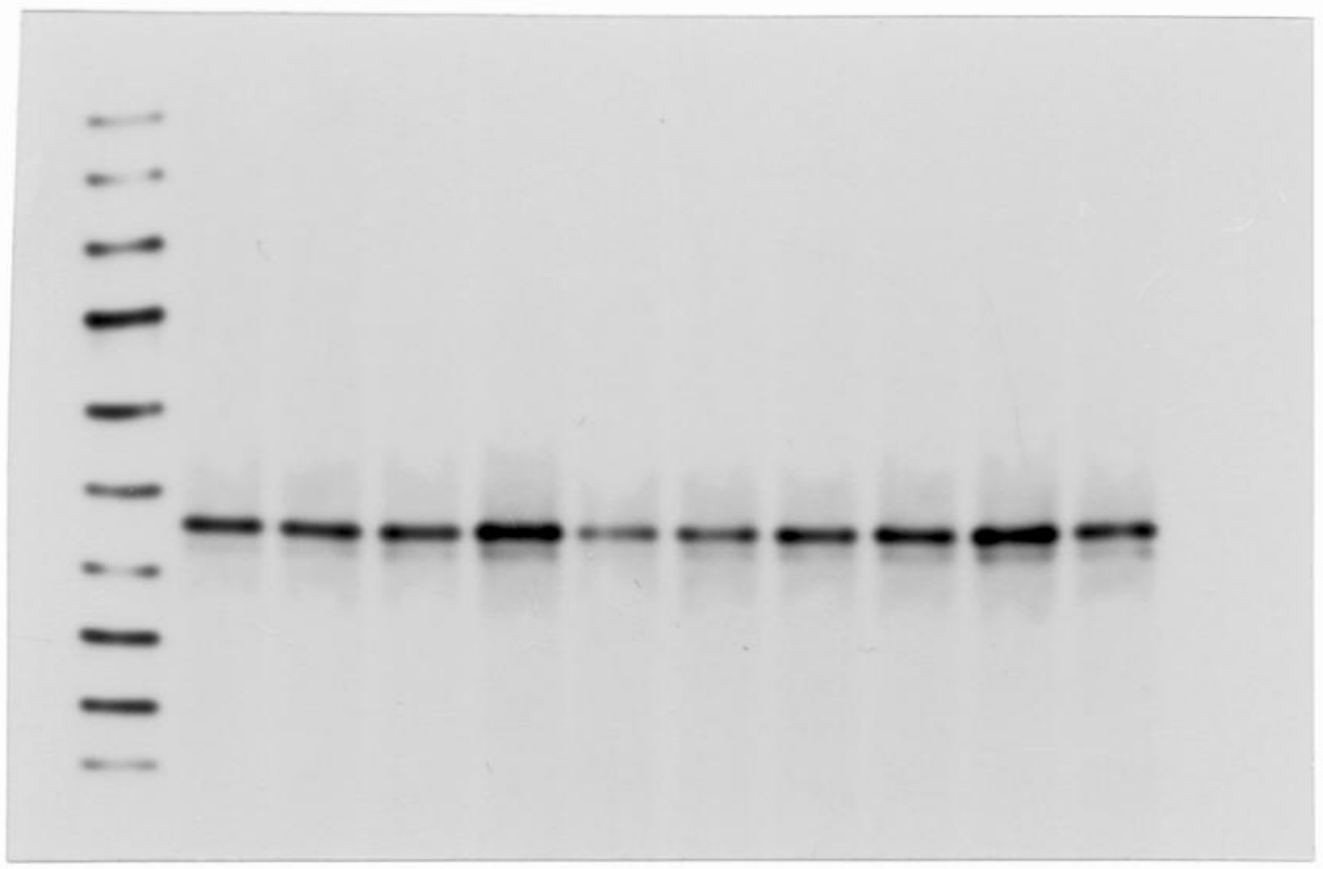

Fig 5G CREB1

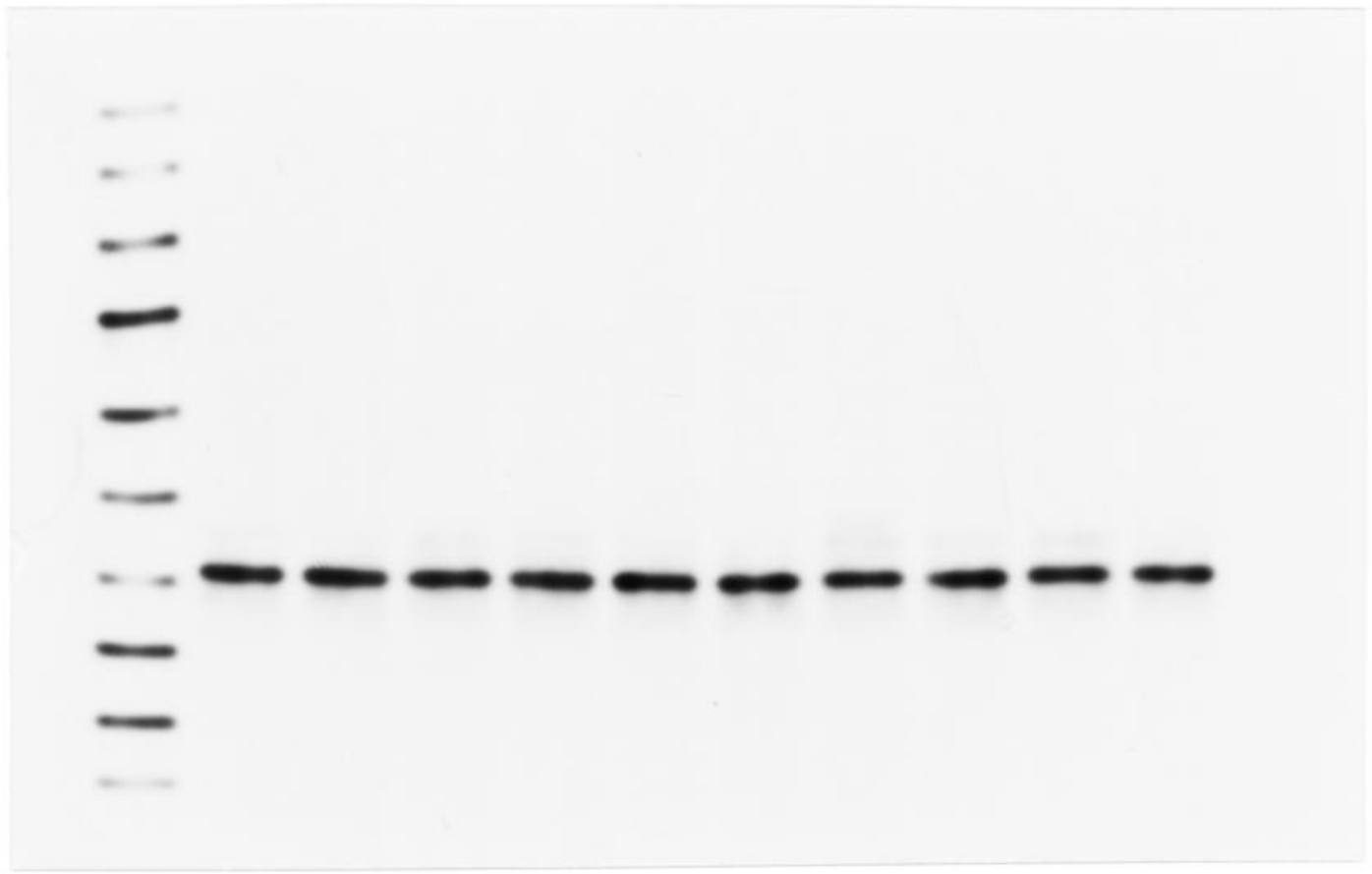

Fig 5G β-actin

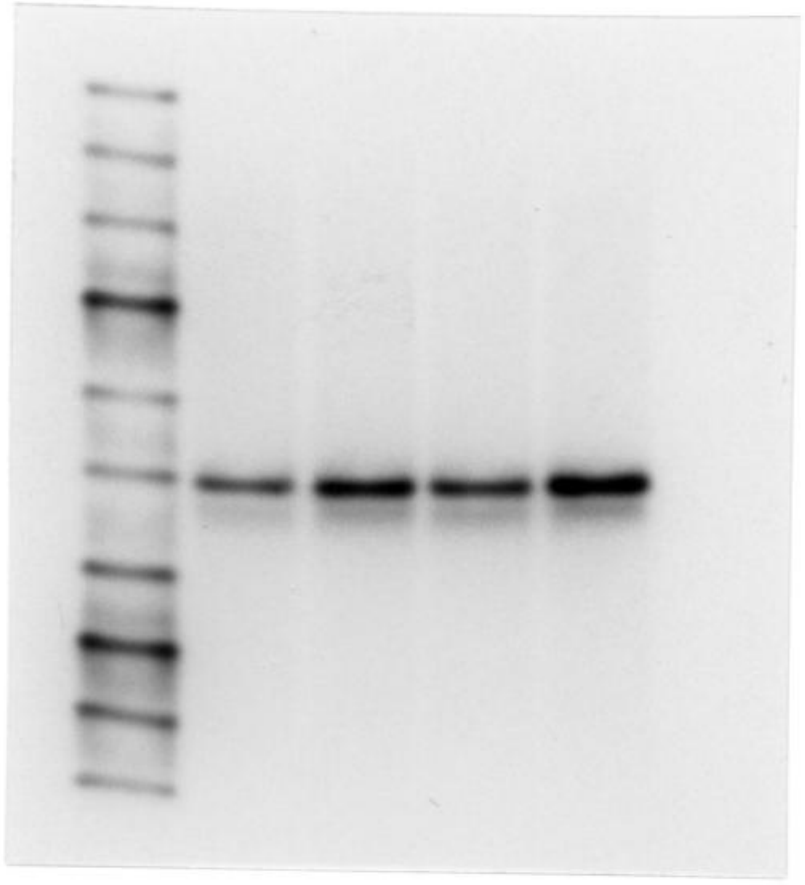

Fig 5U SELENOP

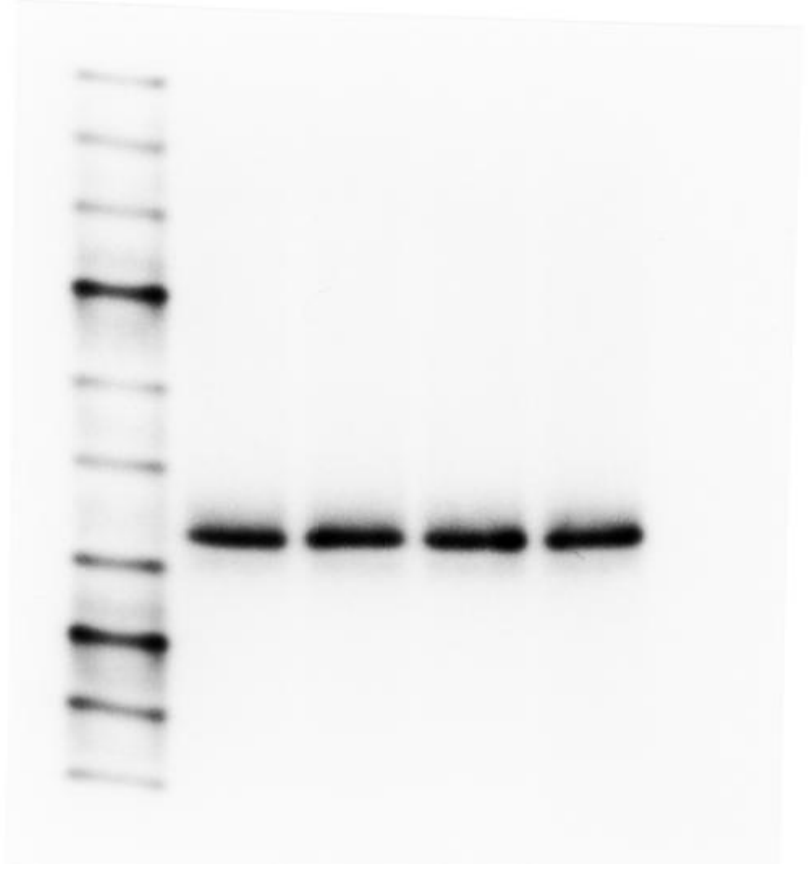

Fig 5U β-actin

Fig 6

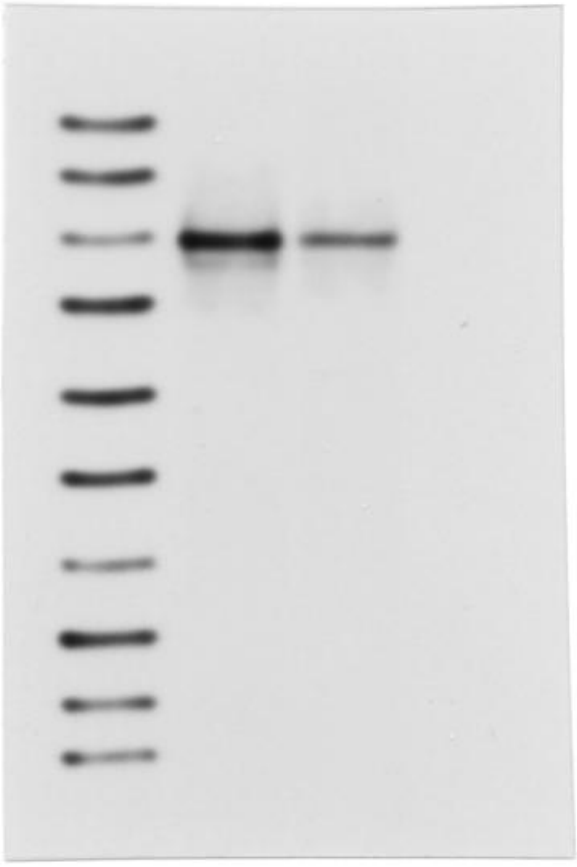

Fig 6M NRF2

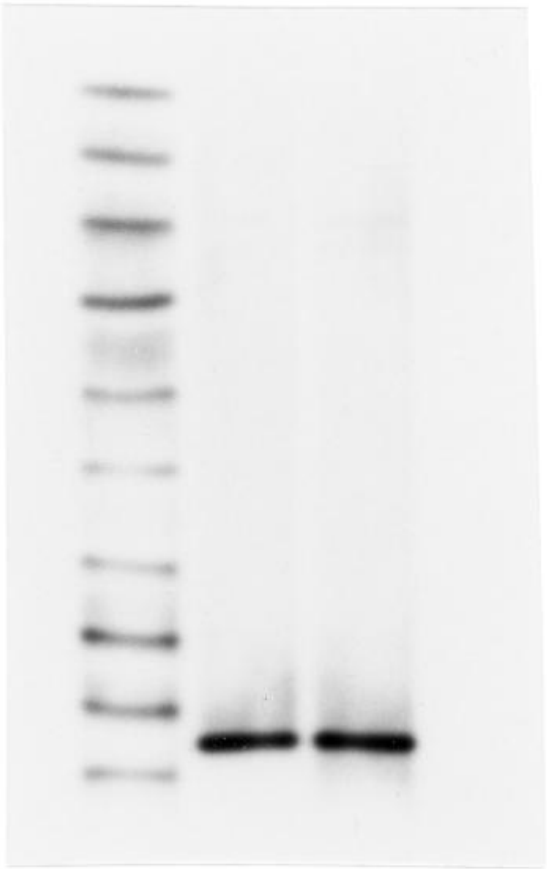

Fig 6M H3

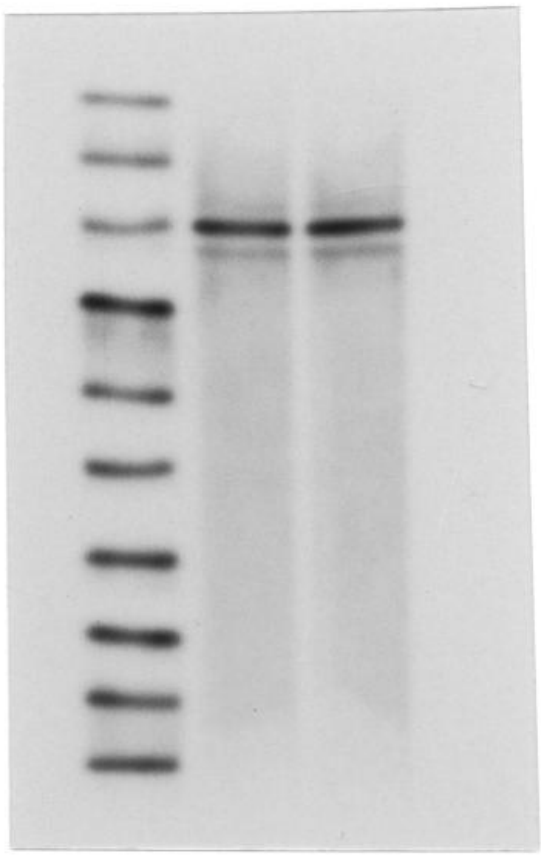

Fig 6M NRF2

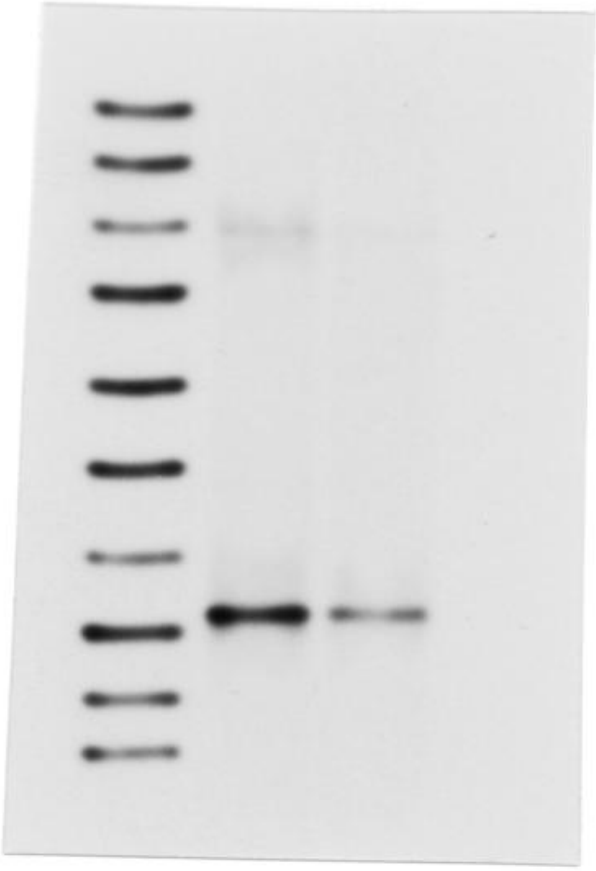

Fig 6M HO-1

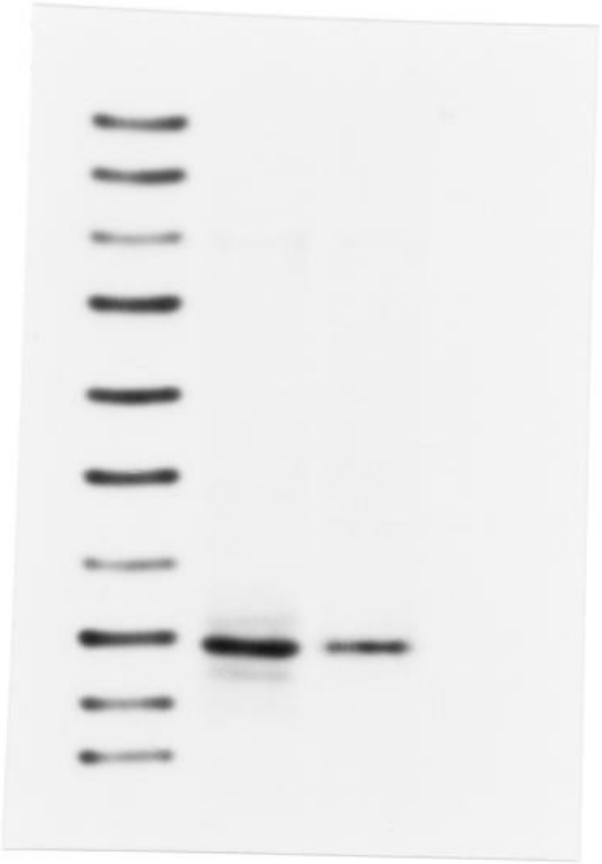

Fig 6M NQO-1

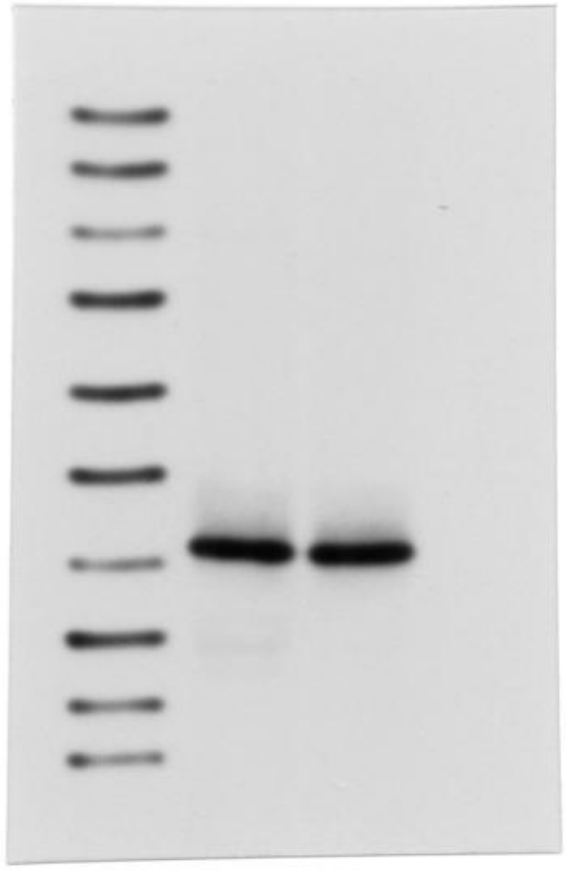

Fig 6M β-actin

Fig 7

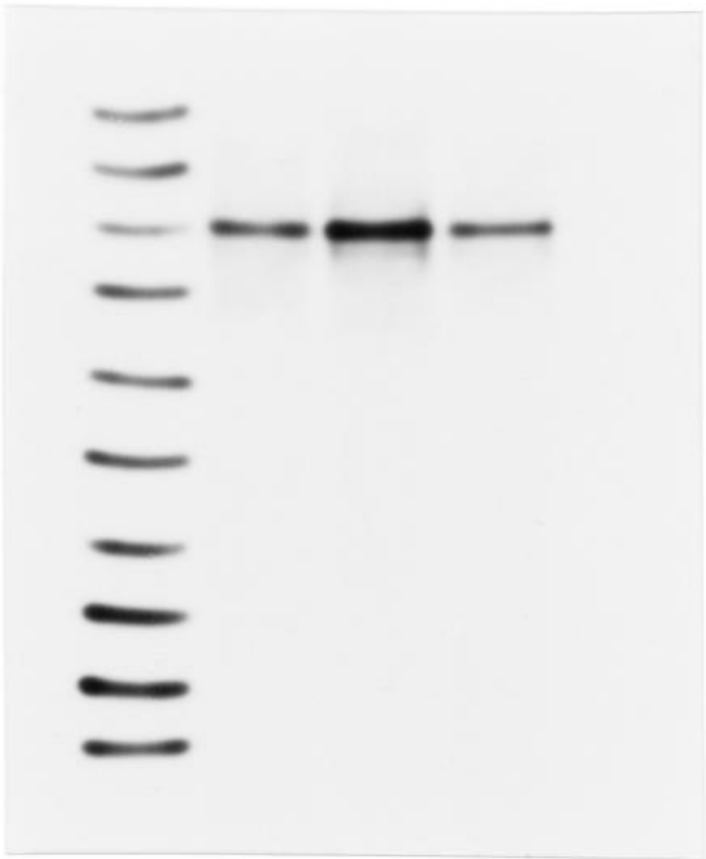

Fig 7J NRF2

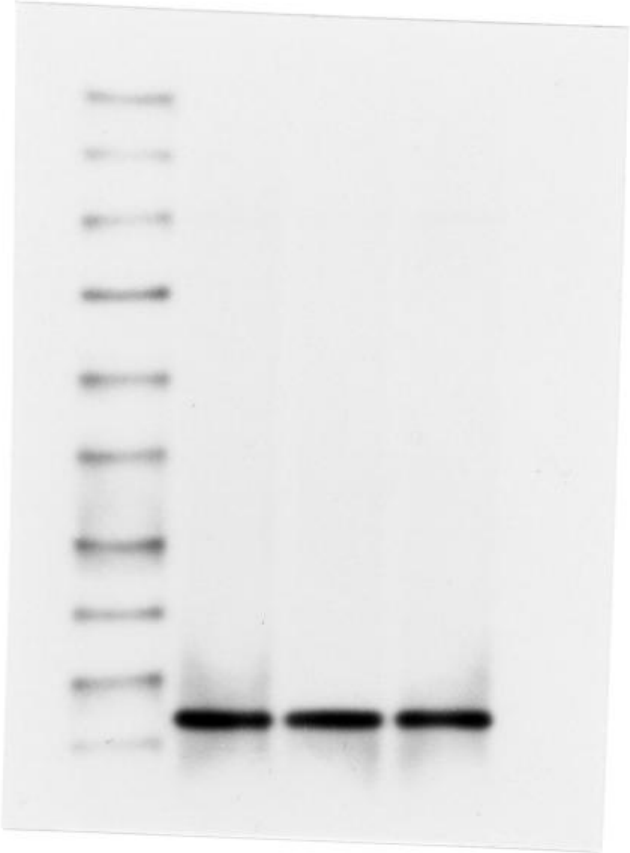

Fig 7J H3

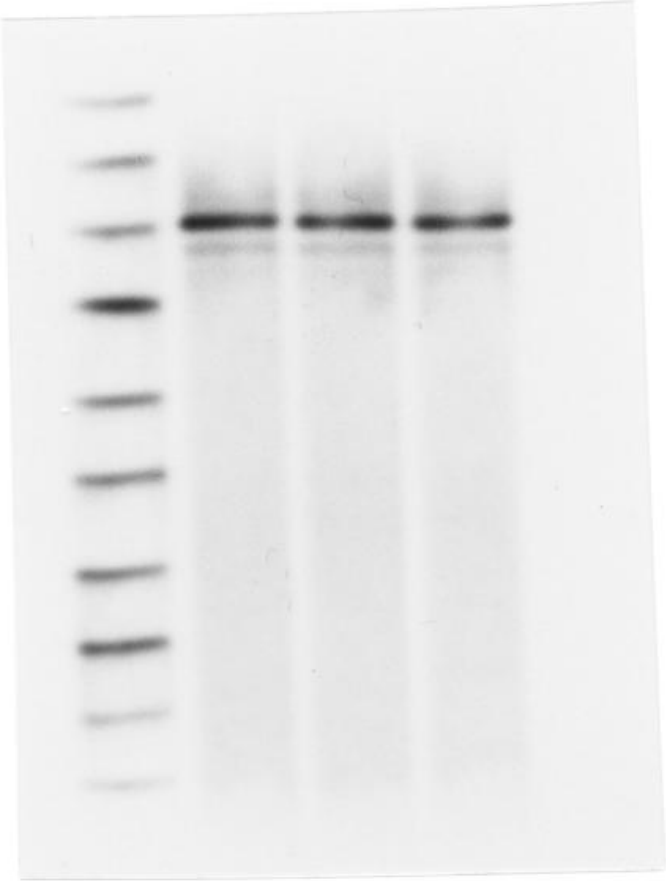

Fig 7J NRF2

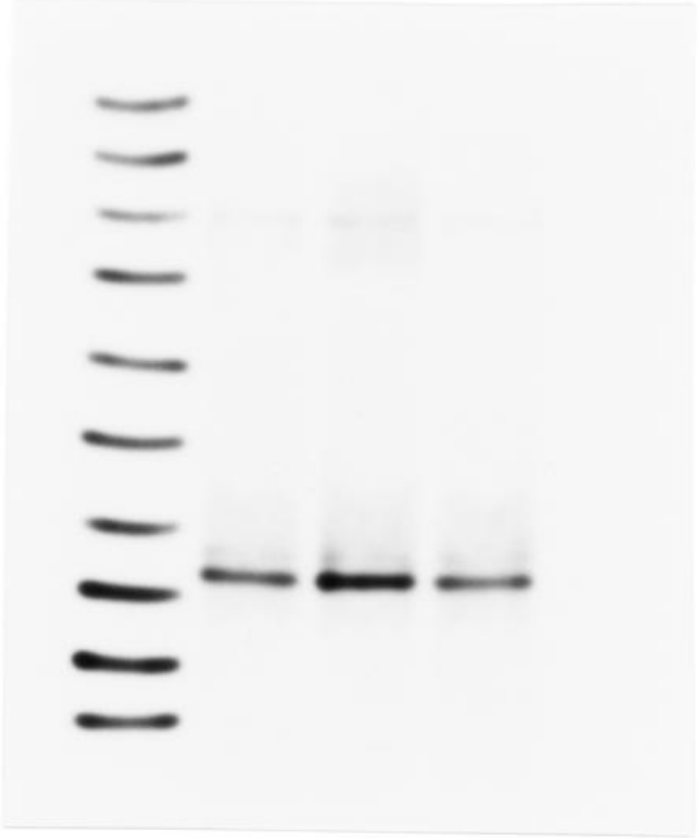

Fig 7J HO-1

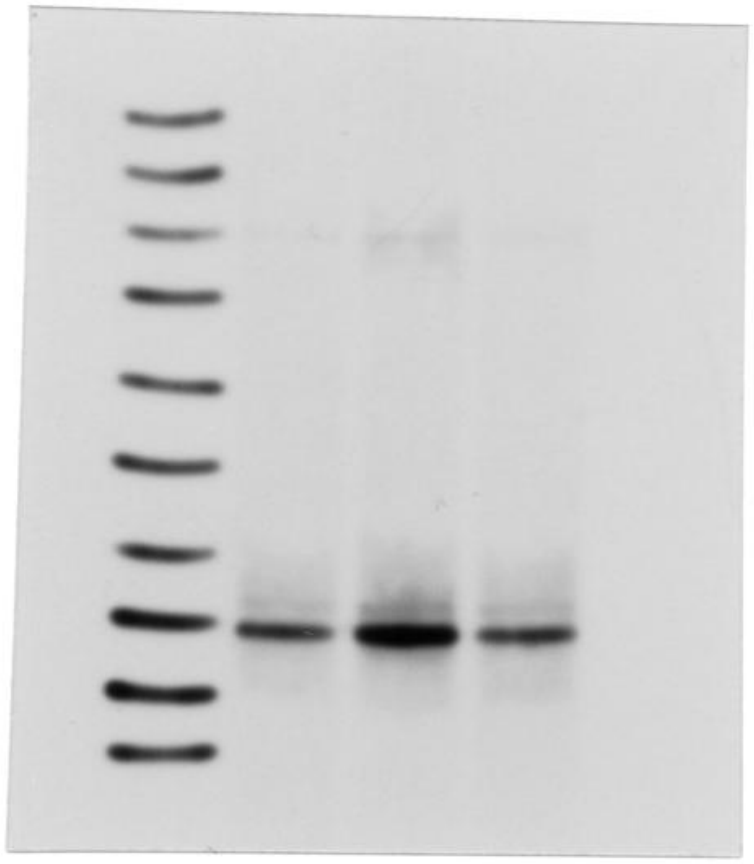

Fig 7J NQO-1

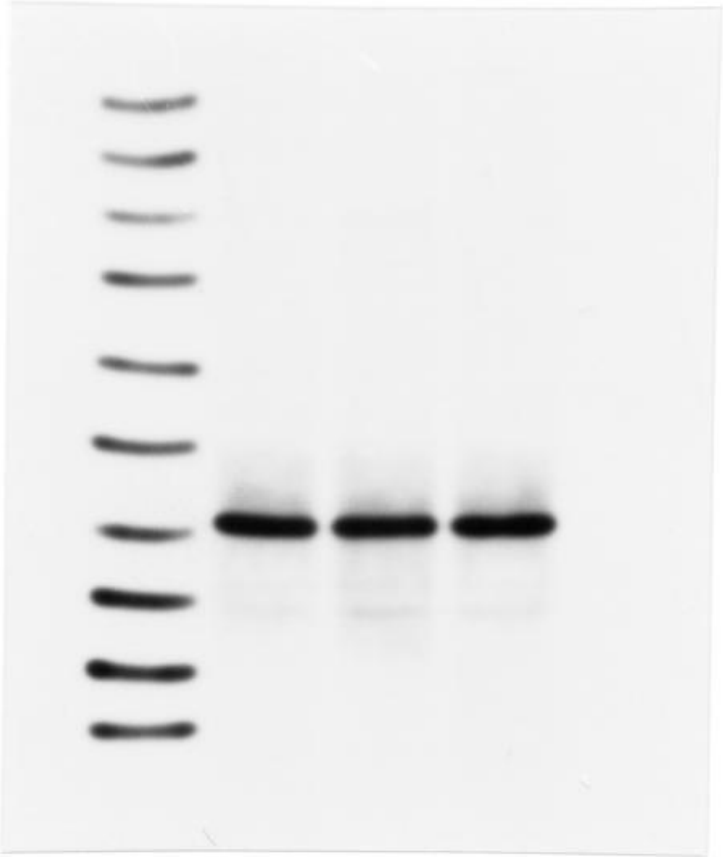

Fig 7J β-actin

Fig 8

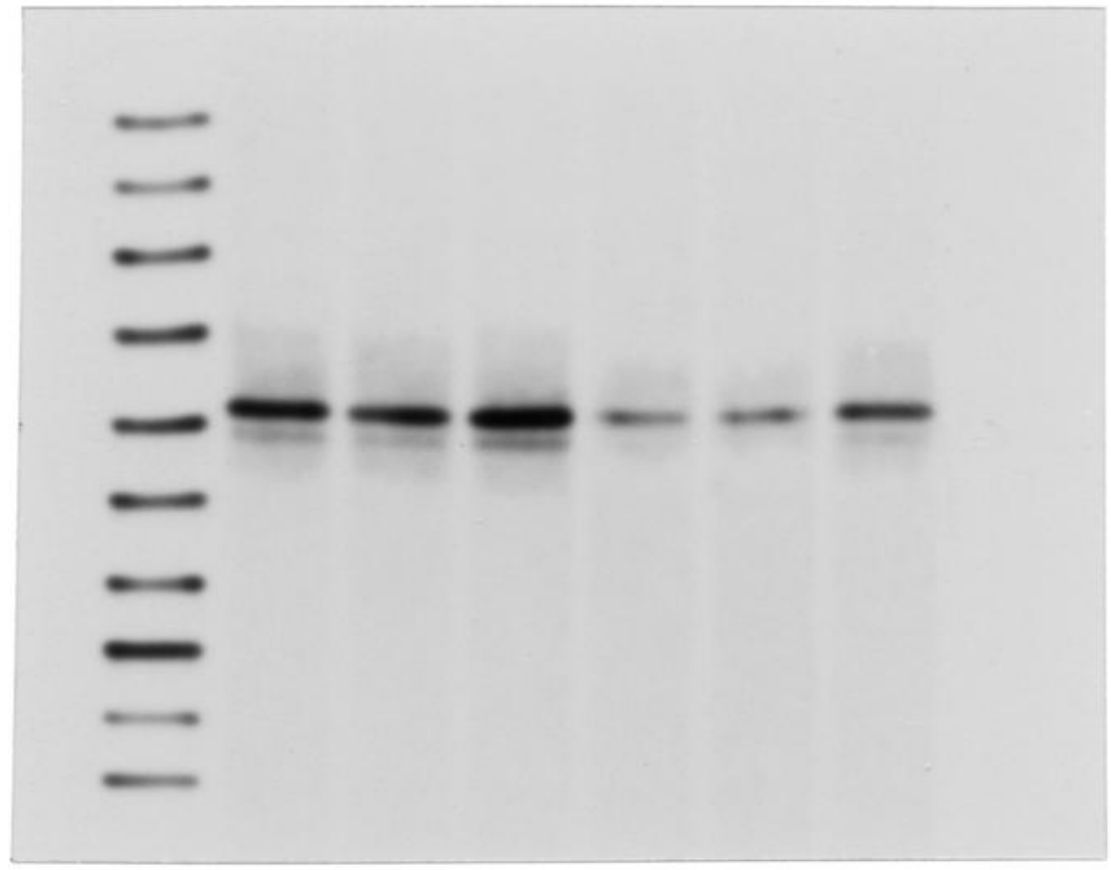

Fig 8N Occludin

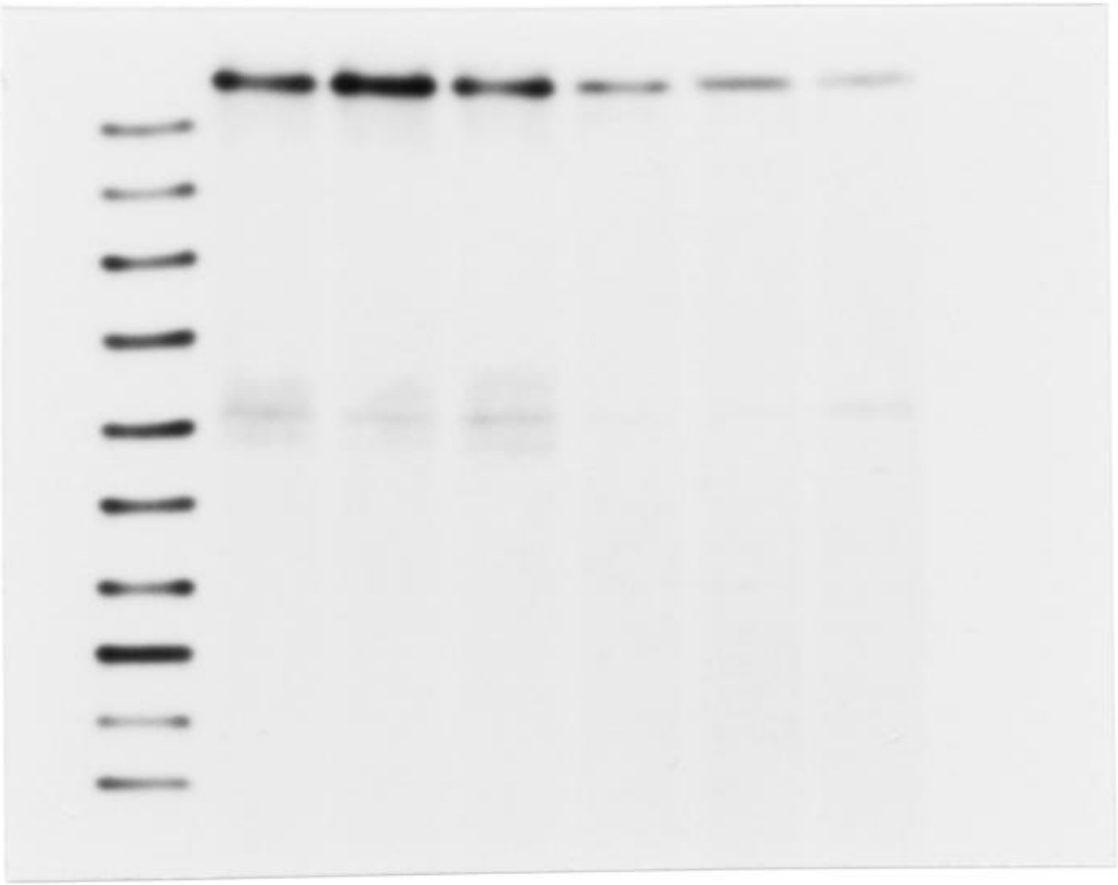

Fig 8N ZO-1

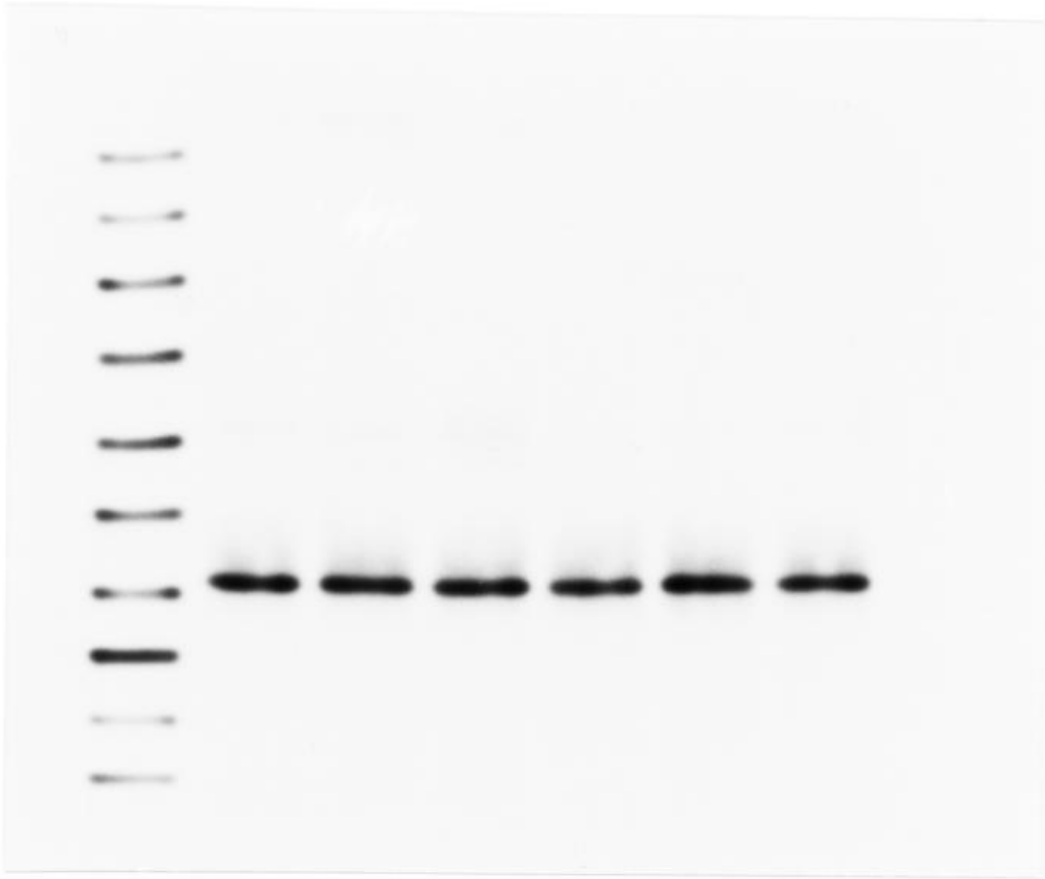

Fig 8N β-actin

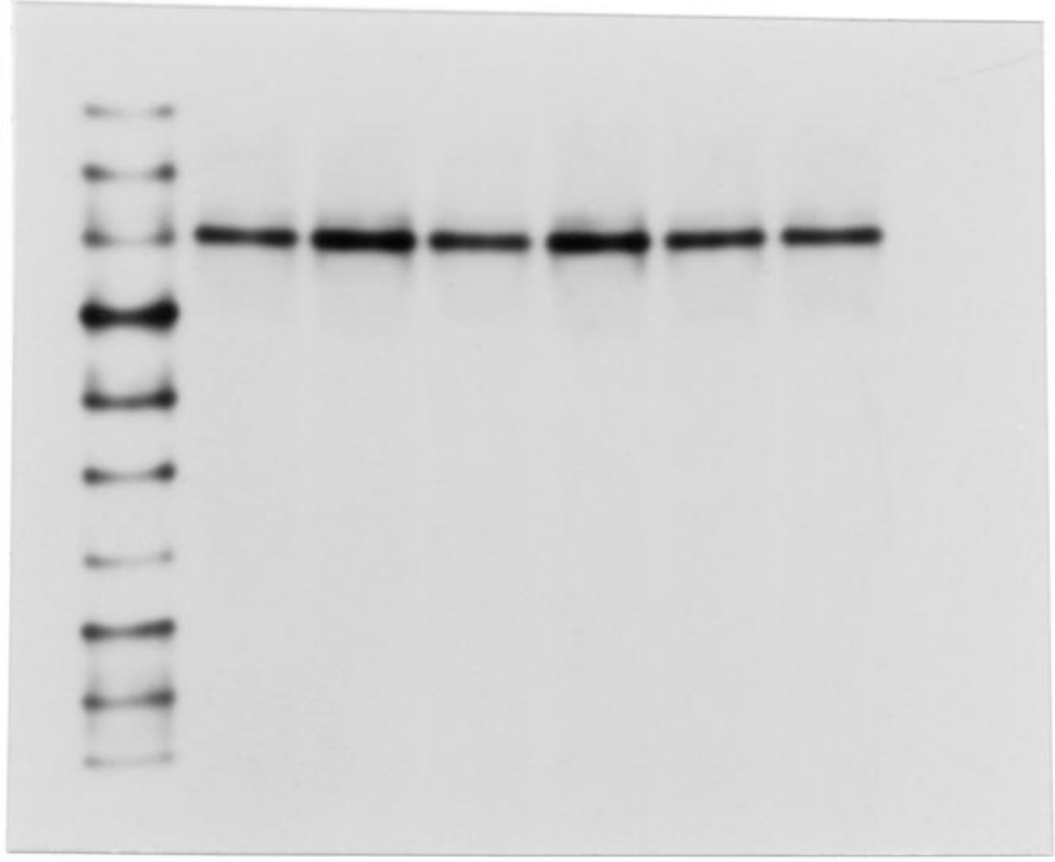

Fig 8R NRF2

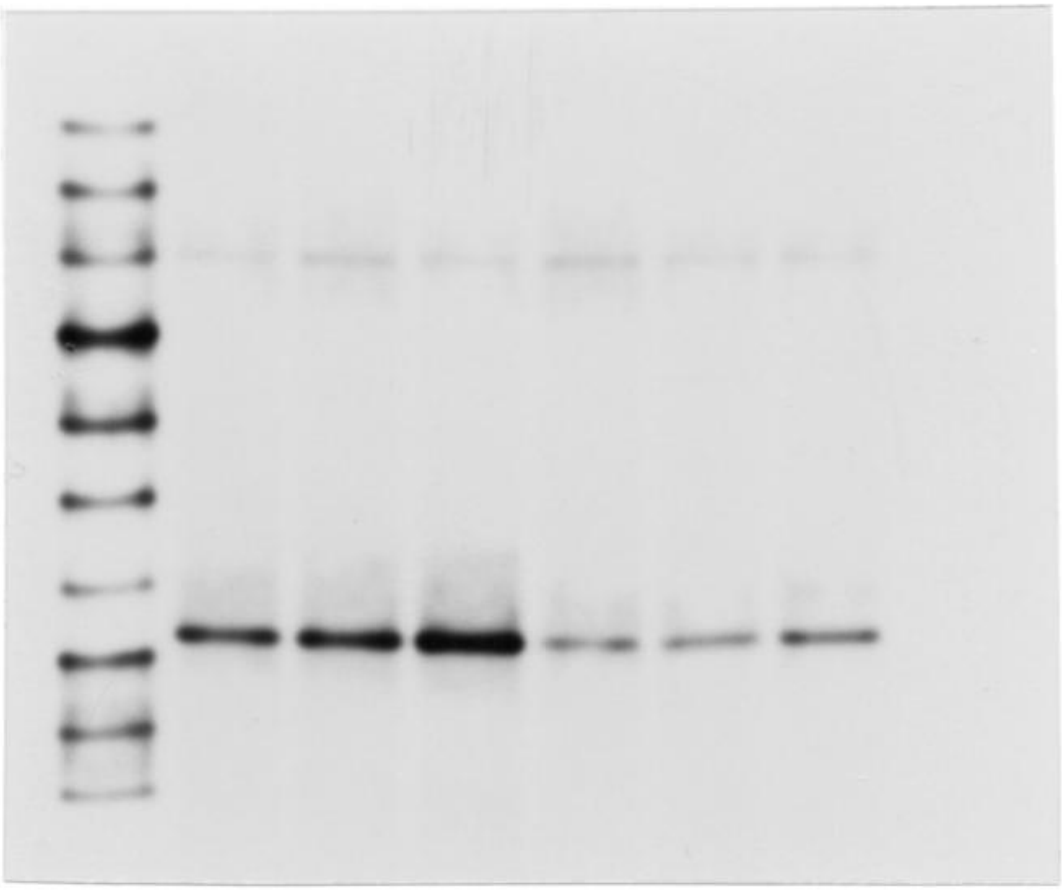

Fig 8R HO-1

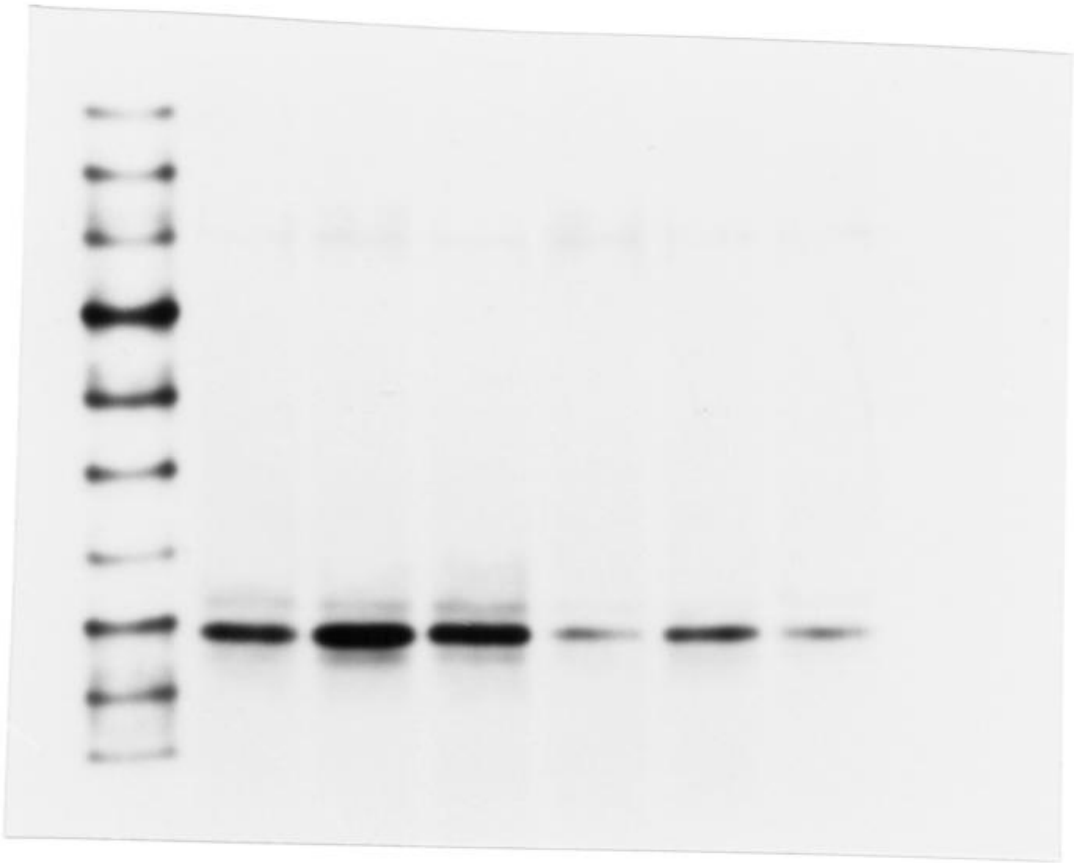

Fig 8R NQO-1

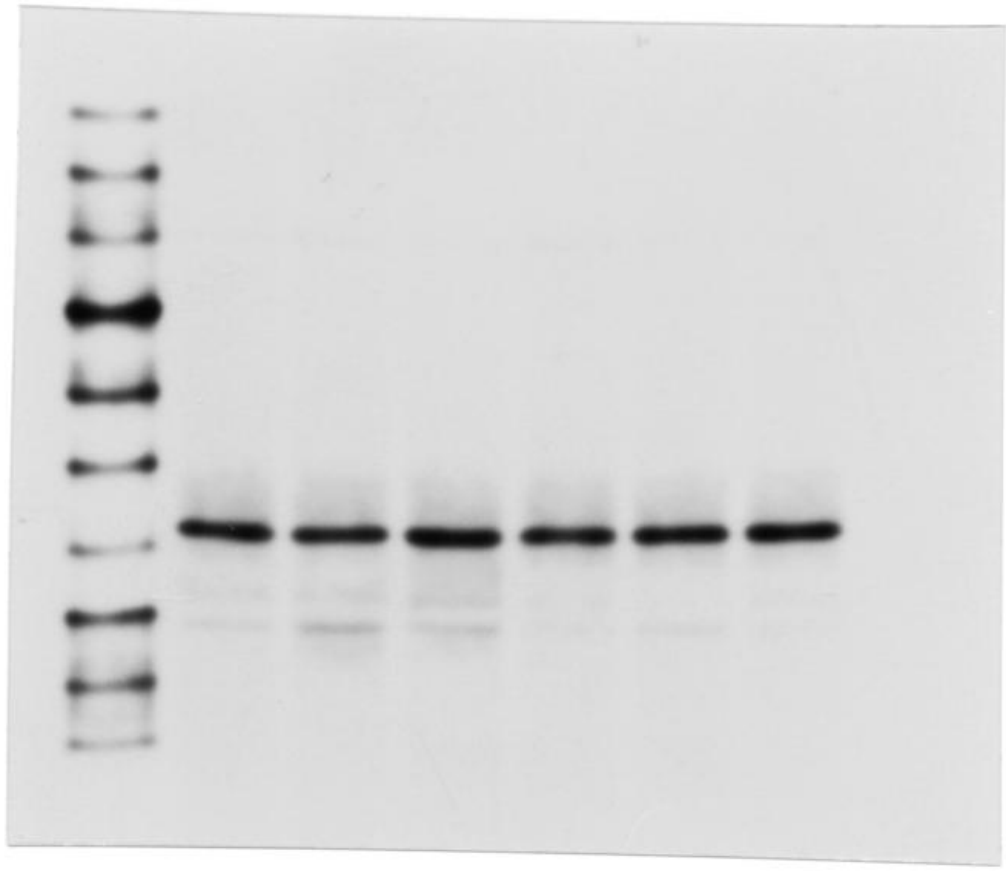

Fig 8R β-actin

Fig S8

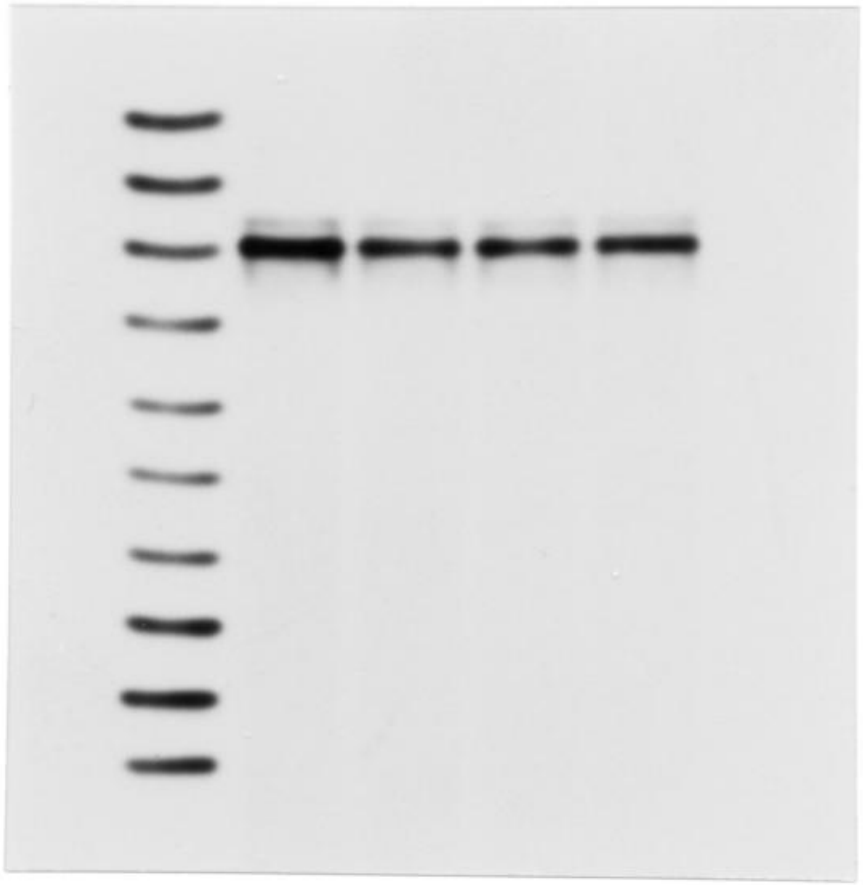

Fig S8A NRF2

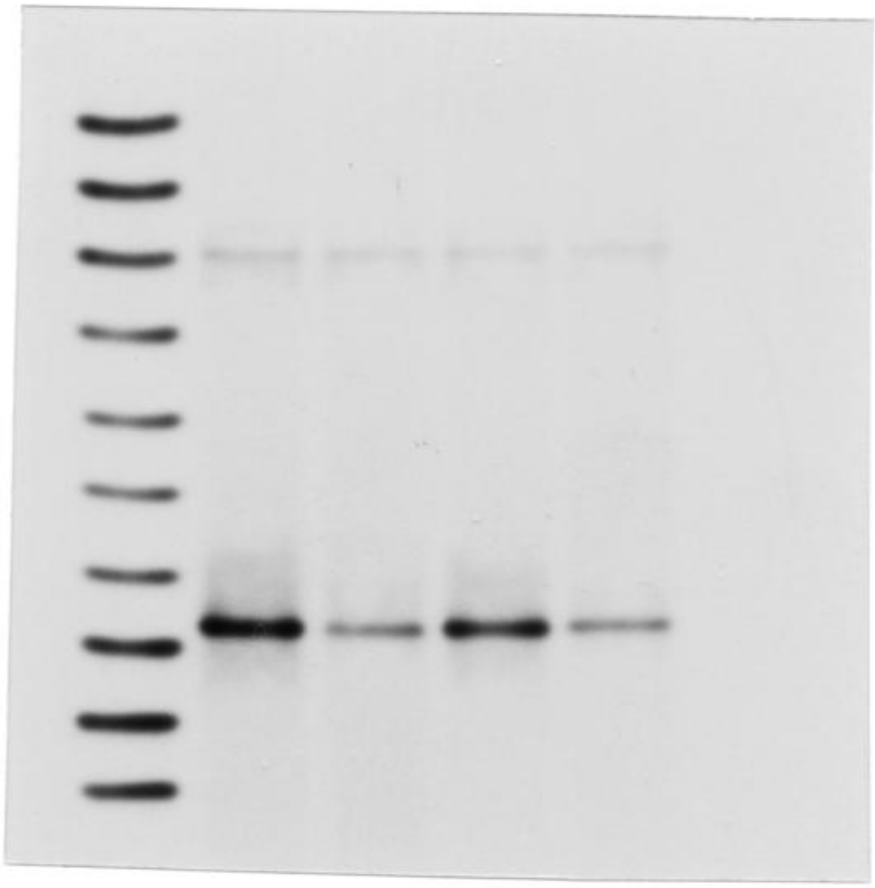

Fig S8A HO-1

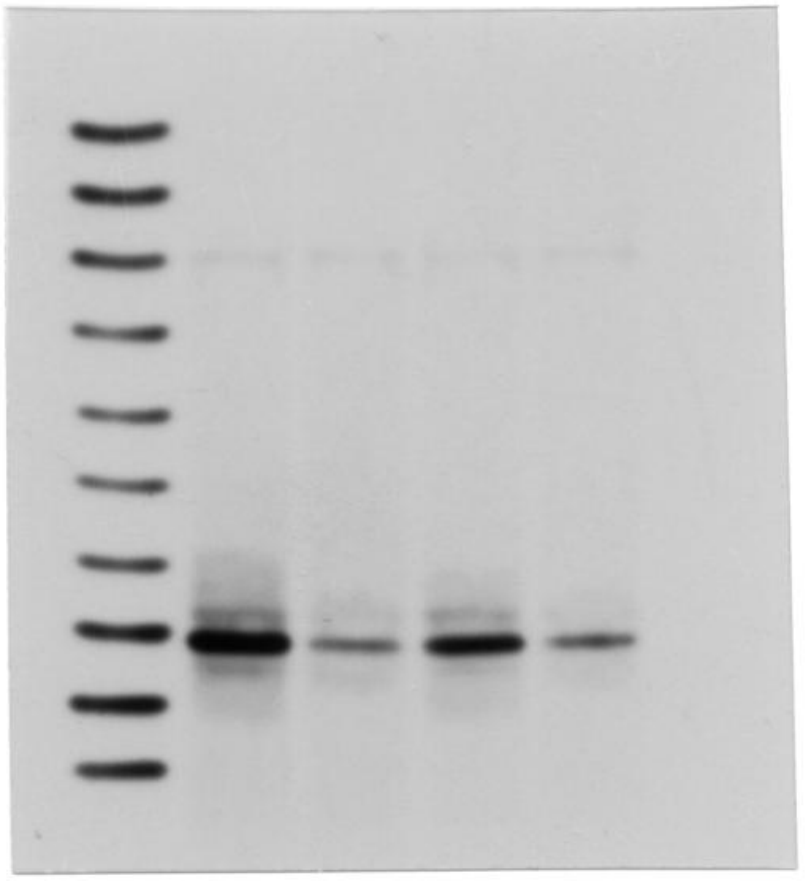

Fig S8A NQO-1

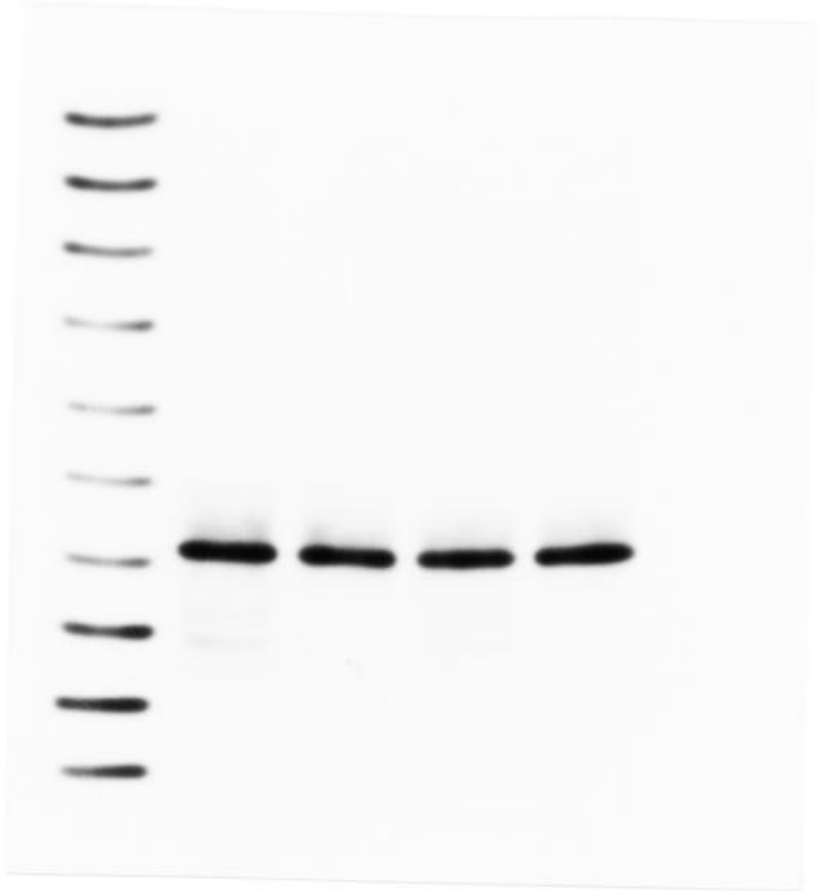

Fig S8A β-actin

Fig 9

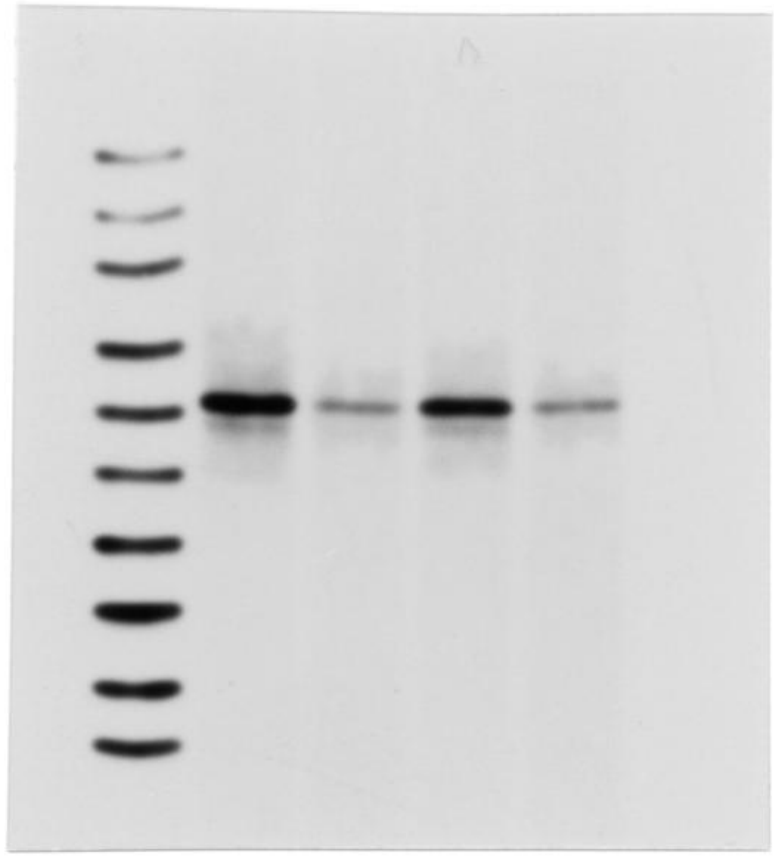

Fig 9O Occludin

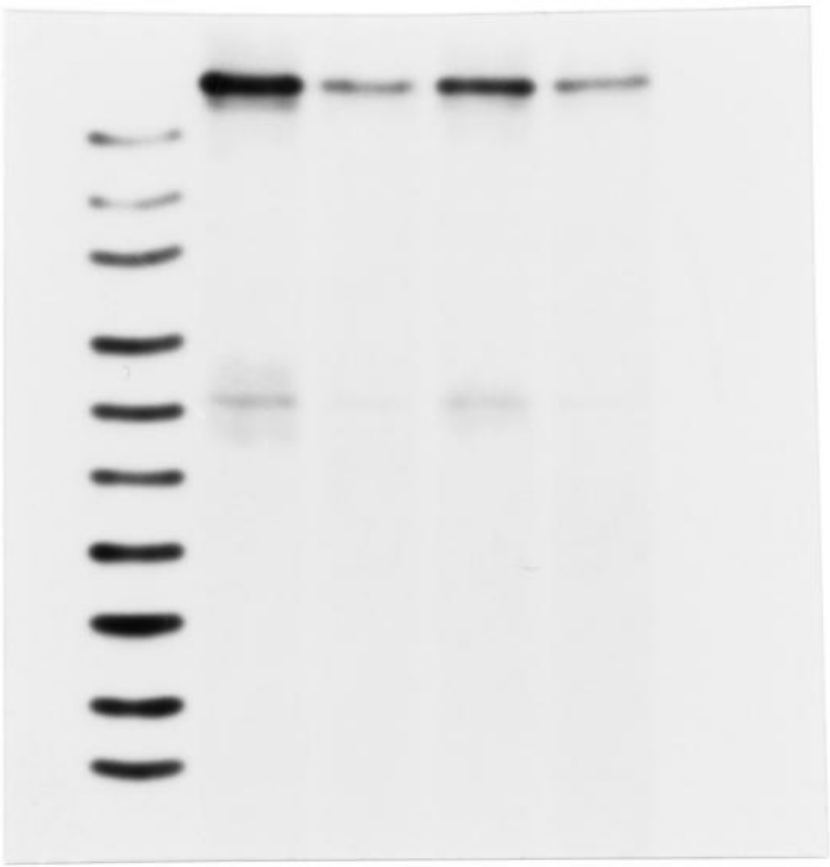

Fig 9O ZO-1

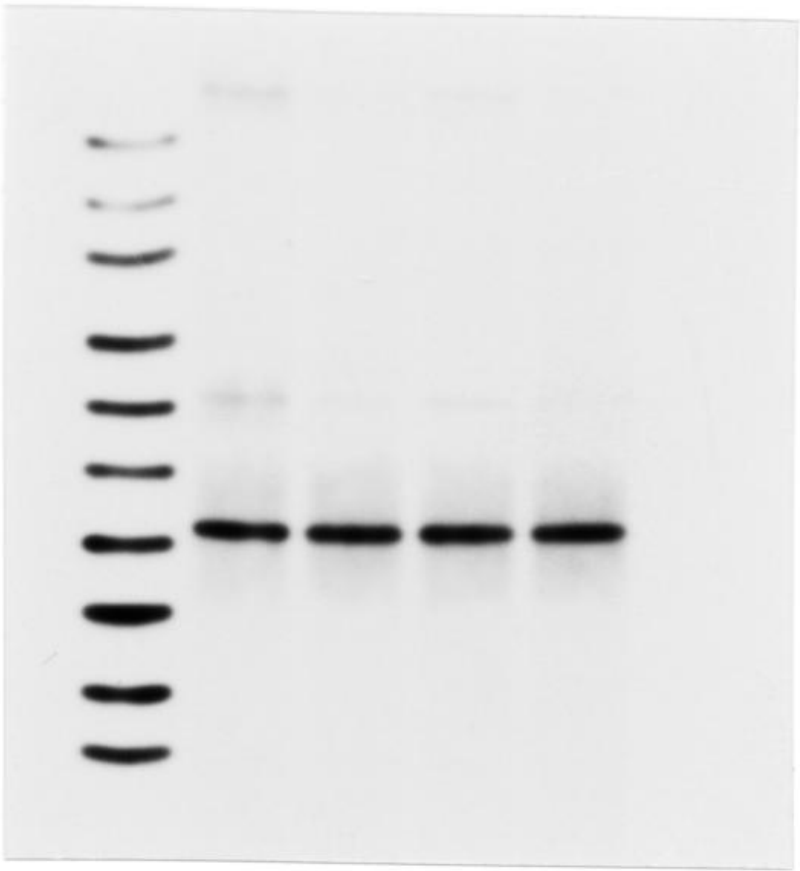

Fig 9O β-actin
