# Supplementary material for: Macrophage SELENOP controls neutrophil homeostasis through redox stress regulation in Crohn's disease
Source: Redox Biol. 2026 Sep 9;97:104377. doi: 10.1016/j.redox.2026.104377 (PMC13587815; doi:10.1016/j.redox.2026.104377)
Supplement: Supplementary file 2 [file mmc1.doc]

Table S1.	Antibody information

| Anti-Occludin | Abcam | ab216327 |
| --- | --- | --- |
| Anti-ZO-1 | Abcam | ab221547 |
| Anti-CD68 | Servicebio | GB113109 |
| Anti-F4/80 | Servicebio | GB113373 |
| Anti-SELENOP | Abcam | ab277526 |
| Annti-MPO | Servicebio | GB11224 |
| Anti-Cit-H3 | Abcam | AB281584 |
| Anti-NRF2 | Servicebio | GB113808 |
| Anti-HO-1 | Servicebio | GB15549 |
| Anti-NQO-1 | Servicebio | GB15282 |
| Anti-CAP1 | Invitrogen | MA5-53318 |
| Anti-NLRP3 | Servicebio | GB114320 |
| Anti-Caspase-1 | Abcam | ab207802 |
| Anti-GSDMD-N | Abcam | ab215203 |
| Anti-TRIP12 | Proteintech | 25303-1-AP |
| Anti-Ubiquitin | Cell Signaling Technology | 20326 |
| Anti-CREB1 | Servicebio | GB15236 |
| Anti-p-CREB1 | Abcam | ab220798 |
| Anti-SMARCA4 | Abcam | ab110641 |
| Anti-EpCAM | Servicebio | GB11274 |

ZO-1, zonula occludens-1; SELENOP, selenoprotein P; MPO, myeloperoxidase; Cit-H3, citrullinated histone H3; NRF2, nuclear factor erythroid 2–related factor 2;

HO-1, heme oxygenase-1; NQO-1, NAD(P)H quinone dehydrogenase 1; CAP1, cyclase-associated protein 1; NLRP3, NLR family pyrin domain containing 3; TRIP12, thyroid hormone receptor interactor 12; CREB1, cAMP response element-binding protein 1; SMARCA4, SWI/SNF-related matrix-associated actin-dependent regulator of chromatin subfamily A member 4; EpCAM, epithelial cell adhesion molecule.
