# Supplementary material for: Macrophage SELENOP controls neutrophil homeostasis through redox stress regulation in Crohn's disease
Source: Redox Biol. 2026 Sep 9;97:104377. doi: 10.1016/j.redox.2026.104377 (PMC13587815; doi:10.1016/j.redox.2026.104377)
Supplement: Supplementary file 3 [file mmc2.doc]

Table S2.	Patient demographic data

|  | Discovery set (scRNA) | Validation set |
| --- | --- | --- |
| Number | 4 | 56 |
| Age (year) | 42.0±2.2 | 43.1±6.0 |
| Gender, n (%) | - | - |
| Male | 3 (75.0) | 38(67.9) |
| Female | 1 (25.0) | 18(32.1) |
| Disease location, n (%) | - | - |
| Colonic | 2 (50.0) | 16(28.6) |
| Ileocolic | 2 (50.0) | 40(71.4) |
| Duration of disease (month) | 21.3±6.5 | 22.4±10.1 |
| Body mass index (kg/m^2^) | 20.9±0.9 | 21.6±2.0 |
| Recurrence within postoperative 1 year | 1 (25.0) | 22 (39.3) |
| CRP (mg/L) | 6.9±4.2 | 21.0±16.0 |
| CDAI | 118.3±46.3 | 128.4±53.3 |
| SES-CD | 4.8±1.5 | 4.8±1.8 |

scRNA-seq, single-cell RNA sequencing; CRP, C-reactive protein; CDAI, Crohn’s disease activity index; SES-CD, Simple Endoscopic Score for Crohn’s Disease. Data are expressed as means ± SD or number with percentage.
