# Supplementary material for: Macrophage SELENOP controls neutrophil homeostasis through redox stress regulation in Crohn's disease
Source: Redox Biol. 2026 Sep 9;97:104377. doi: 10.1016/j.redox.2026.104377 (PMC13587815; doi:10.1016/j.redox.2026.104377)
Supplement: Supplementary file 4 [file mmc3.doc]

Table S3. Primers sequences for qRT-PCR

| Name | Sequence (5′-3′) |
| --- | --- |
| Human SELENOP |  |
| Forward | CCAGCCTGGAGCATAAGAGATC |
| Reverse | GCAGTATGCACAGGTATCAGC |
| Human SESN2 |  |
| Forward | TCACCTACAATACCATCGCCAT |
| Reverse | CACCTCCCCATAATCATAGTCA |
| Human TRIP12 |  |
| Forward | CCCAAAGGCTAACACATCAGGA |
| Reverse | CAGAGAAAACATGCGAACCAC |
| Human ATF3 |  |
| Forward | GTCCATCACAAAAGCCGAGGT |
| Reverse | TTTCTGCAGGCACTCCGTCT |
| Human CREB1 |  |
| Forward | AGTGTAACTGATTCCCAAAAGCG |
| Reverse | TCTTCAATCCTTGGCACTCCT |
| Human IL-17A |  |
| Forward | ACATCCATAACCGGAATACCAA |
| Reverse | GTAGTCCACGTTCCCATCAGC |
| Human IL-6 |  |
| Forward | ATGTGTGAAAGCAGCAAAGAGG |
| Reverse | ATTTTCACCAGGCAAGTCTCC |
| Human SMARCA4 |  |
| Forward | TCTTCCCAAACTCCGAGCAAC |
| Reverse | TCGTGGTTCCATCAAGCCT |
| Human TNF-α |  |
| Forward | CCGACTCAGCGCTGAGATCA |
| Reverse | GCAATGATCCCAAAGTAGACCTG |
| Human IL-1β |  |
| Forward | TCTGTACCTGTCCTGCGTGT |
| Reverse | GGCAGACTCAAATTCCAGCT |
| Human IL-6 |  |
| Forward | GCCACTCACCTCTTCAGAACG |
| Reverse | TGCTGCTTTCACACATGTTACTC |
| Human GAPDH |  |
| Forward | TCCTGGGCTACACTGAGCAC |
| Reverse | CTGTTGCTGTAGCCAAATTCGTTG |
| ChIP-qPCR |  |
| Site1 |  |
| Forward | CTGGTAACGCTTGTAAGTTGG |
| Reverse | ATTCAGCATACAGCAATCAGTGG |
| Site2 |  |
| Forward | AGATATTCCTCCCTGTAGTTCAT |
| Reverse | AACTACTGGGTTTAGGCCAGG |
| Mouse IL-1β |  |
| Forward | GGAGTTTGAGTCTGCAGAGTTC |
| Reverse | AAGACACGGATTCCATGGTGA |
| Mouse TNF-α |  |
| Forward | ATCAGTTCTATGGCCCAGACC |
| Reverse | GTCTTTGAGATCCATGCCGTT |
| Mouse IL-17A |  |
| Forward | AGACTACCTCAACCGTTCCAC |
| Reverse | CTTTCCCTCCGCATTGACACA |

qRT-PCR, quantitative real time-polymerase chain reaction; SELENOP, Selenoprotein P; SESN2, Sestrin 2; TRIP12, Thyroid Hormone Receptor Interactor 12; ATF3, Activating Transcription Factor 3; CREB1, cAMP Response Element-Binding Protein 1; IL-1β, interleukin-1 beta; TNF-α, tumor necrosis factor alpha; ChIP-qPCR, Chromatin immunoprecipitation quantitative PCR; IFN-γ, Interferon gamma.
