# Supplementary material for: Macrophage SELENOP controls neutrophil homeostasis through redox stress regulation in Crohn's disease
Source: Redox Biol. 2026 Sep 9;97:104377. doi: 10.1016/j.redox.2026.104377 (PMC13587815; doi:10.1016/j.redox.2026.104377)
Supplement: Supplementary file 5 [file mmc4.doc]

Table S4. Primers for sh- and si-RNA sequences

| Name | Sequence (5′-3′) |
| --- | --- |
| Human sh-SELENOP-1 |  |
| Forward | CCGG CATCATCACAATCATGGACAT CTCGAG ATGTCCATGATTGTGATGATG TTTTTG |
| Reverse | AATTCAAAAA CATCATCACAATCATGGACAT CTCGAG ATGTCCATGATTGTGATGATG |
| Human sh-SELENOP-2 |  |
| Forward | CCGG GTGAGAATGACCTTCAAACTA CTCGAG TAGTTTGAAGGTCATTCTCAC TTTTTG |
| Reverse | AATTCAAAAA GTGAGAATGACCTTCAAACTA CTCGAG TAGTTTGAAGGTCATTCTCAC |
| Human sh-SELENOP-3 |  |
| Forward | CCGG GACAAATAAGTCAGCAGCTTA CTCGAG TAAGCTGCTGACTTATTTGTC TTTTTG |
| Reverse | AATTCAAAAA GACAAATAAGTCAGCAGCTTA CTCGAG TAAGCTGCTGACTTATTTGTC |
| Human si-CREB1-1 | Target: AAGCTGAAAACCAACAAATGACA |
| Forward | UCAUUUGUUGGUUUUCAGCUU |
| Reverse | GCUGAAAACCAACAAAUGACA |
| Human si-CREB1-2 | Target: GTCCAAACAGTTCAGATTTCAAC |
| Forward | UGAAAUCUGAACUGUUUGGAC |
| Reverse | CCAAACAGUUCAGAUUUCAAC |
| Human si-CREB1-3 | Target: TCCAAACAGTTCAGATTTCAACT |
| Forward | UUGAAAUCUGAACUGUUUGGA |
| Reverse | CAAACAGUUCAGAUUUCAACU |
| Human si-TRIP12-1 | Target: GAGTAGAAAATCTAATTCTAAGG |
| Forward | UUAGAAUUAGAUUUUCUACUC |
| Reverse | GUAGAAAAUCUAAUUCUAAGG |
| Human si-TRIP12-2 | Target: GCCAAATTAGCAAGTTTAAGAAA |
| Forward | UCUUAAACUUGCUAAUUUGGC |
| Reverse | CAAAUUAGCAAGUUUAAGAAA |
| Human si-TRIP12-3 | Target: AGCACAATTTTGATATTATGAAC |
| Forward | UCAUAAUAUCAAAAUUGUGCU |
| Reverse | CACAAUUUUGAUAUUAUGAAC |
| Human si-SMARCA4-1 | Target: CCGAATTCAGGAACTTGAAAACC |
| Forward | UUUUCAAGUUCCUGAAUUCGG |
| Reverse | GAAUUCAGGAACUUGAAAACC |
| Human si-SMARCA4-2 | Target: AAGAAAATTACAGGAAAAGATAT |
| Forward | AUCUUUUCCUGUAAUUUUCUU |
| Reverse | GAAAAUUACAGGAAAAGAUAU |
| Human si-SMARCA4-3 | Target: ACCAAGAAGATGAAGAAGATTGT |
| Forward | AAUCUUCUUCAUCUUCUUGGU |
| Reverse | CAAGAAGAUGAAGAAGAUUGU |

SELENOP, Selenoprotein P; TRIP12, Thyroid Hormone Receptor Interactor 12; CREB1, cAMP Response Element-Binding Protein 1;
